# Hepatic ENTPD5 Is Critical for Maintaining Metabolic Homeostasis and Promoting Brown Adipose Tissue Thermogenesis

**DOI:** 10.1002/advs.202503603

**Published:** 2025-08-11

**Authors:** Rufeng Ma, Song Hou, Rui Xiang, Wenjun Liu, Xin Li, Yujing Chi, Ming Xu, Haochen Yao, Jing Li, Jichun Yang

**Affiliations:** ^1^ Department of Physiology and Pathophysiology School of Basic Medical Sciences State Key Laboratory of Vascular Homeostasis and Remodeling Center for Non‐coding RNA Medicine Peking University Health Science Center Beijing 100191 China; ^2^ Department of Central Laboratory and Institute of Clinical Molecular Biology Department of Gastroenterology Peking University People's Hospital Beijing 100044 China; ^3^ Department of Cardiology Peking University Third Hospital Beijing 100191 China; ^4^ Hepatobiliary and Pancreatic Surgery Department General Surgery Center First Hospital of Jilin University No.1 Xinmin Street Changchun Jilin 130021 China; ^5^ Department of Endocrinology Beijing Chao‐Yang Hospital Capital Medical University Beijing 100020 China

**Keywords:** adenosine triphosphate metabolism, adrenomedullin, brown adipose tissue thermogenesis, ectonucleoside triphosphate diphosphohydrolase 5

## Abstract

Although hepatocyte‐released adenosine triphosphate (ATP) plays important roles in maintaining metabolic homeostasis, how its hydrolyzation outside hepatocytes impacts on glucolipid metabolism remains unclear. The authors aim to identify the enzyme(s) that hydrolyzes hepatocyte‐secreted ATP to regulate metabolic homeostasis. All known ATP‐hydrolyzing enzymes are expressed with the highest expression of ectonucleoside triphosphate diphosphohydrolase 5 (ENTPD5) in hepatocytes. ENTPD5 expression is reduced in steatotic mouse and human livers. Hepatic ENTPD5 overexpression ameliorates the deregulated glucolipid metabolism and obesity with increased brown adipose tissue (BAT) thermogenesis, while hepatic ENTPD5 silencing exerted the opposite effects in obese mice. Mechanistically, ENTPD5 hydrolyzes extracellular ATP to ADP, which activates purinergic receptor, P2Y_12_, to inhibit gluconeogenesis and lipid deposition, and repress adrenomedullin (ADM) expression. Hepatic ENTPD5 repression promotes ADM expression and release to inhibit uncoupling protein 1 (UCP1) expression and thermogenesis in BAT. Hepatic ADM expression is increased in NAFLD patients. Serum ADM level is correlated positively with Body mass index in overweighted or obese humans. Hepatic ADM silencing promotes UCP1 expression and thermogenesis in BAT of obese mice. Overall, ENTPD5‐mediated hydrolysis of extracellular ATP to ADP of hepatocytes is critical for maintaining hepatic glucose/lipid metabolism and promoting BAT thermogenesis by inhibiting ADM expression and secretion.

## Graphical abstract

ENTPD5‐mediated conversion of extracellular adenosine triphosphate to ADP of hepatocytes is critical for maintaining hepatic glucose/lipid metabolism and promoting brown adipose tissue thermogenesis via the inhibition of adrenomedullin expression and secretion. Inhibition of hepatic ENTPD5 expression plays important roles in the development and progression of diabetes, NAFLD, and obesity.

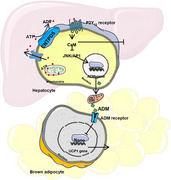

## 1 Introduction

The global prevalence of diabetes and obesity has reached epidemic levels, affecting billions of adults worldwide and become significant public health challenges.^[^ ^1^, ^2^, ^3^, ^4^ ^]^ Brown adipose tissue (BAT) is present in adult humans, particularly in the neck and clavicular regions, and plays important metabolic roles.^[^ ^5^ ^]^ Activating BAT thermogenesis to boost energy expenditure and improve metabolic health is a promising strategy for combating metabolic diseases.^[^ ^4^ ^]^ Current approaches include cold exposure, exercise, medications, and dietary changes, but long‐term adherence and potential side effects limit their effectiveness.^[^ ^6^, ^7^, ^8^ ^]^ Clearly, further research on the regulatory mechanism of BAT thermogenesis will shed light on the prevention and treatment of obesity and metabolic diseases.

The liver is crucial for energy metabolism and regulate inter‐organ crosstalk by secreting hepatokines such as fibroblast growth factor 21 to influence lipid storage, lipolysis, fatty acid oxidation, and glucose metabolism.^[^ ^9^, ^10^ ^]^ Meanwhile, liver is involved in regulating thermogenesis by releasing factors or neurotransmitters that influence BAT function. For example, liver‐derived acylcarnitines provide fuel for BAT during cold exposure, and activin E and the hepatokine tsukushi also affect BAT thermogenesis.^[^ ^11^, ^12^ ^]^ Liver‐derived factors can also influence thermogenesis through the vagus nerve.^[^ ^13^ ^]^ Recent studies have shown that dysregulation of adenosine triphosphate (ATP) production in the liver is linked to obesity and metabolic diseases in both humans and animals.^[^ ^14^, ^15^ ^]^ We and others have shown that family with sequence similarity 3 member A (FAM3A) plays in important roles in regulating hepatic and global metabolism by promoting ATP production and secretion.^[^ ^16^, ^17^, ^18^, ^19^, ^20^, ^21^ ^]^ We have recently demonstrated that FAM3A primarily stimulates ATP secretion via pannexin 1 to influence hepatic and global glucose/lipid metabolism.^[^ ^22^ ^]^

It is widely recognized that secreted ATP serves as a crucial signaling molecule in various cell types. Generally, extracellular ATP (eATP) is broken down into extracellular adenosine diphosphate (eADP), extracellular adenosine monophosphate (eAMP), and adenosine by certain enzymes on the cell membrane. So far, several types of enzymes such as ectonucleoside triphosphate diphosphohydrolases (ENTPDs), ectonucleotide pyrophosphatase/phosphodiesterase, ecto‐5′‐nucleotidase (Nte5, also known as CD73) and adenosine deaminase (Ada) have been shown to degrade ATP, ADP, and AMP, respectively.^[^ ^23^, ^24^ ^]^ ENTPDs regulate many biological processes in various cell types by modulating eATP and eADP homeostasis and balance. By modulating the concentrations of eATP and eADP, ENTPDs influence the activation of P2 purinergic receptors to regulate various immune cell responses and vascular functions.^[^ ^25^, ^26^, ^27^ ^]^ It had been previously reported that under stressed or injured conditions, hepatocyte released pathological level of ATP (µm to mm), which was metabolized by ENTPDs on macrophages present in the liver.^[^ ^28^, ^29^ ^]^ However, whether hepatocyte expresses ENTPDs remains unclear. Given that hepatocyte‐released physiological level of ATP (nm) is vital for maintaining glucose and lipid homeostasis in the liver, it is reasonable to speculate that hepatocyte should be capable of metabolizing eATP to maintain metabolic homeostasis.

The current study found that ENTPDs were ubiquitously expressed in hepatocytes with the highest expression of ENTPD5. Moreover, ENTPD5 was the only deregulated enzymes in steatotic mouse and human liver tissue, and hepatocytes treated with free fatty acids (FFAs). The study then investigated how ENTPD5 influenced glucose and lipid metabolism in the liver, with a focus on its impact on BAT thermogenesis.

## 2 Results

### 2.1 ENTPD5 Expression Was Reduced in Steatotic Mouse and Human Livers

**Figure 1:**
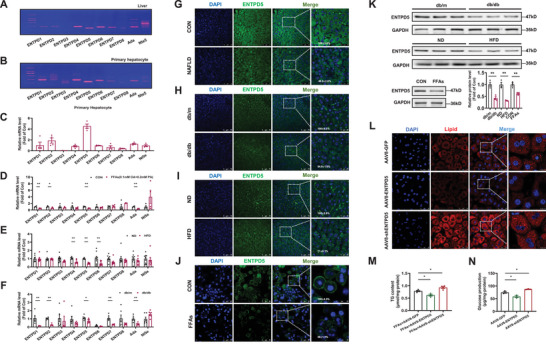
ENTPD5 expression is reduced in steatotic mouse and human livers, and in cultured hepatocytes treated with free fatty acids. A,B) Gel electrophoresis of PCR products determination the expression profile of ENTPDs in mouse livers (A) and cultured hepatocytes (B). C) Relative mRNA levels of ENTPDs in cultured mouse hepatocytes (*N* = 4). D–F) Changes in ENTPD mRNAs in primary hepatocytes treated with FFAs (D, *N* = 4), and HFD‐fed and db/db mouse livers (E,F, *N* = 5–8). Hepatocytes were treated with FFAs (0.1 mm oleic acid and 0.2 mm palmitic acid) for 24 h before assays. G) Confocal imaging analysis revealed that ENTPD5 protein was reduced in the livers of patients with NAFLD (*N* = 3). Scale bar, 75 and 25 µm. The relative expression data was marked in the images. H–J) Confocal imaging analysis indicated that ENTPD5 protein was reduced in the livers of obese mice (H,I), and primary hepatocytes treated with FFAs (J). Scale bar: 75 and 25 µm for (H,I); 50 and 25 µm for (J). The relative expression data was marked in the images (*N* = 3). K) Immunoblotting assays confirmed the reduction of ENTPD5 protein in obese mouse livers and FFAs‐treated hepatocytes (*N* = 3). L,M) The lipid staining (L) and TG quantification (M) in mouse primary hepatocytes infected with AAV8‐GFP, AAV8‐ENTPD5, or AAV8‐shENTPD5, respectively, in the presence of FFAs for 48 h (*N* = 4). Scale bar, 50 and 10 µm. N) Glucose production assays in mouse primary hepatocytes infected with AAV8‐GFP, AAV8‐ENTPD5, or AAV8‐shENTPD5, respectively, for 48 h (*N* = 3). Hepatocytes were infected with AAV8 at the dose of 100 vg per cell. FFAs (0.1 mm oleic acid (OA) + 0.2 mm palmitic acid (PA)); ND, normal diet; HFD, high fat diet. db/m, db/m mice, db/db, db/db mice. TG, triglycerides. *P < 0.05, ** *P* < 0.01 versus control mice.

The mRNA levels of ten known ATP‐hydrolyzing enzymes, including ENTPD1‐8, Ada, and Nte5, were ubiquitously expressed in both mouse livers and cultured primary mouse hepatocytes with the highest expression level of ENTPD5 (**Figure** **1**A–C). ENTPD 1, ENTPD2, ENTPD5, and Ada mRNAs were reduced in hepatocytes after the treatment with FFAs (Figure 1D). In vivo, ENTPD4‐6 mRNAs were reduced in high‐fat diet (HFD) mouse livers, while ENTPD1, ENTPD2, ENTPD5, ENTPD7, and Ada mRNAs were reduced in db/db mouse livers (Figure 1E,F). Collectively, ENTPD5 was the only consistently dysregulated ATP‐metabolizing gene in vivo and in vitro. ENTPD5 protein was reduced in steatotic human livers (Figure 1G), obese mouse livers and cultured hepatocytes (Figure 1H–K). ENTPD5 protein was mainly localized on the membrane of mouse hepatocytes, while it was distributed in both cytoplasm and membrane of human hepatocytes (Figure 1G–J). To evaluate the roles of ENTPD5 in metabolism, hepatocyte‐specific AAV8 subtype for overexpressing or inhibiting it were constructed and validated (Figure S1A–D, Supporting Information). In hepatocytes, ENTPD5 overexpression or inhibition decreased or increased lipid deposition (Figure 1L,M) and suppressed or promoted gluconeogenesis (Figure 1N) with the consistent nuclear translocation of forkhead box protein O1 (FOXO1) (Figure S1E, Supporting Information). Clearly, ENTPD5 regulates hepatic glucose and lipid metabolism.

### 2.2 Hepatic Modulation of ENTPD5 on Glucose and Lipid Metabolism

**Figure 2:**
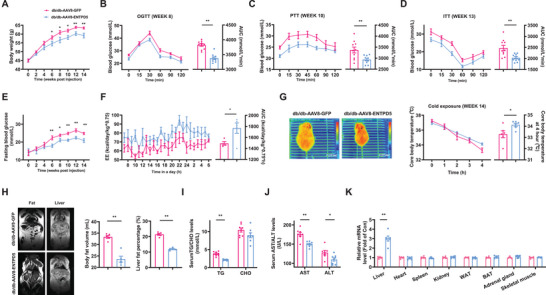
Hepatic ENTPD5 overexpression improved the dysregulation of glucose and lipid metabolism with activated BAT thermogenesis in db/db mice. A) Bodyweight curve of db/db mice post AAV8‐ENTPD5 injection (*N* = 10). B–D) AAV8‐ENTPD5 injection attenuated glucose intolerance (B), hepatic glucose production (C), and global insulin resistance (D) as evaluated by OGTT, PTT, and ITT in the indicated time point of mice (*N* = 10). E) The fasting blood glucose curve in db/db mice transduced with AAV8‐ENTPD5 (*N* = 10). F) AAV8‐ENTPD5 injection increased energy expenditure (EE) of mice as assayed in metabolic cages (*N* = 4–5). G) AAV8‐ENTPD5 injection activated BAT thermogenesis and increased core body temperature after cold exposure (*N* = 6). H) MRI analysis revealed that AAV8‐ENTPD5 injection reduced global and hepatic fat content of mice. Representative images were shown in left panel, and quantitative data shown in right panel (*N* = 5). I,J) The impact of AAV8‐ENTPD5 injection on serum TG/CHO (I), and AST/ ALT (J) levels (*N* = 8). K) AAV8‐ENTPD5 injection specifically increased ENTPD5 mRNA expression in the liver (*N* = 6). MRI: magnetic resonance imaging. Data of control mice were marked in red, and those of mice infected with AAV8‐ENTPD5 marked in blue. BAT, brown adipose tissues; TG, triglycerides; CHO, cholesterol; AST, alanine aminotransferase; ALT, aspartate aminotransferase. * *P* < 0.05, ** *P* < 0.01 versus control mice.

To determine the roles of hepatic ENTPD5 on glucose and lipid metabolism, it was overexpressed in db/db mouse livers via tail vein injection of AAV8‐ENTPD5. From sixth week post AAV8 injection, AAV8‐ENTPD5‐transduced mice exhibited lower bodyweight than control mice (**Figure** **2**A). Moreover, AAV8‐ENTPD5 injection improved glucose intolerance, hepatic glucose production, and insulin resistance in db/db mice (Figure 2B–D). AAV8‐ENTPD5 injection also ameliorated fasting hyperglycemia from sixth week (Figure 2E). AAV8‐ENTPD5 injection increased global energy expenditure (EE) (Figure 2F) with little effect on food intake, water drinking, respiratory quotient (RQ), and activity (Figure S2A–D, Supporting Information) as assayed in metabolic cages. AAV8‐ENTPD5 injection activated BAT thermogenesis and maintained core body temperature after cold exposure (Figure 2G), reduced global and hepatic fat content (Figure 2H), and decreased serum triglycerides (TG) but not cholesterol (CHO) levels (Figure 2I). AAV8‐ENTPD5 injection decreased serum alanine aminotransferase (ALT) and aspartate aminotransferase (AST) activities (Figure 2J). AAV8‐ENTPD5 injection specifically increased hepatic ENTPD5 mRNA expression (Figure 2K). Consistent with the amelioration of obesity, hepatic ENTPD5 overexpression increased lipolytic gene expressions in white adipose tissue (WAT) of obese mice (Figure S2E, Supporting Information).

**Figure 3:**
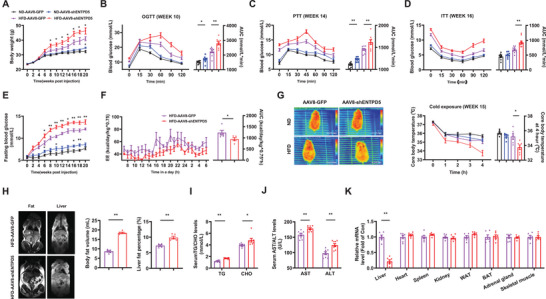
Hepatic ENTPD5 inhibition aggravated HFD‐induced glucose and lipid metabolic disorders, and obesity with reduced BAT thermogenesis in mice. A) Bodyweight curve of mice infected with AAV8‐shENTPD5 on ND or HFD feeding condition, AAV8‐GFP as control (*N* = 8–10). Mice were treated with AAV8‐GFP or AAV8‐ENTPD5, and then subjected to ND or HFD feeding, respectively. B–D) Impact of AAV8‐shENTPD5 injection on glucose tolerance (B), hepatic glucose production (C), and overall insulin sensitivity (D) in mice subjected to a normal diet (ND) or high‐fat diet (HFD) (*N* = 8–10). E) The fasting blood glucose curve in mice post AAV8‐shENTPD5 injection (*N* = 8–10). F) The EE curve and AUC data obtained in metabolic cage analyses of HFD mice at 15 weeks post AAV8‐shENTPD5 injection (*N* = 5). G) AAV8‐shENTPD5 injection reduced BAT thermogenesis and decreased core body temperature after cold exposure at 16 weeks post AAV8‐shENTPD5 injection (*N* = 6). H) MRI analysis revealed that AAV8‐shENTPD5 injection increased global and hepatic fat content. Representative images were shown in the left panel, and quantitative data shown in the right panel (*N* = 5). I,J) Impact of AAV8‐shENTPD5 injection on serum TG/CHO (I), and serum AST/ALT (J) levels in mice (*N* = 8). K) AAV8‐shENTPD5 injection specifically reduced ENTPD5 mRNA levels in the livers of mice (*N* = 6). Data of HFD‐AAV8‐GFP mice were marked in purple, and those of HFD‐AAV8‐shENTPD5 marked in red. * *P* < 0.05, ** *P* < 0.01 versus control mice.

To further validate the metabolic functions of hepatic ENTPD5, its expression was suppressed by tail vein injection of AAV8‐shENTPD5. Subsequently, mice were fed either a normal diet (ND) or an HFD. Under ND feeding, the bodyweight of mice exhibited difference at 20th week post AAV8‐shENTPD5 injection. Under HFD feeding, AAV8‐shENTPD5‐injected mice had higher bodyweight than control mice from week 8 post injection (**Figure** **3**A). AAV8‐shENTPD5‐injected mice showed the deteriorated glucose tolerance and hepatic glucose production compared with control under both ND and HFD feedings (Figure 3B,C). AAV8‐shENTPD5‐injected mice exhibited increased global insulin resistance than control after HFD feeding (Figure 3D). AAV8‐shENTPD5 mice showed elevated fasting blood glucose starting at week 6 after HFD feeding, but only transiently increased fasting blood glucose under ND feeding, prompting a focus on HFD feeding condition for subsequent studies (Figure 3E). These mice exhibited reduced EE without changes in food/water intake, activity, and RQ, and impaired BAT thermogenesis with reduced core body temperature during cold exposure (Figure 3F,G and Figure S2F–I, Supporting Information). AAV8‐shENTPD5 injection also increased global and hepatic fat content (Figure 3H), and elevated serum TG and CHO levels, as well as AST and ALT activities (Figure 3I,J). AAV8‐shENTPD5 injection reduced ENTPD5 mRNA in the liver but not other tissues (Figure 3K). Silencing of hepatic ENTPD5 reduced lipolytic gene expressions in WAT of HFD mice (Figure S2J, Supporting Information).

**Figure 4:**
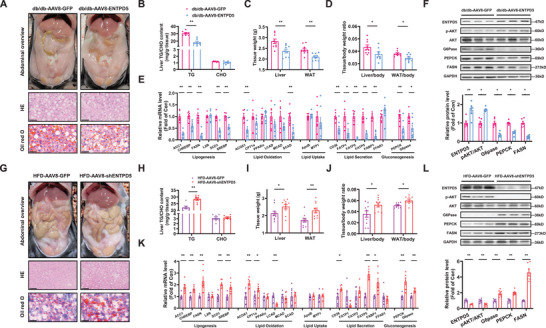
Modulation of ENTPD5 on lipid deposition and the expressions of metabolic genes in the livers of mice. A,B) Hepatic ENTPD5 overexpression attenuated lipid deposition in the livers of db/db mice as evidenced by morphological observation, H.E staining, Oil Red O staining (A), and lipid quantification (B) (*N* = 7). Scale bar: 50 µm. C,D) Hepatic ENTPD5 overexpression reduced the weight of liver and WAT (C), and the ratios to bodyweight (D) (*N* = 9). E,F) Hepatic ENTPD5 overexpression on the mRNA (E) and protein (F) levels of lipid metabolic and gluconeogenic genes in the livers of db/db mice (*N* = 6). G,H) Hepatic ENTPD5 silencing exaggerated HFD‐induced lipid deposition in the livers of mice as evidenced by morphological observation, H.E staining, Oil Red O staining (G), and lipid quantification (H) (*N* = 7). Scale bar: 50 µm. I,J) Hepatic ENTPD5 silencing increased the weight of liver and WAT (I), and the ratios to bodyweight (J) of HFD mice (*N* = 10). K,L) Hepatic ENTPD5 silencing on the mRNA (K) and protein (L) levels of lipid metabolic and gluconeogenic genes in the livers of HFD mice (*N* = 6–7). * *P* < 0.05, ** *P* < 0.01 versus control mice.

Combinational analysis revealed that ENTPD5 overexpression reduced TG but not CHO content in db/db mouse livers, and decreased the weight of liver and WAT, and their ratios to bodyweight (**Figure** **4**A–D). ENTPD5 overexpression led to significant downregulation in mRNA expression of key regulatory genes involved in lipid synthesis and efflux, with concomitant suppression of critical genes governing hepatic gluconeogenesis in db/db mouse livers (Figure 4E). Consistently, ENTPD5 overexpression activated Akt, and reduced key gluconeogenic and lipogenic proteins in db/db mouse livers (Figure 4F). Conversely, hepatic ENTPD5 silencing promoted TG deposition in mouse livers, and increased liver and WAT weight (Figure 4G–J) with primarily characterized by elevated transcriptional levels of genes associated with lipid synthesis and gluconeogenesis in HFD mouse livers (Figure 4K). Consistently, ENTPD5 silencing inactivated Akt, and increased key gluconeogenic and lipogenic proteins in mouse livers (Figure 4L)

### 2.3 Hepatic ENTPD5 Repressed Adrenomedullin Expression and Secretion

**Figure 5:**
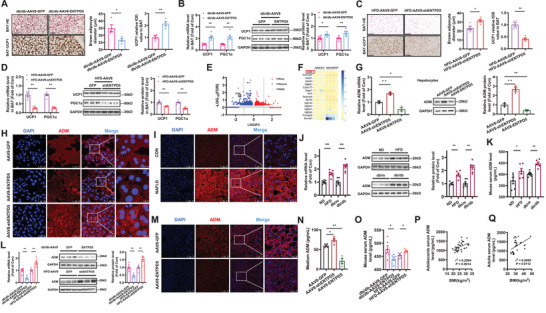
Hepatic ENTPD5 influenced BAT thermogenesis with altered ADM expression and secretion. A,B) Hepatic ENTPD5 overexpression increased the expressions of key thermogenic genes with decreased brown adipocyte size in BAT of db/db as indicated by H.E staining and immunohistochemical staining (A), as well as the mRNA and protein expressions of key thermogenic genes (B). Bar graphs represent the statistical analysis of brown adipocyte diameters and UCP1 grayscale values from immunohistochemical staining (A, *N* = 4), and the statistical analysis of densitometric values from WB (B, *N* = 6). Scale bar: 100 µm. C,D) Hepatic ENTPD5 silencing increased brown adipocyte size, bar graphs represent the statistical analysis of brown adipocyte diameters and UCP1 grayscale values from immunohistochemical staining (C, *N* = 4), while decreased the mRNA and protein expression levels of key thermogenic genes (D, *N* = 6) in BAT of HFD mice, bar graphs represent the statistical analysis of densitometric values from WB. Scale bar: 100 µm. E) The volcano plot of RNA‐sequencing analyses of different expression genes in mouse primary hepatocytes infected with AAV8‐GFP or AAV8‐ENTPD5 for 36 h (*N* = 4). F) Heat map of hepatocyte‐secreted protein genes affected by ENTPD5 overexpression in mouse primary hepatocytes. The color of the map represented the relative expression fold changes (*N* = 4). G) Change in the mRNA and protein levels of ADM in mouse hepatocytes with ENTPD5 overexpression or silencing, respectively (*N* = 3). H) Confocal imaging analysis of ADM protein in mouse primary hepatocytes after ENTPD5 overexpression or silencing. Scale bar: 50 µm, 10 µm. I) Confocal imaging staining of ADM protein in liver tissue specimens from patients with NAFLD. Scale bar, 75 µm, 25 µm. J) The mRNA and protein expressions of ADM were increased in the livers of obese mice (*N* = 6–7). K) Serum ADM level was increased in obese mice (*N* = 6). L) Hepatic overexpression or silencing of ENTPD5 on the mRNA and protein levels of ADM in obese mouse livers (*N* = 6). M) Confocal imaging analysis of ADM protein in db/db mouse livers after ENTPD5 overexpression. Scale bar: 75 and 25 µm. N) Impact of ENTPD5 overexpression or silencing on ADM level in the medium of cultured primary hepatocytes (*N* = 4). O) Serum ADM level in obese mice with hepatic ENTPD5 overexpression or silencing (*N* = 6). P) The correlation analysis between serum ADM level and BMI in overweighted or obese adolescent (*N* = 38). Q) The correlation analysis between serum ADM level and BMI in overweighted or obese adults (*N* = 30). * *P* < 0.05, ** *P* < 0.01 versus control mouse or control cell groups or control subjects.

Variable assays revealed that hepatic ENTPD5 overexpression increased the mRNA and protein levels of (UCP1) and peroxisome proliferator‐activated receptor gamma coactivator α (PGC1α), two pivotal thermogenic genes, in BAT of db/db mice with decreased brown adipocyte size (**Figure** **5**A,B), while hepatic ENTPD5 silencing exerted opposite effects (Figure 5C,D). RNA sequencing was then conducted to investigate the potential mechanism by which hepatic ENTPD5 regulates the UCP1 expression in BAT. Analysis revealed a total of 292 genes were regulated in hepatocytes overexpressing ENTPD5 (Figure 5E and Table S5, Supporting Information, and raw sequencing data are shown in Table S6, Supporting Information). Particularly, 16 genes that coded secretory proteins were affected by ENTPD5 overexpression in hepatocytes (Figure 5F and Table S5, Supporting Information). Furthermore, the changes in these 16 genes in db/db and HFD mouse livers after ENTPD5 overexpression or silencing were assayed. Consequently, adrenomedullin (ADM) was the only gene that was consistently regulated by ENTPD5 in mouse livers and hepatocytes (Figure 5F and Figure S3A,B, Supporting Information). ENTPD5 overexpression or silencing decreased or increased the mRNA and protein levels of ADM in hepatocytes (Figure 5G,H), confirming the RNA sequencing results. ADM mRNA and protein expression was increased in steatotic human and mouse livers (Figure 5I,J). Serum ADM levels were increased in obese mice (Figure 5K). Hepatic ENTPD5 overexpression or silencing decreased or increased hepatic expressions of ADM (Figure 5L,M and Figure S3C, Supporting Information). Importantly, modulation of ENTPD5 expression had little effect on the mRNA expressions of other ENTPD family members in hepatocytes and mouse livers (Figure S3D–F, Supporting Information). ENTPD5 overexpression or silencing decreased or increased ADM protein level in medium of cultured hepatocytes, and serum of obese mice (Figure 5N,O). Notably, in overweighted or obese adolescents and adults, serum ADM level was correlated with BMI (Figure 5P,Q), and diminished the positive correlation between serum ADM level and BMI in overweighted or obese adolescents (Figure S3G, Supporting Information). Injection of AAV‐ENTPD5 or AAV‐shENTPD5 had little effect on the level of ENTPD5 in other organs such as the heart, muscle, and adipose tissues beyond liver of obese mice (Figure S3H,I, Supporting Information).

**Figure 6:**
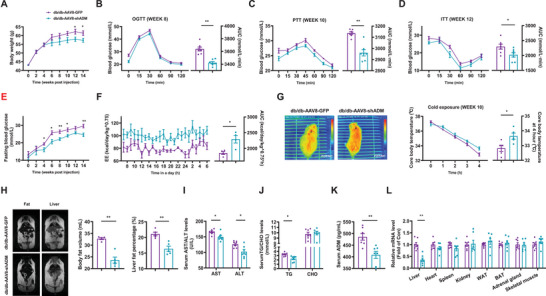
Hepatic ADM knockdown ameliorated glucose/lipid metabolic disorders and obesity with enhanced BAT thermogenesis in db/db mice. A) Bodyweight curve of db/db mice infected with AAV8‐shADM or AAV8‐GFP (*N* = 7). B–D) Injection of AAV8‐shADM improved glucose intolerance, gluconeogenesis, and insulin resistance of mice as assayed by OGTT (B), PTT (C), and ITT (D) (*N* = 7). E) Fasting blood glucose curve of db/db mice with AAV8‐shADM injection (*N* = 7). F) The EE curve and AUC data in metabolic cage analyses of db/db mice after AAV8‐shADM injection (*N* = 4). G) AAV8‐shADM injection activated BAT thermogenesis and increased key body temperature after cold exposure (*N* = 5). H) MRI assay indicated that AAV8‐shADM injection reduced global and hepatic content (*N* = 5). I,J) Change in serum AST/ALT (I) and TG/CHO (J) levels of db/db mice after AAV8‐shADM injection (*N* = 7). K) Serum ADM level of db/db mice after AAV8‐shADM injection (*N* = 7). L) Tissue expression profile of ADM mRNA in db/db mice after AAV8‐shADM injection (*N* = 7). *P < 0.05, ** *P* < 0.01 versus control mouse.

To determine the role of hepatic ADM in regulating BAT thermogenesis, liver‐specific AAV8‐shADM was constructed and validated (Figure S4A,B, Supporting Information). AAV8‐shADM administration reduced body weight and improved glucose intolerance, hepatic glucose production, insulin resistance, and fasting hyperglycemia in db/db mice. (**Figure** **6**A–E). AAV8‐shADM injection increased global EE and RQ (Figure 6F) without affecting food intake, water drinking, and activity (Figure S4C–F, Supporting Information). AAV8‐shADM injection increased BAT thermogenesis and core body temperature after cold exposure (Figure 6G). AAV8‐shADM injection reduced global and hepatic fat content (Figure 6H), and decreased AST and ALT activities, and TG and ADM levels in serum (Figure 6I–K). AAV8‐shADM injection specifically reduced ADM mRNA in the liver (Figure 6L).

**Figure 7:**
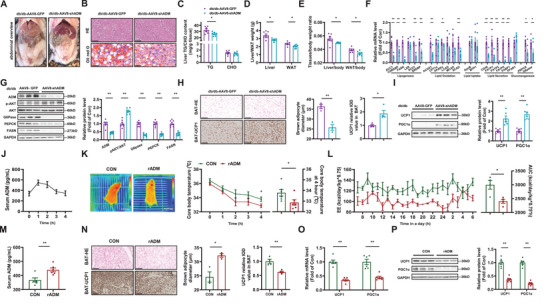
Thermogenic gene expression in BAT was activated by hepatic ADM knockdown but repressed by treatment with rADM in mice. A–C) Hepatic ADM inhibition reduced lipid deposition in the livers of db/db mice as evidenced by morphological observation (A), H.E staining (B, top), Oil Red O staining (B, bottom), and lipid quantification (C) (*N* = 6). Scale bar: 100 µm. D,E) Hepatic ADM inhibition reduced the weight of liver and WAT, and their ratios to bodyweight of db/db mice (*N* = 7). F,G) Hepatic ADM inhibition decreased the mRNA and protein levels of lipid metabolic and gluconeogenic genes in db/db mouse livers (*N* = 6–7). H,I) Hepatic ADM inhibition upregulated the expressions of key thermogenic genes with decreased brown adipocyte size in BAT of db/db as indicated by morphology, bar graphs represent the statistical analysis of adipocyte diameters from H.E staining (H‐left, *N* = 3), grayscale values statistics of UCP1 from immunohistochemical images (H‐right, *N* = 4) and key thermogenic protein levels (I, *N* = 6), bar graphs illustrate densitometric values from WB. Scale bar: 100 µm. J) Dynamic serum ADM in C57BL/6 mice after tail vein injection of rADM (5 nmol kg^−1^, *N* = 5). K) Treatment with rADM for 5 days reduced BAT thermogenesis and core body temperature after cold exposure in C57BL/6 mice (*N* = 6). L) Treatment with rADM for 1 week decreased EE of mice as assayed in metabolic cages (*N* = 4). M) Serum ADM in C57BL/6 mice after scarification (*N* = 6). N–P) Treatment with rADM inhibited the expressions of key thermogenic genes in BAT of wild type C57BL/6 mice in morphology, bar graphs represent the statistical analysis of adipocyte diameters and grayscale values from H.E staining (N‐left, *N* = 3) and immunohistochemical staining (N‐right, *N* = 4), as well as the mRNA (O, *N* = 6) and protein levels of key thermogenic genes (P, *N* = 6). rADM: recombinant ADM protein. * *P* < 0.05, ** *P* < 0.01 versus control mouse.

Hepatic ADM silencing ameliorated lipid deposition and TG content in db/db mouse livers, reduced the weight of liver and WAT, and their ratios to bodyweight (**Figure** **7**A–E). ADM silencing reduced the mRNA and protein levels of lipid metabolic and gluconeogenic genes with the activation of Akt in mouse livers (Figure 7F,G). Hepatic ADM silencing increased UCP1 and PGC1α expressions in BAT of db/db mice with decreased brown adipocyte sizes (Figure 7H,I). Hepatic ADM silencing increased lipolytic gene expressions in WAT of db/db mice (Figure S4G, Supporting Information). Hepatic silencing of ADM had little effects on the mRNA levels of ADM receptors in liver, BAT and WAT of obese mice (Figure S4H–J, Supporting Information). We also observed that overexpression of ENTPD5 in the liver significantly enhanced the browning of WAT, whereas knockdown of hepatic ENTPD5 resulted in a notable reduction in the expression of thermogenic genes in mouse WAT (Figure S4K,L, Supporting Information). To further confirm the inhibitory role of ADM on BAT thermogenic function, wild type C57BL/6 mice were treated with recombinant ADM protein (rADM, 5 nmol kg^−1^) via the tail vein injection.^[^ ^31^ ^]^ Dynamical change in serum ADM level after rADM injection was shown in Figure 7J. rADM administration for 5–7 days inhibited BAT thermogenesis and reduced core body temperature after cold exposure, and decreased EE and RQ without affecting food intake, water drinking, activity, bodyweight, and fasting blood glucose levels of mice (Figure 7K,L and Figure S5A–E, Supporting Information). rADM administration increased serum ADM level by about 30%, and reduced UCP1 and PGC1α expressions in BAT of mice (Figure 7M–P).

### 2.4 ENTPD5 Modulated Extracellular ADP/ATP Ratio to Repress ADM Expression in Hepatocytes

**Figure 8:**
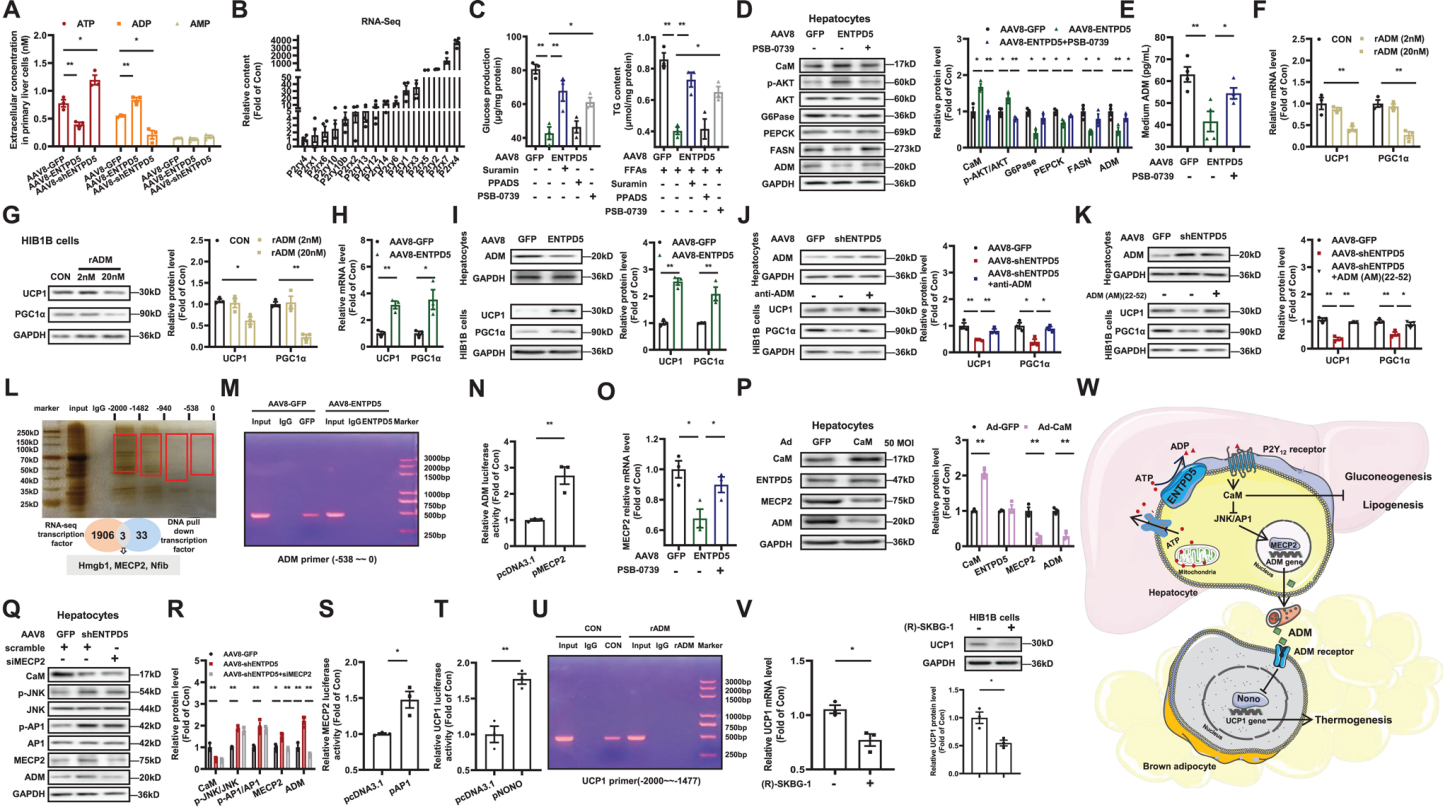
ENTPD5 activated ADP‐P2Y_12_ pathway to repress ADM expression, leading to the activation of BAT thermogenesis via NONO. A) Determination of extracellular ATP/ADP/AMP (eATP/eADP/eAMP) concentrations in mouse primary hepatocytes after infection with AAV8‐GFP, AAV8‐ENTPD5, or AAV8‐shENTPD5 for 36 h, respectively (*N* = 3). B) Relative P2 receptor subtype expression identified by RNA‐sequencing analyses in mouse primary hepatocytes (*N* = 4). C) ENTPD5‐induced suppression of gluconeogenesis and TG deposition was reversed by the treatment with suramin (50 µm), PPADS (50 µm), and P2Y_12_ inhibitor PSB‐0739 (20 µm) in hepatocytes (*N* = 3). D) Treatment with P2Y_12_ inhibitor reversed the regulatory effects of ENTPD5 on ADM and metabolic proteins in hepatocytes (*N* = 3). E) ENTPD5‐induced reduction in ADM secretion was reversed by P2Y_12_ inhibitor in hepatocytes (*N* = 4). F,G) Treatment with rADM reduced the mRNA(F) and protein levels(G)of UCP1 and PGC1α in HIB1B cells (*N* = 3). Cells were treated with 2 and 20 nm rADM for 36 h. H,I) In Hepatocte‐HIB1B cell coculture system, ENTPD5 overexpression in hepatocytes activated UCP1 and PGC1α mRNA (H) and protein (I) expressions in HIB1B cells (*N* = 3). J,K) In Hepatocte‐HIB1B cell coculture system, ENTPD5 silencing in hepatocytes reduced UCP1 and PGC1α expressions in HIB1B cells and were reversed by the incubation with anti‐ADM antibodies (J) or ADM receptor antagonist ADM (AM) 22–52 (K) (*N* = 3). L) Representative silver‐stained gel image of DNA pull‐down products in mouse primary hepatocytes. The protein bands pulldown using various mouse ADM gene promoter fragments in the red circles were cut and subjected to mass spectrometry analysis. 36 potential nuclear proteins were identified, while RNA sequencing revealed a total of 1909 transcribed transcription factor genes in hepatocytes. M) ChIP assay revealed that MECP2 bound to −532 to 0 region of mouse ADM gene promoter. N) Dual‐luciferase reporter assays indicated that MECP2 activated the promoter activity of mouse ADM gene in HEK293T cells (*N* = 3). O) ENTPD5 overexpression reduced the mRNA level of MECP2 in mouse primary hepatocytes, reversed by P2Y_12_ inhibitor (*N* = 3). P) The impact of CaM overexpression on the protein levels of ENTPD5, MECP2, and ADM in mouse primary hepatocytes (*N* = 3). Hepatocytes were infected with Ad‐CaM or Ad‐GFP (50 MOI) for 24 h, respectively. Q,R) The effect of ENTPD5 knockdown followed by MECP2 inhibition on the expression of JNK downstream proteins and ADM in primary hepatocytes (Q), bar graphs depict the statistical analysis of densitometric values derived from WB assays (R) (*N* = 3). S) AP1 overexpression activated mouse MECP2 gene promoter activity in HEK293T cells (*N* = 3). T) NONO overexpression activated the mouse UCP1 gene promoter activity in HEK293T cells (*N* = 3). U) ChIP assay revealed that NONO bound to −2000 to 1471 region of mouse UCP1 gene promoter, but the binding was inhibited by rADM treatment. V) Treatment with NONO inhibitor (R)‐SKBG‐1 (5 µm) for 24 h reduced the mRNA and protein expressions of UCP1 in HIB1B cells, bar graphs depict the statistical analysis of densitometric values derived from WB assays (*N* = 3). * *P* < 0.05, ** *P* < 0.01 versus control cells or between two indicated groups. W) Proposed mode for ENTPD5's regulatory mechanism(s) in hepatic glucose/lipid metabolism and BAT thermogenesis. Hepatocyte‐released ATP (eATP) is hydrolyzed by ENTPD5 on hepatocyte membrane to eADP, which acts on P2Y_12_ to activate CaM. Activation of CaM on one hand represses gluconeogenesis and lipogenesis. On the other hand, activated CaM reduces the expression and secretion of ADM in hepatocytes via the inhibition of JNK‐AP1‐MECP2 pathway. A decrease in circulating ADM activates NONO to induce key thermogenic gene expression and promote thermogenesis in BAT. Under overnutrition condition, inhibition of hepatic ENTPD5 expression will promote gluconeogenesis, lipogenesis and inhibit BAT thermogenesis to cause obesity. ADM: adrenomedullin; ADP: adenosine diphosphate; AP1: activated protein 1; ATP: adenosine triphosphate; CaM: calcium‐calmodulin; ENPTD5: ectonucleoside triphosphate diphosphohydrolase 5; JNK: c‐Jun N‐terminal kinase; MCEP2: methyl CpG binding protein 2; NONO: non‐POU domain‐containing octamer‐binding protein; P2Y_12_ receptors: purinergic receptor P2Y_12_; UCP1: uncoupling protein 1.

Overexpression of ENTPD5 reduced eATP level but increased eADP level without affecting eAMP level, while silencing of ENTPD5 exerted the opposite effects on eATP and eADP in cultured hepatocytes (**Figure** **8**A). Overexpression or inhibition of ENTPD5 had little effect on intracellular ATP content in hepatocytes and mouse livers (Figure S5F,G, Supporting Information). Purinergic receptor P2 receptors consist of P2X subtype, which contains seven members, and P2Y subtype, which contains eight members. Generally, P2X receptors are activated by ATP, while P2Y receptors are mainly activated by ADP.^[^ ^32^ ^]^ RNA sequencing revealed that P2 receptor subtypes were ubiquitously expressed in hepatocytes (Figure 8B and Table S6, Supporting Information). Treatment with suramin, inhibitor of both P2X and P2Y receptor subtypes, but not PPADS, inhibitor of P2X receptor subtype, reversed ENTPD5‐mediated inhibition of gluconeogenesis and lipid accumulation in hepatocytes (Figure 8C), suggesting that an increase in eADP predominantly activated P2Y subtypes to exert biological functions. The binding affinities of ADP to P2Y_12_ (Kd = 0.06 µm) and P2Y_13_ (Kd = 0.011 µm) are significantly higher than other P2Y members.^[^ ^29^, ^33^ ^]^ Because P2Y_12_ has higher expression level than P2Y_13_ in hepatocytes (Figure 8B), and there is no specific P2Y_13_ inhibitor currently available, whether ENTPD5 activates it to regulate glucose and lipid metabolism, and ADM expression was determined. ENTPD5's repression on gluconeogenesis and lipid deposition was significantly interrupted by P2Y_12_ inhibitor PSB‐0739 (Figure 8C). Moreover, ENTPD5's effects on phosphorylation of Akt, expression and secretion of ADM, and expressions of key metabolic genes were also reversed by P2Y_12_ inhibitor in hepatocytes (Figure 8D,E). Treatment with rADM reduced UCP1 and PGC1α expressions in brown adipocyte cell line HIB1B cells (Figure 8F,G). In hepatocyte‐HIB1B coculture system, hepatic ENTPD5 overexpression repressed ADM expression in hepatocytes, and upregulated UCP1 and PGC1α expressions in HIB1B cells (Figure 8H,I). Conversely, ENTPD5 silencing elevated ADM expression in hepatocytes, and decreased UCP1 and PGC1α expressions in HIB1B cells (Figure 8J), was blocked by treatment with anti‐ADM antibodies or ADM receptor antagonist ADM(AM)22‐52 (Figure 8J,K). In the co‐culture system, hepatocyte ENTPD5 knockdown significantly elevated ADM levels in the medium, whereas single or dual knockdown of ADM and ENTPD5 markedly reduced ADM levels compared to controls. Although ADM levels trended lower in the double‐knockdown group, no statistical significance was observed between single‐ and dual‐knockdown conditions (Figure S5H, Supporting Information). ENTPD5 silencing upregulated hepatic ADM expression and suppressed thermogenic gene expression in HIB1B cells, which was rescued by ADM inhibition. However, dual knockdown of hepatic ADM and ENTPD5 failed to further attenuate brown adipose thermogenic protein expression, suggesting a dominant regulatory role of ADM in this pathway (Figure S5I,J, Supporting Information). Overall, hepatic ENTPD5 inhibits ADM expression and secretion to promote UCP1 and PGC1α expressions in BAT.

DNA‐pulldown using four probes spanning the mouse ADM promoter (−2000 to 0 bp) identified proteins binding to this region in hepatocytes. Consequently, mass spectrometry (MS) analysis identified 36 nuclear proteins in four indicated pulldown protein band areas in red circles (Figure 8L and Table S7, Supporting Information). Moreover, RNA sequencing identified the signals of 1909 transcription factors in hepatocytes (Table S6, Supporting Information). Simple combination analysis of RNA sequencing and DNA‐pulldown–MS results yielded three universal transcription factors or cofactors: methyl CpG binding protein 2 (MCEP2), high mobility group box 1 (Hmgb1) and nuclear factor I/B (Nfib) (Figure 8L). MCEP2 and Hmgb1 expressions were increased in HFD mouse livers, while only MCEP2 was increased db/db mouse livers (Figure S6A, Supporting Information). In mouse livers with ENTPD5 silencing, MCEP2 and Nfib expressions were increased, while only MCEP2 was decreased in mouse livers with ENTPD5 overexpression (Figure S6B, Supporting Information). In hepatocytes, ENTPD5 overexpression reduced MCEP2 and Hmgb1 mRNA levels, while ENTPD5 silencing only increased MCEP2 mRNA level (Figure S6C, Supporting Information). Moreover, Nfib overexpression failed to activate mouse ADM gene promoter activity (Figure S6D, Supporting Information). So, whether ENTPD5 modulated ADM transcription via MECP2 was investigated. Chromatin immunoprecipitation (ChIP) experiments revealed that MCEP2 bound with −538 to 0 fragment (Figure 8M) but not −2000 to −538 region of mouse ADM gene promoter (Figure S6E, Supporting Information). MCEP2 overexpression activated mouse ADM gene promoter activity (Figure 8N). In hepatocytes, ENTPD5 overexpression decreased MECP2 mRNA level, which was blocked by P2Y_12_ inhibition (Figure 8O). ENTPD5 overexpression reduced MCEP2 protein level in mouse livers and hepatocytes, while ENTPD5 silencing increased its expression in mouse livers (Figure S6F,G, Supporting Information). MCEP2 overexpression increased ADM expressions in hepatocytes (Figure S6H, Supporting Information). In our previous studies, we had demonstrated that secreted ATP activated CaM to modulate the transcriptions of genes in various cell types^[^ ^5^, ^6^ ^]^ ENTPD5 overexpression increased CaM protein level but was reversed by P2Y_12_ inhibition in hepatocytes (Figure 8D). CaM overexpression reduced MCEP2 and ADM protein levels without affecting ENTPD5 protein in hepatocytes (Figure 8P). We had recently demonstrated that PANX‐1–mediated ATP release activated CaM to interact with and inhibit c‐Jun N‐terminal kinase (JNK) activity, repressing the activity of transcription factor activated protein 1 (AP1).^[^ ^7^ ^]^ Bioinformatic prediction identified several potential binding sites for AP1 in mouse and human MCEP2 genes (data not shown). In hepatocytes, ENTPD5 silencing reduced CaM protein, increased JNK and AP1 phosphorylations, and elevated MCEP2 and ADM expressions (Figure 8Q,R). Notable, MCEP2 silencing reversed ENTPD5's inhibition on ADM expression without affecting the expressions of CaM, and phosphorylated JNK and AP1 (Figure 8Q,R). AP1 overexpression activated mouse MCEP2 gene promoter activity (Figure 8S), and induced its expressions in hepatocytes (Figure S6I, Supporting Information). Clearly, ENTPD5‐mediated ATP hydrolysis activates P2Y_12_ to activate CaM, which suppresses JNK and AP1 activity to reduce MECP2 and ADM transcriptions in hepatocytes. Then, the mechanism of ADM‐induced UCP1 repression in BAT cells was probed. Four probes flanking −2000 to 0 bp of UCP1 gene promoter were designed to pulldown proteins that bound with it in HIB1B cells. The four regions in red circles were subjected to MS analysis (Figure S7A, Supporting Information, and MS pulldown data are shown in Table S8, Supporting Information). As a results, 35 transcription factors or cofactors or nuclear proteins including the KH domain proteins (Krr1), non‐POU domain‐containing octamer‐binding protein (NONO), proliferation‐associated nucleolar protein P120 (Nop2), quaking proteins (Qki), and cell division cycle 5 like protein (Cdc5I) were identified (Table S7, Supporting Information). To initially identify the potential transcription factors or cofactors that mediate ADM's regulatory effects on UCP1 expression, the mRNA expressions of Krr1, NONO, Nop2, Qki, and Cdc5I in BAT of mice with hepatic ENTPD5 overexpression or silencing, hepatic CaM silencing, and in HIB1B cells cocultured with AAV8‐ENTPD5–infected hepatocytes were determined. Consequently, NONO was the only gene that was consistently regulated in these experiments (Figure S7B,F, Supporting Information). Hepatic overexpression of ENTPD5 or silencing of ADM increased, while hepatic silencing of ENTPD5 decreased NONO expression in BAT of mice (Figure S7C,F, Supporting Information). In hepatocyte‐HIB1B cell coculture system, hepatic ENTPD5 overexpression upregulated NONO expression, but was blocked by P2Y_12_ inhibitor in HIB1B cells (Figure S7G,H, Supporting Information). Overexpression of NONO activated mouse UCP1 gene promoter activity (Figure 8T). In contrast, overexpression of Krr1 failed to affect the UCP1 gene promoter activity (Figure S7I, Supporting Information). ChIP analysis revealed that NONO bound with the −2000 to 1477 bp region, but not −1477 to 0 bp region of mouse UCP1 gene promoter (Figure 8U and Figure S7J, Supporting Information), and overexpression of NONO upregulated UCP1 mRNA and protein expressions in HIB1B cells (Figure S7K, Supporting Information). Importantly, rADM treatment reduced the binding of NONO with mouse UCP1 gene promoter (Figure 8U). Treatment with NONO inhibitor reduced UCP1 expressions in HIB1B cells (Figure 8V).

In a hepatocyte‐HIB1B coculture system, hepatic AP1 overexpression increased ADM expression and secretion, and inhibited NONO and UCP1 expressions in HIB1B cells, but this inhibitory effect was reversed by treatment with anti‐ADM antibodies or the ADM receptor antagonist (Figure S8A, Supporting Information). Moreover, treatment with anti‐ADM antibodies reduced ADM level in the medium, while treatment with ADM receptor antagonist failed to do so (Figure S8A, Supporting Information). Furthermore, in hepatocyte‐HIB1B coculture system, hepatic ENTPD5 overexpression inactivated AP1, and repressed MCEP2 and ADM expressions in hepatocytes, decreased ADM level in the medium, and upregulated NONO and UCP1 expressions in HIB1B cells, but these effects were reversed by hepatic AP1 overexpression (Figure S8B, Supporting Information). Moreover, the significantly increased gluconeogenesis and lipid deposition induced by ENTPD5 silencing in hepatocytes were not affected by co‐incubation with anti‐ADM antibodies or an ADM receptor antagonist. This suggests that liver‐derived ADM does not locally regulate hepatocyte metabolism in an autocrine/paracrine manner (Figure S8C,D, Supporting Information). Knockdown of ENTPD5 in primary hepatocytes results in the suppression of thermogenic gene expression in the co‐cultured HIB1B cells, and this inhibitory effect was exacerbated by the application of a NONO inhibitor (Figure S8E, Supporting Information). Further studies demonstrated that hepatis overexpression of ENTPD5 leads to a reduction in the expression levels of MECP2 and ADM in primary hepatocytes. Concurrently, an increase in the protein expression of key thermogenic molecules in the co‐cultured HIB1B cells induced by ENTPD5 overexpression or ADM silencing in hepatocytes was reversed by the application of NONO inhibitor (Figure S8F,G, Supporting Information). These results elucidated the critical roles of hepatic ADM in modulating the functions of brown adipocytes.

## 3 Conclusion

The current study provided the first report revealing hepatic ENTPD5's essential role in regulating hepatic and global metabolism by eATP homeostasis. ENTPD5 not only hydrolyzes ATP but also interacts with fibroblast growth factor 23 and sodium‐dependent phosphate transporter 2a, participating in biological processes like bone metabolism.^[^ ^36^ ^]^ Our study demonstrated that inhibition of hepatic ENTPD5 played important roles in triggering the development and progression of obesity and metabolic disorders. Together with the previous findings,^[^ ^5^, ^9^, ^10^, ^11^, ^12^ ^]^ a series of our study has established the vital roles of ATP synthesis, secretion, and metabolism in regulating hepatic and global glucose and lipid metabolism.

It has been recognized that the liver can release various hepatokines to control the functions of distant organs.^[^ ^37^, ^38^, ^39^ ^]^ Our findings have revealed that ENTPD5‐mediated ATP hydrolysis in hepatocytes plays important roles in regulating thermogenesis in BAT via the repression of hepatic ADM expression and secretion through the purinergic receptor P2Y_12_ signaling pathways. Hepatic ENTPD5 activates ADP‐P2Y_12_‐CaM‐JNK‐AP1 signaling pathway to repress the transcription of MECP2 within hepatocytes, thereby inhibiting the expression and secretion of ADM. Previous studies have suggested that the JNK‐AP1 pathway plays important roles in obesity by influencing transcription or epigenetic pathways, promoting inflammation, affecting adipose growth and function, and regulating metabolic homeostasis and inflammatory cytokine expression, thereby impacting obesity‐related metabolic disturbances.^[^ ^40^, ^41^ ^]^ The AP‐1 signaling pathway plays an important role in hepatic steatosis^[^ ^42^ ^]^ and is involved in regulating adipose tissue function. Here we provided novel and convincing evidence that hepatic AP1 regulated the expression and secretion of ADM to modulate UCP1 expression and thermogenesis in BAT. A decrease in circulating ADM stimulates the expression of UCP1 and promotes the thermogenesis in BAT via the activation of transcription cofactor NONO. Clearly, ADM is a novel hepatokine that links hepatic metabolic deregulation to the impairment of BAT thermogenesis. Our findings strongly suggest that altered hepatic eATP metabolism plays important roles in triggering obesity. In other words, obesity may partly stem from the dysregulation of eATP metabolism and ADM secretion in hepatocytes.

ADM is a peptide expressed most abundantly in the adrenal medulla,^[^ ^43^ ^]^ and has vasodilatory effects.^[^ ^44^ ^]^ Adrenal medulla‐secreted ADM is involved in the pathogenesis of liver fibrosis, hepatitis, and cirrhosis by regulating hepatic perfusion, inhibiting hepatic stellate cell activation, and exerting anti‐inflammatory and antioxidant effects.^[^ ^45^ ^]^ Moreover, treatment with ADM mimicked the effect of obesity and induced endothelial and systemic insulin resistance in mice.^[^ ^46^ ^]^ Additionally, ADM is also expressed in adipose tissue along with its receptors,^[^ ^47^ ^]^ playing a role in the pathogenesis of obesity and related diseases.^[^ ^48^, ^49^, ^50^ ^]^ Here we observed ADM is expressed in hepatocytes, and hepatocyte‐released ADM negatively impacts BAT functions. Additionally, serum ADM levels are elevated in obese mice, adolescents, and adults. Importantly, there is a positive correlation between serum ADM levels and BMI in obese adolescents and adults. Based on the effects of hepatic overexpression of ENTPD5, or hepatic silencing of ADM on serum ADM level, we speculated that hepatic ADM secretion might contribute to about 20–30% of circulating ADM under obese condition. Moreover, our findings also revealed that chronic increase in circulating ADM level by 20–30% was sufficient to impair the thermogenesis in BAT. Given that ADM is widely involved in the regulation of cardiovascular functions and inflammatory process, increased ADM secretion from the liver may trigger other diseases such as vascular diseases and global inflammation beyond metabolic diseases.

Patients with Rett syndrome (RTT, OMIM #312 750) exhibits pathogenic variants of the MECP2 with loss of function mutations. These patients have metabolic dysfunctions in addition to the previously recognized neurodegenerative changes.^[^ ^51^, ^52^ ^]^ However, the mechanism of MECP2 in the regulation of glucose and lipid metabolism remains unclear in RTT patients. This study provided the direct metabolic roles of MECP2 by controlling the expression of ADM in the liver. Our research revealed that ADM is a novel hepatokine linking hepatic eATP metabolism to BAT thermogenesis via NONO. Current research on NONO is mainly focused on tumorigenesis.^[^ ^53^ ^]^ Our findings revealed that NONO plays important roles in regulating UCP1 expression and BAT thermogenesis. However, although MCEP2 and NONO bound with the ADM and UCP1 gene promoter, respectively, no corresponding binding site(s) was predicted by bioinformatic analysis. It is also possible that they may bind to the untraditional site(s) in the target gene promoters or function as transcription cofactors.

In summary, hepatic ENTPD5 plays important roles in maintaining glucose and lipid metabolism by hydrolyzing eATP to eADP, which activates P2Y_12_‐CaM pathways to regulate hepatic glucose and lipid metabolism. Particularly, hepatic ENTPD5 regulates ADP‐P2Y_12_‐CaM‐AP1‐MECP2 pathway to inhibit the expression and secretion of ADM, which modulates the thermogenesis of BAT. Under overnutrition condition, inhibition of hepatic ENTPD5 will trigger the excessive expression and secretion of ADM to inhibit BAT thermogenesis, causing obesity and metabolic dysfunctions (Figure 8W). The ENTPD5‐ADM cascade may mediate as a potential mechanistic link between metabolic disturbances and increased cardiovascular risk. Investigating this cascade could unveil novel therapeutic targets aimed at ameliorating cardiovascular complications in individuals with metabolic syndrome or type 2 diabetes. Targeting hepatic ENTPD5‐ADM pathway represents a novel strategy for combating against obesity and metabolic disorders.

## 4 Experimental Section

### Ethics Statement

The animals in this study were used in accordance with the Guide for Care and Use of Laboratory Animals of the National Institute of Health, and the protocols were approved by the Institutional Animal Care and Use Committee of Peking University Health Science Center. For the human specimens and serum samples used in this study, informed consent was obtained from all individuals and recorded in an electronic database. Research Board protocols were conducted under the guidelines set out by the Medical Ethical Committees of the Affiliated Beijing Chaoyang Hospital of Capital Medical University.

### RNA Extraction and RT‐qPCR

Total RNA was extracted from tissues or cells using TRIzol (TRANS, ER501‐01‐V2) according to the manufacturer's instructions. Reverse transcription was conducted using Stastagene Mx3000P real‐time quantitative PCR system (Agilent Technologies, 76 337 Waldbronn). Real‐Time PCR was performed using Stastagene Mx3000P real‐time quantitative PCR system (Agilent Technologies). Target gene mRNA levels were normalized to β‐actin. All primer pair sequences used in RT‐PCR are provided in Table S1 (Supporting Information).

### Western Blotting Assays

Tissues or cells were lysed in Roth Lysis buffer with proteinase inhibitors. 20–40 µg protein were separated by SDS‐PAGE, and then transferred to the NC (nitrocellulose filter, 66 485) membrane. The membrane was incubated at 4 °C with the following primary antibody overnight: anti‐ENTPD5 (proteintech, 26746‐1‐AP), anti‐pAkt (Cell Signaling Technology, 9271) (Cell Signaling Technology, 4060S), anti‐Akt (Cell Signaling Technology, 9272S), anti‐G6Pase (Santa Cruz, sc‐25840), anti‐PEPCK (Bioworld, BS6870), anti‐fatty acid synthase (Abcam, ab22759), anti‐UCP1 (Abclonal,A5857), anti‐PGC1α (Abclonal, A11971), anti‐ADM (Cloudclone, MAA220Hu22), anti‐CaM (Abclonal, A10769; Abcam, ab45689), anti‐JNK (Immunoway, YT2439), anti‐p‐JNK (Cell Signaling Technology, 9521), anti‐AP1 (Immunoway, YN3243), anti‐p‐AP1 (Abcam, ab273448), anti‐FOXO1 (Cell Signaling Technology, 2880) and anti‐GAPDH (Cell Signaling Technology, 5174). Then the primary antibody was washed with TBST and incubated with second antibody. Blots were visualized using enhanced chemiluminescence. Protein levels were quantified by densitometry using Image J software, and data were normalized to GAPDH protein expression. Antibodies used for western blotting are listed in Table S1 (Supporting Information).

### Clinical Samples

The clinical liver histopathology specimens used in this study were obtained from surgical resections of patients with non‐neoplastic liver diseases. All diagnoses of NAFLD were based on histopathological examinations. The human tissue samples used in this study was approved by the Research Ethics Committee of Peking University People's Hospital (Ethics Number 2020PHB337–01), and the clinical characteristics of these human subjects are detailed in Table S2 (Supporting Information). The plasma from healthy individuals and patients with diabetes were obtained with informed consent and were approved by the Ethics Committee of Peking University Third Hospital (Ethics Number 2021‐283‐07) and Affiliated Beijing Chaoyang Hospital of Capital Medical University (Ethics Number 2024‐KE‐958). The physiological and biological parameters are detailed in Tables S3 and S4 (Supporting Information). The study design and conduct complied with all relevant regulations regarding the use of human study participants and adhered to the criteria set by the Declaration of Helsinki.

### Animals

C57BL/6J, db/db mice with a BKS background were purchased from Vital River Company and used in this study. C57BL/6J mice were fed either a 45% HFD (Research Diets, D12492) or a normal diet (ND) for 20 weeks to induce diabetic and steatotic phenotypes. Male db/db mice were fed on ND for 14 weeks. All mice were male and 8–10 weeks old. Body weight and fasting blood glucose levels were assessed every 2 weeks. All animals were housed in a controlled environment with a 12‐h light, 12‐h dark cycle at a temperature ranging from 20 to 25 °C. Oral glucose tolerance test (OGTT), insulin tolerance test (ITT), and pyruvate tolerance test (PTT) were performed at different time points after injection to monitor the metabolic phenotype changes. The detailed protocols for OGTT, ITT, and PTT have been described in our previous studies.^[^ ^10^ ^]^ All protocols involving experimental animals were approved by the Institutional Animal Care and Use Committee of Peking University Health Science Center.

### AAV Virus Preparation and In Vivo Transduction

Liver‐specific adeno‐associated virus (AAV8) subtype was packaged by Yibaike Biotech Co., Ltd (Beijing, China). All constructs were confirmed by Sanger sequencing, and the final titer was greater than 2.0 × 10^13^ viral genomes per mL. A total of 5 × 10^11^ vg AAV8‐ENTPD5 or AAV8‐GFP were injected into tail veins of db/db mice. To knockdown ENTPD5 in the liver, 5 × 10^11^ vg AAV8‐shENTPD5 or AAV8‐GFP were injected into tail veins of mice fed on 45%HFD or ND for 20 weeks. To knockdown ADM in the liver, 5 × 10^11^vg AAV8‐shADM or AAV8‐GFP were injected into the tail veins of db/db mice.

### Cell Culture

Primary mouse hepatocytes were cultured and infected with adeno associated virus or adenovirus as previously detailed.^[^ ^11^ ^]^ The pre‐brown adipocyte cell line HIB1B was generously provided by associate professor Dandan Zhao from Diabetes Center of Beijing University of Chinese Medicine. HIB1B and 293T cells were cultured in DMEM medium (Gibco) supplemented with 10% FBS, 100 units per mL penicillin, and 100 units per mL streptomycin in an incubator at 37 °C with 5% CO_2_. After reaching confluence, cell differentiation was induced using high‐glucose DMEM containing 10% FBS, 20 nm insulin, 1 nm triiodothyronine (T3), 0.5 mm IBMX, and 1 µm dexamethasone for 2 days. Cells were re‐fed every other day with DMEM containing 10% FBS and the same concentrations of insulin and T3. The HIB1B brown adipocytes, after differentiation and maturation were co‐cultured with pre‐treated primary mouse hepatocytes (AAV8‐GFP, AAV8‐ENTPD5, AAV8‐shENTPD5, 36 h).

### H&E and Immunohistochemistry Staining

Liver samples were fixed in 4% paraformaldehyde and embedded with paraffin. Samples were sliced into 5–8 µm in thickness and then subjected to hematoxylin and eosin (H&E) staining. For immunohistochemistry, sections were dewaxed, rehydrated, and then incubated in EDTA antigen retrieval buffer (ZSGB‐BIO, ZLI‐9072) for 5 min at 100 °C. Slices were then incubated with 3% H_2_O_2_ for 10 min, washed three times with PBS containing 0.02% Triton X‐100 (Sigma‐Aldrich, T8787), followed by incubation with primary antibodies overnight at 4 °C. Horseradish peroxidase‐conjugated secondary antibody was incubated at 37 °C for 1 h. The color was developed by incubation with DAB kit (ZSGB‐BIO, ZLI‐9017). Sections were counterstained with hematoxylin (Baso, BA4041) and examined under the microscope (Olympus, CKX41SF). Primary antibodies used in the immunohistochemistry staining are listed in Table S1 (Supporting Information). Quantification for immunohistochemistry staining positive area was conducted in 5–8 random fields (10× magnification) per mouse using ImageJ software (version 1.8.0).

### Oil Red O Staining

Liver tissues were fixed, embedded with OCT compound, and sliced into 5–8 µm in thick sections. Oil Red O (Sigma‐Aldrich, O0625) powder was dissolved in isopropanol at a concentration of 0.7 g per 100 mL and then diluted with water at a volume ratio of 3:2 to prepare the Oil Red O solution. Pre‐warmed tissue sections were washed twice with PBS and stained with Oil Red O solution for 20 min, then were washed with 60% isopropanol three times for 10 s each, followed by counterstaining with hematoxylin (Baso, BA4041), and then were viewed under the microscope (Olympus). The Oil Red O staining positive area was quantified with 5–*8* random fields per mouse using ImageJ software (version 1.8.0).

### Cells Lipid Staining

Mouse hepatocytes were administrated with FFAs (0.2 mmol L^−1^ oleic acid and 0.4 mmol L^−1^ palmitic acid) in the presence of different treatments (AAV8‐GFP, AAV8‐ENTPD5, and AAV8‐shENTPD5), scramble (scrambled siRNA), and siMECP2 (MECP2 siRNA) for 24 h. After the treatment, cells were fixed by 4% paraformaldehyde and stained with Lipid TOX (Invitrogen, H34476). The nuclei were stained with DAPI, and then observed by a confocal laser scanning microscope (Olympus, CKX41SF).

### Hepatocyte Glucose Production Assay

Primary mouse hepatocytes were treated with AAV8‐GFP, AAV8‐ENTPD5, or AAV8‐shENTPD5 for 36 h then washed by PBS for three times. The glucose content was detected using the Glucose Assay Kit (Sigma‐Aldrich, GAGO‐20) according to the manufacturers’ instructions and normalized to the protein content in each sample (µg mg^−1^ protein^−1^).

### Cold Exposure Experiments

Mice were housed in an artificial climate chamber at 4 °C after their core body temperatures were measured using a rectal probe (TH212) at room temperature (25 °C). The core body temperatures were measured every hour. The infrared thermometer (Fotric) was used to take pictures of the mice after 4 h of exposure to 4 °C, as previously described.^[^ ^10^ ^]^

### Metabolic Cage Analyses

Each mouse was housed in a sealed metabolic cage for 24 h to monitor energy expenditure (EE), respiratory exchange rate (RQ), physical activities, and food and water intake. The environment was controlled at 22.5 ± 2.5 °C. EE was calculated using the comprehensive laboratory animal monitoring system formula: EE = (3.815 + [1.232 × RQ]) × oxygen consumption × 1.44. Animal activity was monitored by horizontal and vertical infrared sensor pairs arranged on the strips, as previously described.^[^ ^10^ ^]^

### Magnetic Resonance Imaging Analyses

Mice were placed horizontally in small‐animal magnetic resonance imaging (MRI) mount and continuously anesthetized with isoflurane. Fat distribution and images of the whole body and liver were scratched and quantitatively analyzed using a Siemens MRI system 3.0 T, Trio Tim scanner, and Siemens Syngo software, as previously described.^[^ ^10^ ^]^

### RNA‐Sequencing Analyses

Primary mouse hepatocytes were infected with AAV8‐GFP or AAV8‐ENTPD5 for 36 h and then collected for RNA sequencing, which was performed using Illumina by Mega Genomic (China). Different expression gene (DEG) analyses were performed using the DESeq, Genes with a fold change >2 or <1/2 and a false discovery rate <0.01 was considered to be significantly DEGs.

### Lipid Measurement

Triglyceride (TG) and CHO content in serum, cells, and livers were tested using TG and CHO determination kit according to the manufacturer's instructions (E1013 and E1015, Applygen). Hepatocytes were administrated with FFAs (0.2 mmol L^−1^ oleic acid and 0.4 mmol L^−1^ palmitic acid) in the presence of different treatments (AAV8‐GFP, AAV8‐ENTPD5, and AAV8‐shENTPD5) and/or PSB0739 (HY‐108660, 20 µmol L^−1^) for 36 h. After the treatment, cells were fixed by 4% paraformaldehyde and stained with Lipid TOX (Invitrogen, H34476). DAPI was stained for the nucleus, then observed by a confocal laser scanning microscope.

### Serum ADM Detection

Serum ADM levels were detected by ELISA kit (Dogesce DG30221M, KT0729‐A) in mouse and human as manufacturer's instruction.

### ATP, ADP, and AMP Detection by LC–MS

The levels of the three compounds (ATP, ADP, and AMP) were measured using a TSQ Quantiva interfaced with Ultimate 3000 Liquid Chromatography system (Thermo Scientific) based on previously published studies.^[^ ^30^ ^]^

### DNA Pull Down

The promoter sequence of mouse ADM was divided into four segments as follows: −2000 to −1482, −1501 to −920, −940 to −510, and −538 to 0. The promoter sequence of mouse UCP1 was divided into four segments as follows: −2000 to −1477, −1501 to −1104, −1126 to −494, and −514–0. Primers were constructed and amplified for each sequence, labeled with 5′‐biotin. DNA fragments were recovered by agarose gel electrophoresis, and their concentrations were measured. Primary mouse hepatocytes were treated with AAV8‐GFP or AAV8‐ENTPD5 for 48 h, and the HIB1B brown adipocytes were treated with 20 nm rADM. Nuclear and cytoplasmic fractions were extracted using nuclear/cytoplasmic/membrane preparation kit (HX1852, Huaxingbio). 200 µg of hepatocytes were incubated and rotated at room temperature for 1 h with 2 µg of biotinylated DNA probe in 150 µL of binding buffer. After this 1 h incubation, 20 µL of high‐capacity streptavidin magnetic beads (HY‐K0208, Med Chem Express [MCE], Shanghai, China) were added to the binding reaction and further incubated for 1 h at room temperature. The binding reaction was terminated by centrifugation at 5000 rpm for 1 min and washing the resins with 1 mL of washing buffer twice. After the complete removal of the washing buffer, 12 µL of a 2 × SDS‐PAGE sample buffer was added, and the mixture was heated at 95 °C for 10 min. The eluted protein was detected the corresponding protein by immunoblotting assays and mass spectrometry.

### Luciferase Reporter Assay

The mouse MECP2, ADM, NONO, or UCP1 gene promoter were cloned into the pGL3‐basic vector, respectively. The positive clones were selected and confirmed by DNA sequencing. The activation of firefly luciferase and ranilla luciferase were measured with the Dual‐Luciferase reporter assay kit (Promega, USA) at 36 h after transfection according to the manufacturer's instructions.

### ChIP

Primary mouse hepatocytes cells or the HIB1B brown adipocytes were washed three times with warm PBS and then fixed with 1% formalin for 10 min at 37 °C. The cells were washed and collected using cold PBS, the centrifuged at 3000 rpm for 10 min at 4 °C. The cells were dissolved in 300 µL lysis buffer and incubated on ice for 10 min. The lysate was centrifuged at 14 000 × *g* for 10 min. The lysate was pre‐cleared using the control agarose resin and salmon sperm DNA for 2 h, and centrifuged at 1000 rpm for 5 min. The flowthrough was collected. 2 µg antibody (anti‐MECP2/NONO, with normal IgG as control) were added to the lysate, and then the mixture was incubated with gentle end‐over‐end mixing or shaking overnight at 4 °C. Agarose resin and salmon sperm DNA were added to the lysate, followed by a 2 h incubation at 4 °C. The mixture was centrifuged at 1000 rpm for 5 min, and the flow‐through was discard. Then the samples were washed once with TSEI, TSEII, TSEIII, and TE in turn. 100 µL of elution buffer was added to the sample, and then it was centrifuged. The final sample was incubated at 65 °C for 6 h to isolate the protein and DNA. DNA was extracted using phenol, chloroform, and isoamyl alcohol. Agarose gel electrophoresis was performed after PCR. The primer sequences used in this research are provided in Table S1 (Supporting Information).

### Statistical Analysis

All experimental results were reported as Mean ± standard error (Mean ± SEM) and the data were analyzed using GraphPad Prism 8.0. If the data conformed to a normal distribution, a *t*‐test (two groups) or ANOVA (multiple groups) was used for analysis. If the data were not normally distributed, the Mann–Whitney *U* test (two groups) and the Kruskal–Wallis *H* rank‐sum test (multiple groups) were used for statistical analysis, *P*‐values <0.05 were used as the criterion for a significant difference.

## Conflict of Interest

The authors declare no conflict of interest.

## Author Contributions

R.M. and S.H. contributed equally to this work. R.M., S.H., R.X., and W.L. researched data and contributed to the discussion. R.M., J.L., and J.Y. wrote the manuscript. X.L. assisted in raising animals and samples preparation. J.L. and M.X. provided human liver and serum samples. R.M., H.Y., Y.C., J.L., and J.Y. designed the study and revised/edited manuscript. All corresponding authors are the guarantors of this work and take responsibility for the integrity of the data and the accuracy of the data analysis.

## Supporting information



Supporting Information

Supporting Information

Supporting Information

## Data Availability

All the datasets presented in the paper are available from the corresponding author upon reasonable request.
