# Supplementary material for: Hepatic ENTPD5 Is Critical for Maintaining Metabolic Homeostasis and Promoting Brown Adipose Tissue Thermogenesis
Source: Adv Sci (Weinh). 2025 Aug 11;12(40):e03603. doi: 10.1002/advs.202503603 (PMC12561356; doi:10.1002/advs.202503603)
Supplement: Supplementary file 1 — Supporting Information [file ADVS-12-e03603-s003.docx]

**Supporting Information**

Supporting Information is available from the Wiley Online Library or from the author.

Table 1. List of primers and antibodies used - in this study

| **Primers** | | |
| --- | --- | --- |
| **Gene name** | **Upstream primer sequence (5'-3')** | **Downstream primer sequence (5'-3')** |
| Entpd1 (M) | TTCAAGTGGTGGCGTCCT | TGGCACTGTTCGTAGTCTCC |
| Entpd2 (M) | GAACAGGCACTTCGGGATG | CAGCAGGTAGTTGGCAGTCA |
| Entpd3 (M) | GCTTCTCAGCCCACTACATCT | GCCAGGACTCCCATAAACAC |
| Entpd4 (M) | CAACCCCAGCGTGAACTAT | GCTCAGAAGCGGAGAAATGT |
| Entpd5 (M) | GCTTTGAACCCTGCTATGC | CCGCCCTTTTCATAATCG |
| Entpd6 (M) | CTGGGCTTTCTGCTTACGCT | CACCTCCTTCACCTTTTGC |
| Entpd7 (M) | CTGCTCAGGATTACTGTGGC | AGGTTTGGGTAGTCGTAGGG |
| Entpd8 (M) | CTCCCAGTGCAGCTCAGAC | CCTTCACCAGGATGAGGACG |
| Nt5e (M) | TACGATGCTATGGCACTGGG | CACCGCCAACAGAGAGAACT |
| Ada (M) | GCATTTGGCATCAAGGTCCG | CCATTCTTTACTGCGCCCTCA |
| ACC1 (M) | TGGTCGTGACTGCTCTGTGC | GTAGCC GAGGGTTCAGTTCC |
| CHREBP (M) | TTACTGGAAGCGGCGCATCG | CCAAGCAGCACAGGCACCAC |
| FASN (M) | CTGCCACAACTCTGAGGACA | CGGATCACCTTCTTGAGAGC |
| LXR (M) | TGCCATCAGCATCTTCTCTG | GGCTCACCAGCTTCATTAGC |
| SCD1 (M) | ATGTGCCAGAGGAGCTGAGT | TGATCCACTGTTGCTTCTGC |
| SREBP1 (M) | ACTTCTGGAGACATCGCAAAC | GGTAGACAACAGCCGCATC |
| Acox1 (M) | CCGTCGAGAAATCGAGAACT | ATTGAGGCCAACAGGTTCCA |
| Cpt1α (M) | ACGTTGGACGAATCGGAACA | GGTGGCCATGACATACTCCC |
| PPARα (M) | GTGGGTGGTTGAATCGTGAG | GCAGTGGAGTTTGGGTTGG |
| Lcad (M) | GCATCAACATCGCAGAGAAA | ACGCTTGCTCTTCCCAAGTA |
| Mcad (M) | AACTAAACATGGGCCAGCGA | CAGCTGCGACTGTAGGTCTG |
| Scad (M) | ATGTGCCAGAGGAGCTGAGT | TGATCCACTGTTGCTTCTGC |
| ApoB (M) | TCACCATTTGCCCTCAACCTAA | GAAGGCTCTTTGGAAGTGTAAAC |
| Mtp (M) | ATCATCATTGGAGCCCTGGT | CATTCTTCAGGGCCAGCA |
| CD36 (M) | TGGTCAAGCCAGCTAGAAA | CCCAGTCTCATTTAGCCAC |
| Fatp1 (M) | CCGTATCCTCACGCATGTGT | CTCCATCGTGTCCTCATTGAC |
| Fatp2 (M) | GATGCCGTGTCCGTCTTTTAC | GACTTCAGACCTCCACGACTC |
| Fatp5 (M) | TCGGATCTGGGAATTCTACG | TTGGTTCTTTCGAACCTTGG |
| G6Pase (M) | AGGAAGGATGGAGGAAGGAA | TGGAACCAGATGGGAAAGAG |
| PEPCK (M) | ATCTTTGGTGGCCGTAGACCT | CCGAAGTTGTAGCCGAAGAA |
| Prl7d1 (M) | TCCTATCAAAGGCCGAAGCC | TTTGATCTTGCAAAGTGAAGTGT |
| Skint6 (M) | CCAAGTTCCTCTGTGCGTCT | GTCGTAGAGTTGTACGGCCT |
| Dpep1 (M) | TCCTGCGGACACTCTACCAT | ACACCCAAGCGGTTCATCTC |
| Amd-ps4 (M) | TGATCCTGAGGGCCATGCTAA | CATGCACTTCAGTTCCACAGC |
| Serpina6 (M) | GGAAACGTGGTGCCAAACTC | GGGATGGTGTCTCAACCTGG |
| Apoa5 (M) | CAGGCTTTTCGGCATGACAC | CATCGTGTGGATGGCTCAGT |
| Apoa1 (M) | CACCTGAAGACACTTGGCGA | GGCCTTGTCGATCACACTCT |
| Bglap3 (M) | TCCAAGCAGGAGGGCAATAA | TCAAGCTCACATAGCTCCCG |
| Ugt2a3 (M) | GACTGTGGGGAAAGCTGACA | AGAGTGCTGTTGGAAGGCTC |
| Ugt1a5 (M) | TTTCCCTGTGGGGCATTGTT | GGTCTAGTTCCGGTGTAGCG |
| Ugt3a2 (M) | TAGGGCTCATGCTGGGTACT | CCTCCTTGACCTTCGTTGCT |
| Il2rb (M) | TCAGAAATGGCTCTCCTCGC | AATCTCCGTCGAGCACTTCC |
| Ugt3a1 (M) | GCCTTGGATTTTGCTCGTCC | TGCCTGATAACCTTGGAGGC |
| Cyp2c70 (M) | GACAGGAACCACATGCCGTA | CTCCACATCCTGCGTTGTCT |
| Pcdhgb8 (M) | AACTACAGCGAGGGGACTCT | CGGAGGGACCATTTCTGGAG |
| Sema4f (M) | CGAGGCTGACTCCTATCTCA | CAGCTCGGGAGATAATCGGC |
| UCP1 (M) | GCCAGGCTTCCAGTACCATT | AAGCATTGTAGGTCCCCGTG |
| UCP1-1 (M) | TGCCATTCCTCTAAGACCATAGC | AAACGGCAGTCTGTGAGCAT |
| UCP1-2 (M) | GATGCTCACAGACTGCCGTT | TTCTGCTACTCAGCGTGGTC |
| UCP1-3 (M) | CTGGGACCACGCTGAGTAG | GGGGAGGGAATCCATGCAAAA |
| UCP1-4 (M) | TCTCGAAAAAGTTTAGGGCGTT | CCAAAGAGCTGCTAGTGGGA |
| ADM (M) | AGTCGTGGGAAGAGGGAACT | ATGTGGGCTTCGCTCTGATT |
| ADM-1 (M) | CAAGGCTGGTAGAGACTAGAGC | GCAAAACGGAGTTCCCCCT |
| ADM-2 (M) | TTTAGGAGGATAAGTAATCTCGGCG | GGGGTCACCATTCTCATCAGG |
| ADM-3 (M) | GCTGTGCCCCAGTCTTTTGA | CTGGGACTGAGGGGTTTCTC |
| ADM-4 (M) | AAACCCCTCAGTCCCAGTCA | ATAAGCGCTCAGGGGCGG |
| Hmgb1 (M) | TCTGAGTACCGCCCCAAAATC | GCAGCTTTCTTCTCATAGGGC |
| Mecp2 (M) | AAACGGGGTAGAAAGCCTGG | AACTGTGGTAGTGGTGGCGA |
| Nfib (M) | ACCCGTGCTGTGTCTTATCC | TGCAGGTTCACACCAGAGTTC |
| Cdc5l (M) | AATGCCACCCCAGGTAGAAC | AGCTTCACTGGAGAGTTCGC |
| **Gene name** | **Upstream primer sequence (5'-3')** | **Downstream primer sequence (5'-3')** |
| Nop2 (M) | CTATTGGCGCTACCCCTGAG | TTCGTCTTCACAGCAGGGTC |
| NONO (M) | GAAACACGAACCCTAGCGGA | GGCCCATCCGTATCTCTTGTT |
| Pnpla2 (M) | GGGTCCTGAGTCTTGGAATGG | AGTGAGTGGCTGGTGAAAGG |
| HSL (M) | GGAGCACTACAAACGCAACG | CGTTCAAATTCAGCCCCACG |
| LPL (M) | GTGGACATCGGAGAACTGCT | CCTCTCGATGACGAAGCTGG |
| CRLR(M) | GGGACGGAATCAATGCAGTA | CCGAAACCAGTGTCCATCTT |
| RAMP2(M) | GATCTCGGCTTGGTGTGACC | GGGACTCCGGAAGAGATTGG |
| RAMP3(M) | CCTGTCGGAGTTCATCGTGT | ATAGCCACAGTCAGCACGAC |
| **Gene name** | **Upstream primer sequence (5'-3')** | **Downstream primer sequence (5'-3')** |
| **Antibodies** | | |
| **Primary antibody** | **Source** | **Identifer** |
| anti-ENTPD5, WB | Proteintech | 26746-1-AP |
| anti-pAkt, WB | Cell Signaling Technology | 9271 |
| anti-Akt, WB | Cell Signaling Technology | 9272S |
| anti-G6Pase, WB | Santa Cruz | sc-25840 |
| anti-PEPCK, WB | Bioworld | BS6870 |
| anti-Fatty Acid Synthase, WB | Abcam | ab22759 |
| anti-UCP1, WB, IHC | Abclonal | A5857 |
| anti-PGC1α, WB | Abclonal | A11971 |
| anti-ADM, WB | Cloudclone | MAA220Hu22 |
| anti-CaM, WB | Abclonal | A10769 |
| anti-CaM, WB | Abcam | ab45689 |
| anti-JNK, WB | Immunoway | YT2439 |
| anti-p-JNK(Thr183/Tyr185), WB | Cell Signaling Technology | 9251 |
| anti-JUN, WB | Proteintech | 24909-1-AP |
| anti-MECP2, WB, CHIP | Proteintech | 10861-1-AP |
| anti-p54/nrb (A-11) , WB | Santa Cruz | sc-166702 |
| anti-NONO, WB, CHIP | Proteintech | 11058-1-AP |
| anti-ADM, WB | CLOUD-CLONE CORP | MAA220Hu22 |
| anti-AP1, WB | Immunoway | YN3243 |
| anti-p-AP1(S63), WB | Abcam | ab273448 |
| anti-FOXO1, WB, IF | Cell Signaling Technology | 2880 |
| anti-GAPDH, WB | Cell Signaling Technology | 5174 |
| **Secondary antibody** | **Source** | **Identifer** |
| HRP goat anti-rabbit IgG(H+L), WB | Biodragon Immunotechnologies | BF03008 |
| HRP goat anti-mouse IgG(H+L), WB | Biodragon Immunotechnologies | BF02001 |

Table 2. Clinical characteristics of human subjects with or without NAFLD

| **Number** | **Gender** | **Age**  **(year)** | **Height**  **(cm)** | **Weight**  **(kg)** | **BMI** | **ALT**  **(U/L)** | **AST**  **(U/L)** | **Albumin**  **(g/L)** | **CHO**  **(mmol/L)** | **TG**  **(mmol/L)** | **HDL-C**  **(mmol/L)** | **LDL-C**  **(mmol/L)** | **TP**  **(g/L)** | **Fatty liver** |
| --- | --- | --- | --- | --- | --- | --- | --- | --- | --- | --- | --- | --- | --- | --- |
| 1 | F | 65 | 155 | 62 | 25.8 | 11 | 23 | 45.5 | 5.16 | 0.7 | 2.1 | 2.72 | 81.5 | no |
| 2 | F | 42 | 160 | 58 | 22.6 | 16 | 18 | 46 | 4.71 | 1.08 | 1.34 | 2.74 | 81.5 | no |
| 3 | M | 63 | 178 | 60 | 18.9 | 15 | 17 | 36.4 | 4.68 | 1.56 | 0.85 | 3.03 | 62.1 | no |
| 4 | F | 57 | 160 | 72 | 28.1 | 65 | 60 | 39.1 | 5.33 | 1.41 | 1.12 | 3.25 | 69.3 | yes |
| 5 | M | 60 | 181 | 90 | 27.4 | 16 | 19 | 43.8 | 4.7 | 0.93 | 1.15 | 2.78 | 69.2 | yes |
| 6 | F | 54 | 159 | 54 | 21.3 | 11 | 21 | 43.8 | 6 | 2.28 | 1.07 | 4.11 | 77.3 | yes |

Table 3. Serum ADM concentration in adult overweight/obese patients

| Number | Gender | Age (year) | Height (cm) | Weight (kg) | BMI | ADM (pg/mL) | FBG (mmol/L) | HbA1c (％) |
| --- | --- | --- | --- | --- | --- | --- | --- | --- |
| 1 | F | 24 | 168 | 137 | 48.5 | 1798.138 | 4.89 | 5.8 |
| 2 | M | 29 | 177.5 | 130.3 | 41.4 | 1324.639 | 5.24 | 5.9 |
| 3 | M | 28 | 186.5 | 143.3 | 41.2 | 1116.145 | 5.17 | 6 |
| 4 | F | 39 | 160.5 | 105.1 | 40.8 | 1295.525 | 12.3 | 8.9 |
| 5 | F | 38 | 143.5 | 75.7 | 36.8 | 1452.836 | 4.08 | 5.1 |
| 6 | F | 29 | 164.5 | 97 | 35.8 | 1427.561 | 4.83 | 5.3 |
| 7 | M | 35 | 181.5 | 109.5 | 33.2 | 1780.768 | 4 | 5.2 |
| 8 | M | 41 | 181 | 104.5 | 31.9 | 1045.168 | 5.31 | 6.2 |
| 9 | M | 40 | 167 | 88.4 | 31.7 | 1331.331 | 5.48 | 5.7 |
| 10 | F | 32 | 165.5 | 84.8 | 31 | 1456.965 | 5.42 | 5.8 |
| 11 | F | 41 | 153.5 | 72.6 | 30.8 | 1410.735 | 4.51 | 5.6 |
| 12 | F | 46 | 164.5 | 82.9 | 30.6 | 1290.15 | 5.51 | - |
| 13 | F | - | 155 | 73.1 | 30.4 | 1733.638 | 4.58 | 5.4 |
| 14 | F | 25 | 156.5 | 74.1 | 30.3 | 1452.848 | 4.59 | 5.3 |
| 15 | F | 34 | 165.5 | 82.3 | 30 | 1560.998 | 4.71 | 5.8 |
| 16 | F | 40 | 167 | 83 | 29.8 | 1567.938 | 4.75 | 5.7 |
| 17 | F | 29 | 163 | 78.4 | 29.5 | 1242.306 | 4.94 | 5.8 |
| 18 | F | 32 | 151 | 66.8 | 29.3 | 1782.399 | 6.49 | 6.1 |
| 19 | M | 38 | 180 | 93.3 | 28.8 | 1470.973 | 5.67 | 5.9 |
| 20 | M | 28 | 177.5 | 88.3 | 28 | 1709.048 | 4.2 | 5.5 |
| 21 | F | 39 | 163.5 | 74.7 | 27.9 | 846.594 | 4.63 | 4.9 |
| 22 | F | 31 | 163.5 | 74.5 | 27.9 | 823.991 | 4.97 | 5.8 |
| 23 | F | 47 | 166 | 76.5 | 27.8 | 854.464 | 4.89 | 5.8 |
| 24 | F | 41 | 169 | 77.9 | 27.3 | 903.551 | 4.24 | 5.2 |
| 25 | M | 55 | 166.5 | 74.9 | 27 | 884.14 | 5.84 | 5.3 |
| 26 | F | 46 | 162.5 | 71.1 | 26.9 | 807.215 | 5.12 | 6.1 |
| 27 | F | 38 | 166 | 73.4 | 26.6 | 925.376 | 4.42 | 5.6 |
| 28 | F | 36 | 161 | 67.4 | 26 | 957.598 | - | 5.2 |
| 29 | F | 27 | 176 | 75.9 | 24.5 | 844.401 | 5.09 | 5 |
| 30 | F | 31 | 166.5 | 67.2 | 24.2 | 912.598 | 5.32 | 5.8 |

Table 4. Serum ADM concentration in overweight/obese adolescents

| Number | Gender | Age (year) | Height (cm) | Weight (kg) | BMI | ADM (pg/mL) | FBG (mmol/L) | HbA1c (％) |
| --- | --- | --- | --- | --- | --- | --- | --- | --- |
| 1 | F | 11 | 152.4 | 62 | 26.69449783 | 1708.512 | 4.8 | 5.3 |
| 2 | F | 10 | 163.2 | 82.5 | 30.9751658 | 1669.638 | 4.8 | 5.4 |
| 3 | M | 10.5 | 148.5 | 58 | 26.30117108 | 1032.109 | 5.2 | 5.8 |
| 4 | M | 10.5 | 170.5 | 94.5 | 32.50746038 | 1327.549 | 4.8 | 6.1 |
| 5 | M | 11 | 155.6 | 59 | 24.36872609 | 1086.532 | 4.7 | 5.3 |
| 6 | F | 10.5 | 152.6 | 58 | 24.90685694 | 1273.126 | 4.6 | 5.6 |
| 7 | M | 10.5 | 165.5 | 74 | 27.01691295 | 1545.242 | 5.2 | 5.8 |
| 8 | M | 10.5 | 147.5 | 55.5 | 25.50991095 | 1280.901 | 4.8 | 5.9 |
| 9 | M | 10 | 150 | 48 | 21.33333333 | 884.388 | 4.9 | 5.4 |
| 10 | M | 10 | 145.5 | 50.9 | 24.04317379 | 1001.01 | 5.5 | 5.2 |
| 11 | M | 10 | 145.2 | 50.5 | 23.95290243 | 1179.829 | 4.8 | 5.6 |
| 12 | M | 10 | 145.1 | 49.5 | 23.51096062 | 923.262 | 4.8 | 5.5 |
| 13 | F | 10 | 136.7 | 42.5 | 22.7432173 | 1008.784 | 5 | 5.6 |
| 14 | M | 10.5 | 155.5 | 54.5 | 22.53905563 | 1024.334 | 5 | 5.2 |
| 15 | M | 10 | 150 | 51 | 22.66666667 | 1164.279 | 5 | 5.4 |
| 16 | M | 12 | 163.6 | 85.5 | 31.94475165 | 1288.675 | 4.6 | 5.2 |
| 17 | M | 11.5 | 162.8 | 70.5 | 26.59991911 | 1397.522 | 4.8 | 5.7 |
| 18 | F | 11 | 156.4 | 52.9 | 21.62629758 | 1179.829 | 4.9 | 5.4 |
| 19 | M | 11.5 | 149 | 63 | 28.37710013 | 1475.269 | 4.4 | 5.5 |
| 20 | M | 11.5 | 160 | 81 | 31.640625 | 1296.45 | 4.5 | 5.9 |
| 21 | M | 11 | 159.4 | 67.5 | 26.56605936 | 1381.972 | 5.2 | 5.8 |
| 22 | F | 11.5 | 173.7 | 72 | 23.86342959 | 985.46 | 4.5 | 5.6 |
| 23 | F | 11.5 | 166.4 | 80.5 | 29.07295904 | 1117.631 | 4.7 | 5.5 |
| 24 | M | 11.5 | 171 | 80.5 | 27.52983824 | 1475.269 | 5.2 | 5.7 |
| 25 | F | 11.5 | 156.5 | 79 | 32.2551011 | 1296.45 | 5.5 | 5.8 |
| 26 | M | 11 | 148.5 | 60 | 27.20810802 | 1304.225 | 5.3 | 5.6 |
| 27 | F | 11 | 164 | 61.5 | 22.86585366 | 938.8115 | 4.9 | 5.6 |
| 28 | F | 12 | 164.8 | 60.5 | 22.27619238 | 1094.307 | 4.2 | 5.1 |
| 29 | M | 11.5 | 174.2 | 92.8 | 30.58099945 | 1140.955 | 5.2 | 5.4 |
| 30 | F | 11 | 168.5 | 75.1 | 26.45088008 | 1047.658 | 4.4 | 5 |
| 31 | M | 11 | 170 | 77 | 26.64359862 | 931.0365 | 4.8 | 5.7 |
| 32 | M | 10.5 | 154.5 | 54.5 | 22.83176758 | 1164.279 | 5.4 | 5.5 |
| 33 | F | 10 | 151.9 | 45 | 19.50279995 | 1094.307 | 5.6 | 5.5 |
| 34 | F | 10 | 136.5 | 40.5 | 21.73650525 | 1001.01 | 4.3 | 5.5 |
| 35 | M | 10.5 | 156.1 | 60.5 | 24.82844774 | 1451.945 | 5.1 | 5.5 |
| 36 | M | 10 | 145.4 | 50.5 | 23.88705256 | 1319.774 | 4.5 | 5.4 |
| 37 | M | 10.5 | 159 | 61 | 24.12879237 | 1008.784 | 5.3 | 5.4 |
| 38 | M | 10 | 157.1 | 71 | 28.76775548 | 1102.081 | 5.1 | 5.7 |

Table 5. Differential genes in RNA- sequence

| Gene_id | Gene name | FC(AD/GFP) | Log2FC(AD/GFP) | Pvalue | Padjust | Significant | Regulate | **AD**  2_1 | **AD**  2_2 | **AD**  2_3 | **AD**  2_4 | **GFP**  1_1 | **GFP**  1_2 | **GFP**  1_3 | **GFP**  1_4 |
| --- | --- | --- | --- | --- | --- | --- | --- | --- | --- | --- | --- | --- | --- | --- | --- |
| ENSMUSG00000000627 | Sema4f | 3.398368 | 1.764842 | 0.032384 | 0.404313 | yes | up | 0.08 | 0.18 | 0.24 | 0.32 | 0.05 | 0.01 | 0.07 | 0.05 |
| ENSMUSG00000000730 | Dnmt3l | 0.098983 | -3.33668 | 0.037859 | 1 | yes | down | 0 | 0.27 | 0 | 0 | 0.17 | 0.1 | 0.05 | 0.26 |
| ENSMUSG00000000817 | Fasl | 0.092623 | -3.43249 | 0.011713 | 1 | yes | down | 0 | 0.03 | 0.03 | 0 | 0.15 | 0.05 | 0.21 | 0.23 |
| ENSMUSG00000001029 | Icam2 | 0.357182 | -1.48527 | 0.02779 | 0.389377 | yes | down | 0.02 | 0.62 | 1.09 | 0.28 | 0.45 | 0.92 | 0.98 | 1.57 |
| ENSMUSG00000001333 | Sync | 0.392344 | -1.34981 | 0.009891 | 0.284938 | yes | down | 0.12 | 0.28 | 0.39 | 0.08 | 0.58 | 0.59 | 0.25 | 0.48 |
| ENSMUSG00000001670 | Tat | 0.446649 | -1.16279 | 6.15E-06 | 0.00853 | yes | down | 3.98 | 2.42 | 3.21 | 3 | 5.77 | 5 | 7.69 | 5.99 |
| ENSMUSG00000003477 | Inmt | 0.367573 | -1.4439 | 0.009147 | 0.280171 | yes | down | 5.11 | 2.26 | 2.77 | 2.21 | 4.35 | 5.39 | 10.9 | 8.39 |
| ENSMUSG00000003555 | Cyp17a1 | 0.358982 | -1.47802 | 0.007129 | 0.267419 | yes | down | 0.87 | 0.21 | 0.1 | 0.48 | 1.04 | 0.95 | 1.19 | 1.77 |
| ENSMUSG00000005338 | Cadm3 | 0.216741 | -2.20595 | 0.024976 | 1 | yes | down | 0.06 | 0.04 | 0.02 | 0 | 0.42 | 0.22 | 0.07 | 0.14 |
| ENSMUSG00000006344 | Ggt5 | 0.098231 | -3.34768 | 0.007292 | 1 | yes | down | 0.01 | 0.02 | 0 | 0.01 | 0.11 | 0.01 | 0.52 | 0.33 |
| ENSMUSG00000009093 | Gstt4 | 0.473783 | -1.0777 | 0.018544 | 0.342624 | yes | down | 0.28 | 0.92 | 1.13 | 0.85 | 1.66 | 2.05 | 1.06 | 0.89 |
| ENSMUSG00000010492 | Uckl1os | 0.115457 | -3.11457 | 0.014803 | 0.318126 | yes | down | 0 | 2.14 | 0.28 | 0.75 | 2.13 | 0.63 | 3.23 | 0.94 |
| ENSMUSG00000015981 | Stk32c | 0.09085 | -3.46037 | 0.010552 | 1 | yes | down | 0.02 | 0.03 | 0 | 0 | 0.27 | 0.07 | 0.08 | 0.14 |
| ENSMUSG00000016529 | Il10 | 26.14912 | 4.70869 | 0.006229 | 1 | yes | up | 0 | 0.22 | 0.65 | 0.14 | 0 | 0 | 0 | 0 |
| ENSMUSG00000017300 | Tnnc2 | 0.393081 | -1.3471 | 0.028139 | 0.38953 | yes | down | 0.44 | 0.92 | 0.26 | 0.54 | 1.23 | 1.52 | 0.65 | 1.39 |
| ENSMUSG00000018411 | Mapt | 0.492871 | -1.02072 | 0.036607 | 0.415004 | yes | down | 0.43 | 0.69 | 0.29 | 0.16 | 0.72 | 0.36 | 1.34 | 0.86 |
| ENSMUSG00000018893 | Mb | 0.321246 | -1.63825 | 0.0s40142 | 1 | yes | down | 0 | 0.76 | 0.41 | 0.45 | 0.64 | 0.69 | 0.55 | 0.87 |
| ENSMUSG00000019278 | Dpep1 | 0.050389 | -4.31075 | 0.01168 | 1 | yes | down | 0.08 | 0 | 0 | 0 | 0 | 0.43 | 0.17 | 0.02 |
| ENSMUSG00000019836 | Amd-ps4 | 0.068342 | -3.87109 | 0.026164 | 1 | yes | down | 0 | 0 | 0 | 0 | 0.21 | 0.25 | 0.11 | 0.08 |
| ENSMUSG00000021135 | Slc10a1 | 0.48821 | -1.03443 | 4.40E-05 | 0.027259 | yes | down | 8.5 | 3.04 | 3.02 | 2.68 | 10.27 | 8.17 | 13.01 | 10.28 |
| ENSMUSG00000021210 | Akr1c6 | 0.479036 | -1.06179 | 0.000259 | 0.07797 | yes | down | 4.94 | 2.74 | 3.11 | 3.33 | 5.62 | 5.53 | 6.98 | 7.79 |
| ENSMUSG00000021217 | Tshz3 | 0.38302 | -1.38451 | 0.018718 | 0.343664 | yes | down | 0.03 | 0.08 | 0.07 | 0.06 | 0.22 | 0.13 | 0.15 | 0.05 |
| ENSMUSG00000024347 | Psd2 | 0.264246 | -1.92005 | 0.01029 | 1 | yes | down | 0.09 | 0.05 | 0.03 | 0.02 | 0.13 | 0.09 | 0.1 | 0.07 |
| ENSMUSG00000024770 | Lipn | 2.02616 | 1.018748 | 0.023812 | 0.369896 | yes | up | 1.01 | 1.84 | 1.02 | 1.31 | 1.46 | 0.4 | 0.58 | 0.22 |
| ENSMUSG00000025104 | Hdgfl3 | 0.268798 | -1.89541 | 0.022633 | 0.365106 | yes | down | 0.09 | 0 | 0.06 | 0.02 | 0.11 | 0.09 | 0.1 | 0.21 |
| ENSMUSG00000025396 | Hsd17b6 | 0.477648 | -1.06598 | 0.000347 | 0.094629 | yes | down | 6.85 | 5.22 | 4.79 | 3.64 | 7.44 | 7.52 | 10.73 | 10.16 |
| ENSMUSG00000025491 | Ifitm1 | 0.338173 | -1.56417 | 0.003987 | 0.240678 | yes | down | 0.15 | 0.59 | 0.64 | 0.75 | 2.04 | 1.06 | 1.38 | 1.8 |
| ENSMUSG00000025500 | Lmntd2 | 0.46945 | -1.09096 | 0.039887 | 0.425713 | yes | down | 0.27 | 0.21 | 0.38 | 0.16 | 0.65 | 0.6 | 0.51 | 0.15 |
| ENSMUSG00000025784 | Clec3b | 0.149912 | -2.73781 | 0.010521 | 1 | yes | down | 0 | 0.07 | 0.17 | 0.09 | 0.23 | 0.48 | 0.29 | 0.49 |
| ENSMUSG00000026114 | Cnga3 | 2.478388 | 1.309402 | 0.031431 | 0.401429 | yes | up | 0.16 | 0.39 | 0.15 | 0.18 | 0.11 | 0.11 | 0.04 | 0.1 |
| ENSMUSG00000026117 | Zap70 | 0.233601 | -2.09788 | 0.026408 | 1 | yes | down | 0.05 | 0.08 | 0.05 | 0.05 | 0.22 | 0.09 | 0.34 | 0.29 |
| ENSMUSG00000027368 | Dusp2 | 3.578672 | 1.839424 | 0.038499 | 1 | yes | up | 0.3 | 0.28 | 0.27 | 0.22 | 0.18 | 0.03 | 0.06 | 0 |
| ENSMUSG00000027377 | Mall | 0.059454 | -4.07207 | 0.046751 | 1 | yes | down | 0 | 0 | 0 | 0 | 0.03 | 0.25 | 0 | 0.03 |
| ENSMUSG00000027481 | Bpifb2 | 0.468293 | -1.09452 | 0.023455 | 0.367442 | yes | down | 0.27 | 0.19 | 0.54 | 0.23 | 0.74 | 0.45 | 0.54 | 0.55 |
| ENSMUSG00000027513 | Pck1 | 0.334963 | -1.57793 | 0.000736 | 0.147757 | yes | down | 3.39 | 1.48 | 2.53 | 4.84 | 4.39 | 6.68 | 6.92 | 5.21 |
| ENSMUSG00000027584 | Oprl1 | 0.242158 | -2.04598 | 0.011531 | 1 | yes | down | 0.08 | 0.02 | 0.21 | 0.15 | 0.19 | 0.11 | 0.17 | 0.36 |
| ENSMUSG00000027716 | Trpc3 | 0.058302 | -4.10031 | 0.036705 | 1 | yes | down | 0 | 0 | 0 | 0 | 0.04 | 0 | 0.01 | 0.09 |
| ENSMUSG00000027761 | Aadac | 0.468318 | -1.09444 | 0.013425 | 0.309075 | yes | down | 13.68 | 6.33 | 7.23 | 7.47 | 13.26 | 13.54 | 22.7 | 16.25 |
| ENSMUSG00000027855 | Sycp1 | 0.043584 | -4.52005 | 0.003007 | 1 | yes | down | 0 | 0 | 0 | 0 | 0.22 | 0.03 | 0.07 | 0.07 |
| ENSMUSG00000027985 | Lef1 | 0.055059 | -4.18288 | 0.029191 | 1 | yes | down | 0 | 0 | 0 | 0 | 0.08 | 0.02 | 0.55 | 0 |
| ENSMUSG00000028005 | Gucy1b1 | 0.416403 | -1.26395 | 0.041154 | 0.429726 | yes | down | 0.1 | 0.34 | 0.13 | 0.05 | 0.3 | 0.3 | 0.33 | 0.06 |
| ENSMUSG00000028137 | Celf3 | 0.274333 | -1.866 | 0.033374 | 1 | yes | down | 0.08 | 0.02 | 0.06 | 0.1 | 0.25 | 0.21 | 0.23 | 0.35 |
| ENSMUSG00000028240 | Cyp7a1 | 0.281711 | -1.82771 | 0.024205 | 0.371548 | yes | down | 0.14 | 0.05 | 0.03 | 0 | 0.18 | 0.08 | 0.27 | 0.16 |
| ENSMUSG00000028441 | 1110017D15Rik | 0.414849 | -1.26934 | 0.038266 | 0.418888 | yes | down | 0.7 | 0.89 | 0.5 | 0.31 | 2.21 | 0.92 | 2.19 | 0.29 |
| ENSMUSG00000028845 | Tekt2 | 0.447475 | -1.16012 | 0.000334 | 0.09284 | yes | down | 1.16 | 1.33 | 0.79 | 0.6 | 2.16 | 2.34 | 1.69 | 1.39 |
| ENSMUSG00000030236 | Slco1b2 | 0.342131 | -1.54738 | 0.007322 | 0.26837 | yes | down | 2.07 | 0.73 | 0.93 | 0.71 | 2.94 | 2 | 3.85 | 2.49 |
| ENSMUSG00000030237 | Slco1a4 | 0.205266 | -2.28443 | 0.000139 | 0.05644 | yes | down | 0.14 | 0.05 | 0.26 | 0.1 | 0.38 | 0.36 | 0.39 | 0.28 |
| ENSMUSG00000030470 | Csrp3 | 0.229358 | -2.12433 | 0.00911 | 0.280171 | yes | down | 0.31 | 0.07 | 0.34 | 0.16 | 0.31 | 0.41 | 1.34 | 1.11 |
| ENSMUSG00000030790 | Adm | 0.349226 | -1.51777 | 6.19E-05 | 0.030813 | yes | down | 14.3 | 12.14 | 8.87 | 10.51 | 23.65 | 19.82 | 37.56 | 30.65 |
| ENSMUSG00000031284 | Pak3 | 0.287019 | -1.80078 | 0.005015 | 0.249142 | yes | down | 0.03 | 0.05 | 0.01 | 0.03 | 0.22 | 0.1 | 0.06 | 0.04 |
| ENSMUSG00000032024 | Clmp | 0.493562 | -1.0187 | 0.042801 | 0.434662 | yes | down | 0.55 | 0.79 | 0.4 | 0.26 | 0.19 | 0.82 | 0.37 | 0.52 |
| ENSMUSG00000032079 | Apoa5 | 0.296284 | -1.75495 | 0.003725 | 0.238367 | yes | down | 14.91 | 5.53 | 5.22 | 8.68 | 22.17 | 23.63 | 31.38 | 18.3 |
| ENSMUSG00000032083 | Apoa1 | 0.429682 | -1.21866 | 0.031496 | 0.401429 | yes | down | 800.57 | 269.89 | 281.2 | 296.92 | 679.56 | 733.25 | 1187.85 | 849.83 |
| ENSMUSG00000032356 | Rasgrf1 | 17.30316 | 4.112964 | 0.026617 | 1 | yes | up | 0.07 | 0.02 | 0 | 0.06 | 0 | 0 | 0 | 0 |
| ENSMUSG00000032492 | Pth1r | 0.327895 | -1.6087 | 0.008587 | 0.279503 | yes | down | 0.1 | 0.17 | 0.12 | 0.32 | 0.35 | 0.5 | 0.59 | 0.3 |
| ENSMUSG00000032495 | Lrrc2 | 0.399689 | -1.32305 | 0.04527 | 0.439129 | yes | down | 0.09 | 0.31 | 0.02 | 0.23 | 0.23 | 0.39 | 0.11 | 0.1 |
| ENSMUSG00000032523 | Hhatl | 0.489017 | -1.03204 | 0.035458 | 0.413092 | yes | down | 0.15 | 0.47 | 0.22 | 0.25 | 0.65 | 0.67 | 1.1 | 0.6 |
| ENSMUSG00000032978 | Guca2b | 2.578889 | 1.36675 | 0.020589 | 0.351844 | yes | up | 0.89 | 3.1 | 2.75 | 4.8 | 1.09 | 0.97 | 0.23 | 1.49 |
| ENSMUSG00000033722 | BC034090 | 0.349732 | -1.51568 | 0.011562 | 0.296865 | yes | down | 0.18 | 0.05 | 0.21 | 0.24 | 0.61 | 0.72 | 0.39 | 0.81 |
| ENSMUSG00000033794 | Lpcat2b | 8.828784 | 3.142215 | 0.047787 | 1 | yes | up | 0.05 | 0.07 | 0.02 | 0.08 | 0 | 0.02 | 0 | 0 |
| ENSMUSG00000034570 | Inpp5j | 0.395252 | -1.33915 | 0.048759 | 0.44568 | yes | down | 0.08 | 0.07 | 0.14 | 0.04 | 0.15 | 0.21 | 0.26 | 0.07 |
| ENSMUSG00000034755 | Pcdh11x | 2.476195 | 1.308125 | 0.011586 | 0.296865 | yes | up | 2.33 | 2.92 | 4.73 | 1.18 | 0.27 | 1.22 | 1.28 | 0.9 |
| ENSMUSG00000034958 | Atcay | 0.325455 | -1.61947 | 0.040839 | 1 | yes | down | 0.05 | 0.03 | 0.02 | 0.06 | 0.07 | 0.08 | 0.14 | 0.11 |
| ENSMUSG00000035780 | Ugt2a3 | 0.47554 | -1.07236 | 0.035057 | 0.412494 | yes | down | 4.85 | 2.19 | 1.58 | 1.92 | 5.21 | 3.85 | 6.06 | 4.94 |
| ENSMUSG00000036185 | Sapcd1 | 0.078148 | -3.67764 | 0.011945 | 1 | yes | down | 0.08 | 0 | 0 | 0.01 | 0.28 | 0.2 | 0.22 | 0.19 |
| ENSMUSG00000036551 | Akap14 | 0.236124 | -2.08239 | 0.021489 | 1 | yes | down | 0.05 | 0.09 | 0 | 0.06 | 0.32 | 0.23 | 0.11 | 0.15 |
| ENSMUSG00000036770 | Stpg3 | 0.380053 | -1.39573 | 0.017025 | 0.334923 | yes | down | 0.21 | 0.37 | 0.2 | 0.18 | 0.24 | 0.55 | 1.09 | 0.51 |
| ENSMUSG00000037477 | Tbx10 | 0.265902 | -1.91103 | 0.030761 | 1 | yes | down | 0.05 | 0.18 | 0.03 | 0.1 | 0.06 | 0.15 | 0.56 | 0.33 |
| ENSMUSG00000037996 | Slc24a2 | 0.305163 | -1.71235 | 0.008529 | 0.279066 | yes | down | 0.2 | 0.41 | 0.28 | 0 | 0.75 | 0.74 | 0.24 | 0.84 |
| ENSMUSG00000038354 | Ankrd35 | 0.333683 | -1.58345 | 0.000431 | 0.110459 | yes | down | 0.24 | 0.2 | 0.14 | 0.13 | 0.61 | 0.42 | 0.64 | 0.24 |
| ENSMUSG00000038453 | Srcin1 | 0.474984 | -1.07405 | 0.040779 | 0.428816 | yes | down | 0.04 | 0.07 | 0.04 | 0.1 | 0.23 | 0.53 | 0.54 | 0.07 |
| ENSMUSG00000038917 | 3930402G23Rik | 0.407365 | -1.2956 | 0.036998 | 0.415004 | yes | down | 0.11 | 0.2 | 0.33 | 0.15 | 0.36 | 0.09 | 0.21 | 0.21 |
| ENSMUSG00000040035 | Disp2 | 0.422259 | -1.2438 | 0.028008 | 0.389377 | yes | down | 0.03 | 0.05 | 0.05 | 0.05 | 0.13 | 0.06 | 0.1 | 0.08 |
| ENSMUSG00000040797 | Iqsec3 | 0.21234 | -2.23555 | 0.01555 | 1 | yes | down | 0.01 | 0.01 | 0.01 | 0.02 | 0.06 | 0.06 | 0.03 | 0.04 |
| ENSMUSG00000044296 | Zfp879 | 3.135916 | 1.648887 | 0.015166 | 0.322514 | yes | up | 0.29 | 0.33 | 0.25 | 0.25 | 0.02 | 0.11 | 0.12 | 0.05 |
| ENSMUSG00000044306 | 4930500M09Rik | 0.13177 | -2.92391 | 0.013213 | 1 | yes | down | 0.22 | 0 | 0.14 | 0.13 | 0.67 | 0.23 | 0.22 | 1.67 |
| ENSMUSG00000044337 | Ackr3 | 6.168564 | 2.624935 | 0.021622 | 1 | yes | up | 0.04 | 0.1 | 0.1 | 0.25 | 0.02 | 0 | 0.05 | 0 |
| ENSMUSG00000044359 | P2ry4 | 5.16022 | 2.367433 | 0.000568 | 0.129595 | yes | up | 0.1 | 0.19 | 0.19 | 0.19 | 0.02 | 0.01 | 0.04 | 0.03 |
| ENSMUSG00000044646 | Zbtb7c | 0.494229 | -1.01675 | 0.005495 | 0.251439 | yes | down | 0.41 | 0.28 | 0.28 | 0.18 | 0.53 | 0.74 | 0.72 | 0.54 |
| ENSMUSG00000045345 | 9530056K15Rik | 0.379198 | -1.39898 | 0.030575 | 0.398227 | yes | down | 0.08 | 0.08 | 0.09 | 0.08 | 0.14 | 0.31 | 0.19 | 0.13 |
| ENSMUSG00000046295 | Ankle1 | 0.466947 | -1.09867 | 0.004063 | 0.240678 | yes | down | 0.26 | 0.41 | 0.32 | 0.72 | 1.01 | 0.76 | 0.46 | 0.7 |
| ENSMUSG00000047040 | Prr15l | 0.368288 | -1.44109 | 0.036589 | 1 | yes | down | 0.05 | 0.04 | 0.39 | 0.14 | 0.38 | 0.49 | 0.11 | 0.29 |
| ENSMUSG00000048126 | Col6a3 | 0.404571 | -1.30553 | 0.0151 | 0.322514 | yes | down | 0.05 | 0.29 | 0.3 | 0.17 | 0.11 | 0.08 | 0.4 | 0.09 |
| ENSMUSG00000048794 | Cfap100 | 0.251356 | -1.9922 | 0.001171 | 0.176383 | yes | down | 0.04 | 0.07 | 0.24 | 0.06 | 0.25 | 0.34 | 0.51 | 0.19 |
| ENSMUSG00000049103 | Ccr2 | 0.190961 | -2.38865 | 0.041971 | 1 | yes | down | 0.02 | 0 | 0.03 | 0.02 | 0.13 | 0.15 | 0.05 | 0.01 |
| ENSMUSG00000049152 | Ugt3a2 | 0.467089 | -1.09823 | 0.015698 | 0.32722 | yes | down | 6.26 | 2.47 | 2.63 | 3.35 | 6.81 | 6.56 | 8.71 | 6.3 |
| ENSMUSG00000049719 | Prss46 | 0.168667 | -2.56775 | 0.013321 | 1 | yes | down | 0.04 | 0.05 | 0.11 | 0.05 | 0.67 | 0.09 | 0.4 | 0.14 |
| ENSMUSG00000049902 | 4921517D22Rik | 0.09471 | -3.40035 | 0.035105 | 1 | yes | down | 0.01 | 0 | 0 | 0 | 0.04 | 0.11 | 0.03 | 0.02 |
| ENSMUSG00000050463 | Krt78 | 0.374618 | -1.41651 | 0.000207 | 0.069179 | yes | down | 0.28 | 0.19 | 0.27 | 0.17 | 0.53 | 0.56 | 0.66 | 0.39 |
| ENSMUSG00000050556 | Kcnb1 | 0.226357 | -2.14333 | 0.012205 | 1 | yes | down | 0.05 | 0.04 | 0 | 0.13 | 0.18 | 0.07 | 0.11 | 0.1 |
| ENSMUSG00000051067 | Lingo3 | 0.195054 | -2.35806 | 0.000636 | 0.136245 | yes | down | 0.09 | 0.06 | 0 | 0.09 | 0.2 | 0.14 | 0.33 | 0.4 |
| ENSMUSG00000051457 | Spn | 2.109966 | 1.07722 | 0.020939 | 0.352814 | yes | up | 0.37 | 0.34 | 0.28 | 0.22 | 0.25 | 0.12 | 0.11 | 0.12 |
| ENSMUSG00000051940 | 5031410I06Rik | 25.85666 | 4.692464 | 0.008801 | 1 | yes | up | 0.01 | 0.14 | 0.24 | 0 | 0 | 0 | 0 | 0 |
| ENSMUSG00000053603 | 4930442H23Rik | 0.442331 | -1.1768 | 0.045976 | 0.440788 | yes | down | 0.25 | 0.33 | 0.39 | 0.15 | 0.75 | 0.4 | 0.34 | 0.68 |
| ENSMUSG00000054304 | D130007C19Rik | 0.379018 | -1.39966 | 0.011185 | 0.293574 | yes | down | 0.1 | 0.21 | 0.21 | 0.36 | 0.55 | 0.58 | 0.4 | 0.43 |
| ENSMUSG00000054422 | Fabp1 | 0.33335 | -1.58489 | 0.002742 | 0.219417 | yes | down | 133.21 | 47.23 | 51.61 | 45.68 | 168.86 | 167.71 | 233.05 | 188.52 |
| ENSMUSG00000055022 | Cntn1 | 0.48852 | -1.03351 | 0.03717 | 0.415004 | yes | down | 0.08 | 0.09 | 0.09 | 0.09 | 0.17 | 0.21 | 0.1 | 0.14 |
| ENSMUSG00000055407 | Map6 | 0.478904 | -1.06219 | 0.035268 | 0.413092 | yes | down | 0.12 | 0.11 | 0.21 | 0.2 | 0.19 | 0.84 | 0.21 | 0.28 |
| ENSMUSG00000055805 | Fmnl1 | 0.354673 | -1.49544 | 0.005221 | 0.249142 | yes | down | 0.21 | 0.08 | 0.17 | 0.16 | 0.25 | 0.44 | 0.31 | 0.46 |
| ENSMUSG00000056043 | Rgs9bp | 0.166501 | -2.5864 | 0.017424 | 1 | yes | down | 0.01 | 0 | 0.02 | 0.01 | 0.03 | 0.05 | 0.02 | 0.06 |
| ENSMUSG00000056947 | Mab21l1 | 0.072069 | -3.79448 | 0.032408 | 1 | yes | down | 0 | 0 | 0 | 0 | 0.02 | 0.04 | 0.06 | 0.06 |
| ENSMUSG00000057244 | Gm6139 | 0.040636 | -4.6211 | 0.006613 | 1 | yes | down | 0 | 0 | 0 | 0 | 0.52 | 0.45 | 0.28 | 0 |
| ENSMUSG00000057454 | Lypd3 | 0.192679 | -2.37573 | 0.001847 | 0.193013 | yes | down | 0.09 | 0.1 | 0 | 0.14 | 0.32 | 0.35 | 0.55 | 0.26 |
| ENSMUSG00000058297 | Spock2 | 0.27466 | -1.86428 | 0.025736 | 1 | yes | down | 0.03 | 0.04 | 0.03 | 0.01 | 0.04 | 0.05 | 0.05 | 0.34 |
| ENSMUSG00000058740 | Kcnt1 | 0.086248 | -3.53536 | 0.014311 | 1 | yes | down | 0.01 | 0 | 0 | 0.01 | 0.02 | 0.01 | 0.21 | 0.1 |
| ENSMUSG00000058812 | 0610039K10Rik | 0.069278 | -3.85146 | 0.034091 | 1 | yes | down | 0 | 0 | 0 | 0 | 0.42 | 0.13 | 0.06 | 0.06 |
| ENSMUSG00000060565 | Gm5591 | 0.222197 | -2.17009 | 0.004674 | 0.245574 | yes | down | 0.13 | 0.03 | 0.02 | 0.02 | 0.24 | 0.33 | 0.21 | 0.1 |
| ENSMUSG00000060568 | Fam78b | 0.218586 | -2.19373 | 0.004192 | 0.240678 | yes | down | 0.06 | 0.02 | 0.08 | 0.05 | 0.06 | 0.18 | 0.24 | 0.19 |
| ENSMUSG00000060613 | Cyp2c70 | 0.481983 | -1.05295 | 0.000554 | 0.128081 | yes | down | 6.63 | 4.05 | 3.39 | 3.56 | 6.81 | 6.92 | 8.8 | 9.21 |
| ENSMUSG00000060807 | Serpina6 | 0.287448 | -1.79863 | 0.040934 | 0.428816 | yes | down | 2.18 | 0.24 | 0.39 | 0.26 | 2.12 | 2.23 | 3.57 | 2.27 |
| ENSMUSG00000061906 | Ugt2b38 | 0.141213 | -2.82406 | 0.001988 | 1 | yes | down | 0.11 | 0.06 | 0 | 0 | 0.2 | 0.26 | 0.29 | 0.27 |
| ENSMUSG00000064358 | mt-Co3 | 0.372928 | -1.42303 | 0.012272 | 0.302911 | yes | down | 1810.13 | 946.95 | 660.8 | 933.47 | 1713.21 | 2192.41 | 3725.15 | 2565.32 |
| ENSMUSG00000064360 | mt-Nd3 | 0.450171 | -1.15145 | 0.046903 | 0.442121 | yes | down | 1030.68 | 404.96 | 414.49 | 361.94 | 631.02 | 1042.13 | 1523.39 | 1144.29 |
| ENSMUSG00000066902 | Rps23-ps2 | 14.93249 | 3.900383 | 0.049995 | 1 | yes | up | 0.3 | 0 | 0.83 | 1.95 | 0 | 0 | 0 | 0 |
| ENSMUSG00000067058 | Rps15a-ps5 | 12.1492 | 3.60279 | 0.000915 | 1 | yes | up | 2.1 | 2.12 | 4.66 | 3.26 | 0.33 | 0.57 | 0 | 0 |
| ENSMUSG00000067771 | Pwwp4a | 0.269332 | -1.89254 | 0.001721 | 0.190794 | yes | down | 0.26 | 0.19 | 0 | 0.28 | 0.57 | 0.79 | 0.78 | 0.27 |
| ENSMUSG00000068227 | Il2rb | 0.361107 | -1.4695 | 0.023019 | 0.366876 | yes | down | 0.05 | 0.05 | 0.09 | 0.03 | 0.14 | 0.09 | 0.12 | 0.12 |
| ENSMUSG00000068262 | Gm5879 | 0.472443 | -1.08179 | 0.045356 | 0.439441 | yes | down | 0.43 | 0.56 | 0.24 | 0.32 | 0.61 | 0.49 | 0.85 | 0.77 |
| ENSMUSG00000069188 | Gm13192 | 0.057181 | -4.12832 | 0.001748 | 1 | yes | down | 0 | 0 | 4.29 | 0 | 34.13 | 5.54 | 4.33 | 10.5 |
| ENSMUSG00000069303 | H2bc24 | 0.293636 | -1.7679 | 0.009824 | 0.284938 | yes | down | 0.16 | 0 | 0.11 | 0.27 | 0.79 | 0.71 | 0.44 | 0.25 |
| ENSMUSG00000069305 | H4c18 | 0.422385 | -1.24337 | 0.01621 | 0.331414 | yes | down | 1.55 | 4.7 | 1.78 | 3.89 | 7.3 | 2.3 | 7.84 | 6 |
| ENSMUSG00000071343 | Gm10327 | 0.192729 | -2.37536 | 0.030265 | 1 | yes | down | 0.12 | 0.09 | 0.17 | 0 | 0.1 | 0.38 | 0.57 | 0.33 |
| ENSMUSG00000071745 | Pwwp4b | 4.574845 | 2.193723 | 0.007275 | 0.26837 | yes | up | 0.53 | 0.48 | 0.62 | 0.15 | 0.25 | 0.13 | 0 | 0 |
| ENSMUSG00000072664 | Ugt3a1 | 0.490702 | -1.02708 | 0.019059 | 0.345031 | yes | down | 1.14 | 1.07 | 4.17 | 0.57 | 1.49 | 2.09 | 1.88 | 1.05 |
| ENSMUSG00000073679 | Mxra8os | 0.067271 | -3.89388 | 0.022637 | 1 | yes | down | 0 | 0 | 0 | 0 | 0.45 | 0.2 | 0.19 | 0.2 |
| ENSMUSG00000074417 | Pira12 | 0.032205 | -4.95656 | 0.000572 | 1 | yes | down | 0 | 0 | 0 | 0 | 0.08 | 0.23 | 0.11 | 0.07 |
| ENSMUSG00000074489 | Bglap3 | 0.440213 | -1.18373 | 0.001129 | 0.172536 | yes | down | 1.74 | 0.97 | 0.88 | 0.93 | 2.53 | 2.42 | 2.29 | 1.64 |
| ENSMUSG00000074491 | Clec4g | 0.269786 | -1.89011 | 0.020581 | 1 | yes | down | 0.16 | 0.09 | 0.05 | 0.15 | 0.19 | 0.4 | 0.6 | 0.27 |
| ENSMUSG00000074677 | Sirpb1c | 3.452398 | 1.787599 | 0.046188 | 1 | yes | up | 0.42 | 0.25 | 0.45 | 0.21 | 0 | 0.07 | 0.18 | 0.13 |
| ENSMUSG00000075405 | 9430097D07Rik | 0.140381 | -2.83258 | 0.010541 | 1 | yes | down | 0.04 | 0 | 0.04 | 0.06 | 0.12 | 0.09 | 0.26 | 0.4 |
| ENSMUSG00000076543 | Igkv4-74 | 0.059248 | -4.07708 | 0.015444 | 1 | yes | down | 0 | 0 | 0 | 0 | 0.32 | 0.83 | 0.78 | 1.15 |
| ENSMUSG00000078238 | Gm12854 | 17.10922 | 4.096702 | 0.014652 | 1 | yes | up | 7.27 | 5.11 | 0.92 | 0 | 0.91 | 0 | 0 | 0 |
| ENSMUSG00000078588 | Ccdc24 | 0.343805 | -1.54034 | 0.000277 | 0.080954 | yes | down | 0.37 | 0.24 | 1.28 | 0.73 | 1.07 | 0.63 | 0.54 | 0.57 |
| ENSMUSG00000078650 | G6pc | 0.306546 | -1.70583 | 9.50E-07 | 0.003165 | yes | down | 2.07 | 1.09 | 0.89 | 0.85 | 3.14 | 2.95 | 4.35 | 3.76 |
| ENSMUSG00000078689 | Mup6 | 0.067036 | -3.89892 | 0.009351 | 1 | yes | down | 0.06 | 0 | 0 | 0 | 0.36 | 0.07 | 0.43 | 0.2 |
| ENSMUSG00000078886 | Gm2026 | 2.098879 | 1.069619 | 0.035674 | 0.413092 | yes | up | 0.56 | 2.67 | 2.25 | 0.91 | 1.7 | 0.51 | 0.26 | 0.57 |
| ENSMUSG00000079049 | Serpinb1c | 0.119658 | -3.06301 | 0.046169 | 1 | yes | down | 0 | 0 | 0.08 | 0 | 0.27 | 0.29 | 0.06 | 0.12 |
| ENSMUSG00000079297 | Gm2223 | 4.487793 | 2.166006 | 0.006564 | 0.263612 | yes | up | 0.15 | 0.13 | 1.1 | 0.38 | 0 | 0.17 | 0.07 | 0.08 |
| ENSMUSG00000080859 | Rpl10-ps1 | 27.61354 | 4.787304 | 0.006761 | 1 | yes | up | 1 | 1.36 | 0.12 | 0 | 0 | 0 | 0 | 0 |
| ENSMUSG00000080896 | Gm14567 | 3.088919 | 1.627102 | 0.029311 | 1 | yes | up | 38.3 | 63.08 | 57.22 | 53.16 | 18.29 | 0 | 16.64 | 26.24 |
| ENSMUSG00000080993 | Gm12903 | 11.22983 | 3.489264 | 0.020704 | 1 | yes | up | 0.4 | 0.37 | 0.52 | 1.15 | 0 | 0.21 | 0 | 0 |
| ENSMUSG00000081494 | Gm14130 | 0.10034 | -3.31703 | 0.026026 | 1 | yes | down | 0 | 0.13 | 0 | 0 | 0.14 | 0.13 | 0.23 | 0.55 |
| ENSMUSG00000081953 | Gm12435 | 25.60876 | 4.678566 | 0.007312 | 1 | yes | up | 0.21 | 0 | 0.89 | 0.22 | 0 | 0 | 0 | 0 |
| ENSMUSG00000082286 | Pisd-ps1 | 0.498934 | -1.00308 | 0.000906 | 0.168481 | yes | down | 2.45 | 3.11 | 1.26 | 2.14 | 3.57 | 4.39 | 3.28 | 3.3 |
| ENSMUSG00000082289 | Gm15596 | 2.146653 | 1.102089 | 0.019222 | 0.345345 | yes | up | 1.95 | 2.1 | 1.08 | 2.22 | 1.01 | 0.9 | 0.63 | 0.5 |
| ENSMUSG00000082424 | Gm13292 | 2.843718 | 1.507778 | 0.027444 | 0.387689 | yes | up | 1.14 | 0.53 | 0.92 | 0.47 | 0.34 | 0.13 | 0.17 | 0.38 |
| ENSMUSG00000083534 | H2-M6-ps | 2.107994 | 1.075871 | 0.001176 | 0.176383 | yes | up | 1.25 | 2.31 | 2.08 | 1.93 | 0.51 | 0.82 | 0.59 | 1.1 |
| ENSMUSG00000083672 | Kpna2-ps | 2.458969 | 1.298053 | 0.000366 | 0.097642 | yes | up | 1.97 | 2.92 | 1.79 | 1.34 | 1.02 | 0.42 | 0.78 | 0.66 |
| ENSMUSG00000083863 | Gm13341 | 0.389924 | -1.35873 | 0.048004 | 0.44568 | yes | down | 0.59 | 0.22 | 0.5 | 0.63 | 0.6 | 0.95 | 1.12 | 1.52 |
| ENSMUSG00000084098 | Gm13422 | 6.213959 | 2.635513 | 0.040734 | 1 | yes | up | 1.08 | 0.54 | 1.19 | 0.95 | 0 | 0 | 0 | 0.52 |
| ENSMUSG00000084807 | Gm13073 | 0.357885 | -1.48243 | 0.04342 | 0.435723 | yes | down | 1.21 | 1.11 | 0.5 | 1.22 | 4.64 | 0.99 | 0.51 | 3.81 |
| ENSMUSG00000085083 | Gm11615 | 0.392758 | -1.34829 | 0.029291 | 0.39374 | yes | down | 0.14 | 0.74 | 0.78 | 0.36 | 1.38 | 0.51 | 1.03 | 1.18 |
| ENSMUSG00000085169 | Gm10785 | 2.076866 | 1.054408 | 0.009786 | 0.284938 | yes | up | 1.17 | 2.24 | 2.32 | 3.38 | 0.62 | 1.01 | 0.83 | 1.52 |
| ENSMUSG00000085636 | Gm11769 | 0.227853 | -2.13382 | 0.041117 | 1 | yes | down | 0.06 | 0.07 | 0 | 0.04 | 0.28 | 0.16 | 0.12 | 0.1 |
| ENSMUSG00000085774 | Gm13055 | 0.314913 | -1.66698 | 0.022186 | 0.361989 | yes | down | 0.07 | 0.08 | 0.13 | 0.15 | 0.09 | 0.26 | 0.3 | 0.45 |
| ENSMUSG00000086539 | Gm16759 | 2.180887 | 1.124915 | 0.006065 | 0.260899 | yes | up | 0.3 | 0.33 | 0.31 | 0.57 | 0.15 | 0.11 | 0.16 | 0.16 |
| ENSMUSG00000086578 | Gm13583 | 0.407181 | -1.29626 | 0.015529 | 0.325321 | yes | down | 0.2 | 0.37 | 0.54 | 0.25 | 0.42 | 0.99 | 0.81 | 0.39 |
| ENSMUSG00000086726 | Gm9458 | 15.04293 | 3.911013 | 0.026264 | 1 | yes | up | 0.08 | 0.1 | 0.65 | 0.41 | 0 | 0 | 0 | 0 |
| ENSMUSG00000086769 | Gm15587 | 0.319286 | -1.64708 | 0.034747 | 1 | yes | down | 0 | 0.04 | 0.21 | 0.05 | 0.15 | 0.12 | 0.16 | 0.33 |
| ENSMUSG00000086949 | Gm13066 | 0.178194 | -2.48848 | 0.049844 | 1 | yes | down | 0.05 | 0 | 0 | 0.36 | 0 | 0.81 | 0.38 | 0.84 |
| ENSMUSG00000087064 | Sap30bpos | 0.297921 | -1.747 | 0.042362 | 1 | yes | down | 0.02 | 0.11 | 0.05 | 0.05 | 0.14 | 0.06 | 0.26 | 0.15 |
| ENSMUSG00000087142 | Gm12454 | 0.132254 | -2.91862 | 0.017407 | 1 | yes | down | 0 | 0.06 | 0.28 | 0 | 0.34 | 0.54 | 0.67 | 0.12 |
| ENSMUSG00000087194 | Skint6 | 0.027792 | -5.16917 | 0.000287 | 1 | yes | down | 0 | 0 | 0 | 0 | 11.77 | 9.88 | 1.41 | 3.31 |
| ENSMUSG00000087354 | 4930404I05Rik | 0.429795 | -1.21828 | 0.035567 | 0.413092 | yes | down | 0.28 | 0.21 | 0.08 | 0.47 | 0.59 | 0.93 | 0.3 | 0.25 |
| ENSMUSG00000087361 | 0610043K17Rik | 2.878367 | 1.525251 | 0.036527 | 1 | yes | up | 1.12 | 4.95 | 3.89 | 3.21 | 1.36 | 0.64 | 0.64 | 1.39 |
| ENSMUSG00000087685 | 1700122E12Rik | 2.243659 | 1.165854 | 0.02257 | 0.364963 | yes | up | 3.04 | 2.35 | 4.27 | 4.43 | 1.46 | 2.14 | 0.88 | 1.13 |
| ENSMUSG00000088185 | Scarna2 | 0.251647 | -1.99053 | 0.003288 | 0.226644 | yes | down | 0.23 | 1.06 | 1.46 | 0.85 | 2.82 | 4.2 | 2.84 | 2.05 |
| ENSMUSG00000089943 | Ugt1a5 | 0.459351 | -1.12233 | 0.00816 | 0.276708 | yes | down | 1.72 | 0.52 | 0.67 | 1.2 | 1.4 | 1.83 | 2.72 | 1.94 |
| ENSMUSG00000090306 | Adh6-ps1 | 2.090666 | 1.063962 | 0.02735 | 0.387344 | yes | up | 0.18 | 1.41 | 1.02 | 1.08 | 0.37 | 0.3 | 0.23 | 0.54 |
| ENSMUSG00000091135 | Lgals1-ps2 | 6.27476 | 2.64956 | 0.019826 | 1 | yes | up | 0.64 | 2.15 | 0.62 | 1.47 | 0.49 | 0 | 0 | 0.24 |
| ENSMUSG00000093732 | Gm20609 | 10.87535 | 3.44299 | 0.040425 | 1 | yes | up | 0 | 0.26 | 0.13 | 0.19 | 0 | 0.03 | 0 | 0 |
| ENSMUSG00000093846 | Gm4425 | 0.059938 | -4.06038 | 0.014165 | 1 | yes | down | 0 | 0 | 0 | 0 | 0.04 | 0.05 | 0.07 | 0.03 |
| ENSMUSG00000094690 | 1600014C23Rik | 17.0866 | 4.094794 | 0.025098 | 1 | yes | up | 0 | 0.85 | 1.13 | 0.54 | 0 | 0 | 0 | 0 |
| ENSMUSG00000095348 | Gm3892 | 0.137261 | -2.86501 | 0.043979 | 1 | yes | down | 0 | 0.02 | 0.08 | 0.01 | 0 | 0.32 | 0.12 | 0.13 |
| ENSMUSG00000095975 | Cphx1 | 0.12497 | -3.00035 | 0.003617 | 1 | yes | down | 0 | 0.07 | 0.04 | 0.04 | 0.42 | 0.19 | 0.21 | 0.13 |
| ENSMUSG00000096521 | Gm13137 | 2.068267 | 1.048422 | 0.008175 | 0.276708 | yes | up | 2.3 | 2.09 | 2.18 | 2.57 | 1.29 | 1.5 | 0.45 | 0.73 |
| ENSMUSG00000096569 | Amy2a2 | 0.074281 | -3.75086 | 0.036713 | 1 | yes | down | 0.01 | 0.02 | 0 | 0.01 | 0.01 | 0.03 | 0.01 | 0.14 |
| ENSMUSG00000096770 | Amy2a4 | 0.074281 | -3.75086 | 0.036713 | 1 | yes | down | 0.01 | 0.02 | 0 | 0.01 | 0.01 | 0.03 | 0.01 | 0.14 |
| ENSMUSG00000096993 | Gm26787 | 17.19272 | 4.103726 | 0.008741 | 1 | yes | up | 0.33 | 0.54 | 0.57 | 0.38 | 0 | 0 | 0 | 0 |
| ENSMUSG00000097155 | Gm26511 | 0.371607 | -1.42815 | 0.026454 | 0.382064 | yes | down | 0.05 | 0.2 | 0.16 | 0.09 | 0.3 | 0.3 | 0.31 | 0.22 |
| ENSMUSG00000097643 | A130051J06Rik | 2.426184 | 1.278689 | 0.004437 | 0.242414 | yes | up | 0.39 | 0.47 | 0.5 | 0.38 | 0.15 | 0.16 | 0.16 | 0.16 |
| ENSMUSG00000097651 | 4930461G14Rik | 3.794331 | 1.923846 | 0.046274 | 1 | yes | up | 1.15 | 0.39 | 0.44 | 0.41 | 0.41 | 0.15 | 0.07 | 0 |
| ENSMUSG00000097779 | 4833407H14Rik | 14.54857 | 3.862805 | 0.022262 | 1 | yes | up | 0.1 | 0.15 | 0.04 | 0.16 | 0 | 0 | 0 | 0 |
| ENSMUSG00000097825 | 9630001P10Rik | 4.103447 | 2.036836 | 0.024828 | 1 | yes | up | 0.16 | 0.11 | 0.21 | 0.12 | 0.04 | 0 | 0.07 | 0.02 |
| ENSMUSG00000097854 | Gm26602 | 3.975621 | 1.99118 | 0.030848 | 0.399757 | yes | up | 4.61 | 5.7 | 15.33 | 9.3 | 0 | 2.09 | 5.08 | 0 |
| ENSMUSG00000097882 | 0610038B21Rik | 0.217757 | -2.19921 | 0.002685 | 0.219417 | yes | down | 0.06 | 0.03 | 0.19 | 0.15 | 0.29 | 0.25 | 0.62 | 0.47 |
| ENSMUSG00000098196 | Gm26964 | 6.296852 | 2.654631 | 0.009932 | 0.284938 | yes | up | 0 | 2.6 | 1.95 | 1.51 | 0.26 | 0 | 0.05 | 0.47 |
| ENSMUSG00000098302 | Gm28039 | 3.114162 | 1.638844 | 6.73E-05 | 0.031144 | yes | up | 1.58 | 5.6 | 2.11 | 1.48 | 0.33 | 3.08 | 0.22 | 0.7 |
| ENSMUSG00000099764 | Rps10-ps2 | 0.207727 | -2.26724 | 0.028879 | 0.390726 | yes | down | 0.2 | 0 | 0 | 2.38 | 5.02 | 3.41 | 0.56 | 1.85 |
| ENSMUSG00000100210 | H3c7 | 0.18678 | -2.42059 | 0.045311 | 1 | yes | down | 0 | 0.06 | 0 | 0.12 | 0.34 | 0.09 | 0.13 | 0.31 |
| ENSMUSG00000100774 | Gm7329 | 21.98059 | 4.458158 | 0.031044 | 1 | yes | up | 0 | 28.37 | 0 | 13.67 | 0 | 0 | 0 | 0 |
| ENSMUSG00000101163 | Gm13278 | 0.040916 | -4.61119 | 0.025899 | 1 | yes | down | 0 | 0 | 0 | 0 | 0 | 0 | 0.62 | 1.53 |
| ENSMUSG00000101356 | Gm28876 | 0.30525 | -1.71194 | 0.046798 | 1 | yes | down | 0.09 | 0.1 | 0.23 | 0.65 | 0.54 | 0.48 | 0.73 | 1.08 |
| ENSMUSG00000102073 | Gm6818 | 0.163089 | -2.61627 | 0.03761 | 1 | yes | down | 0.06 | 0 | 0 | 0.02 | 0.18 | 0.22 | 0.16 | 0 |
| ENSMUSG00000102448 | Gm37101 | 0.109474 | -3.19134 | 0.044447 | 1 | yes | down | 0 | 0 | 0.02 | 0 | 0.04 | 0.03 | 0.03 | 0.04 |
| ENSMUSG00000103081 | Pcdhgb8 | 2.219573 | 1.150282 | 0.017088 | 0.3351 | yes | up | 0.22 | 0.25 | 0.25 | 0.32 | 0.2 | 0.05 | 0.05 | 0.11 |
| ENSMUSG00000103272 | Gm37914 | 7.114414 | 2.830745 | 0.028712 | 1 | yes | up | 0.03 | 0.16 | 0.13 | 0.14 | 0 | 0.06 | 0 | 0 |
| ENSMUSG00000103739 | Gm37653 | 13.49222 | 3.754056 | 0.035822 | 1 | yes | up | 0.01 | 0.06 | 0.08 | 0.01 | 0 | 0 | 0 | 0 |
| ENSMUSG00000103768 | Gm37856 | 0.118434 | -3.07785 | 0.047326 | 1 | yes | down | 0 | 0.06 | 0.07 | 0 | 0.46 | 0 | 0.22 | 0.24 |
| ENSMUSG00000103847 | Gm20056 | 0.430399 | -1.21625 | 0.017447 | 0.335544 | yes | down | 1.89 | 1.2 | 1.15 | 1.65 | 3.27 | 3.51 | 3.81 | 1.48 |
| ENSMUSG00000103967 | Gm38214 | 16.58938 | 4.052188 | 0.0369 | 1 | yes | up | 0.22 | 0.06 | 0.57 | 0 | 0 | 0 | 0 | 0 |
| ENSMUSG00000104493 | Gm19552 | 13.89843 | 3.79685 | 0.007603 | 1 | yes | up | 0.05 | 0.15 | 0.06 | 0.1 | 0 | 0.02 | 0 | 0 |
| ENSMUSG00000104605 | Gm42922 | 0.200358 | -2.31935 | 0.048504 | 1 | yes | down | 0.02 | 0.05 | 0.03 | 0 | 0.17 | 0.09 | 0.02 | 0.17 |
| ENSMUSG00000105219 | Gm43821 | 2.064801 | 1.046003 | 0.003027 | 0.219417 | yes | up | 1.32 | 4.44 | 4.27 | 4.21 | 1.06 | 1.4 | 0.69 | 2.52 |
| ENSMUSG00000105265 | Sox2ot | 0.233225 | -2.1002 | 0.042626 | 1 | yes | down | 0.08 | 0.13 | 0.02 | 0.13 | 0.12 | 0.11 | 0.22 | 0.56 |
| ENSMUSG00000105362 | Gm43474 | 14.66565 | 3.874369 | 0.01955 | 1 | yes | up | 0.27 | 0.34 | 0.18 | 0.16 | 0 | 0 | 0 | 0 |
| ENSMUSG00000105746 | Gm43595 | 0.082925 | -3.59205 | 0.016753 | 1 | yes | down | 0 | 0.06 | 0 | 0 | 0.12 | 0.16 | 0.21 | 0.22 |
| ENSMUSG00000105925 | Gm43846 | 0.247265 | -2.01587 | 0.043036 | 1 | yes | down | 0.06 | 0 | 0.15 | 0.22 | 0.22 | 0.45 | 0.43 | 0.33 |
| ENSMUSG00000105944 | Gm5075 | 0.055361 | -4.17498 | 0.012454 | 1 | yes | down | 0 | 0 | 0 | 0 | 0.13 | 0.59 | 0.44 | 0.24 |
| ENSMUSG00000106123 | Gm42638 | 0.090035 | -3.47337 | 0.025903 | 1 | yes | down | 0.02 | 0 | 0 | 0 | 0.09 | 0.02 | 0.07 | 0.06 |
| ENSMUSG00000106229 | Gm19409 | 0.054452 | -4.19887 | 0.022412 | 1 | yes | down | 0 | 0 | 0 | 0 | 0.1 | 0 | 0.07 | 0.05 |
| ENSMUSG00000107037 | Gm29793 | 3.204593 | 1.680141 | 0.015799 | 0.328015 | yes | up | 1.46 | 3.82 | 3.29 | 1.36 | 0.9 | 0.79 | 0.57 | 0.41 |
| ENSMUSG00000107222 | Gm43198 | 0.046806 | -4.41717 | 0.036106 | 1 | yes | down | 0 | 0 | 0 | 0 | 0 | 2.75 | 3.3 | 0.19 |
| ENSMUSG00000107633 | Gm44078 | 11.50371 | 3.524027 | 0.023903 | 1 | yes | up | 0.03 | 0.18 | 0.02 | 0.08 | 0 | 0.02 | 0 | 0 |
| ENSMUSG00000107719 | Gm43937 | 0.098963 | -3.33696 | 0.031206 | 1 | yes | down | 0 | 0 | 0.02 | 0 | 0.03 | 0.05 | 0.04 | 0.05 |
| ENSMUSG00000107870 | Gm43940 | 7.69676 | 2.944251 | 0.013943 | 1 | yes | up | 28.54 | 22.04 | 43.65 | 70.53 | 0 | 10.45 | 4.23 | 0 |
| ENSMUSG00000107951 | Gm6210 | 0.091214 | -3.4546 | 0.024416 | 1 | yes | down | 0.03 | 0 | 0 | 0 | 0.12 | 0.06 | 0.06 | 0.1 |
| ENSMUSG00000108120 | 9930120I10Rik | 0.213852 | -2.22532 | 0.032455 | 1 | yes | down | 0.26 | 0 | 0.17 | 0 | 0.8 | 0.29 | 0.54 | 0.29 |
| ENSMUSG00000108353 | Gm45205 | 0.474928 | -1.07422 | 0.006901 | 0.264806 | yes | down | 0.18 | 0.21 | 0.27 | 0.12 | 0.39 | 0.37 | 0.32 | 0.39 |
| ENSMUSG00000108476 | Gm44974 | 7.196486 | 2.847293 | 0.048728 | 1 | yes | up | 0.2 | 0.14 | 0.26 | 0.15 | 0 | 0 | 0 | 0.09 |
| ENSMUSG00000109167 | Gm44652 | 2.309918 | 1.207841 | 0.033203 | 0.405719 | yes | up | 0.13 | 0.22 | 0.45 | 0.36 | 0.11 | 0.1 | 0.1 | 0.1 |
| ENSMUSG00000109644 | 0610005C13Rik | 0.418576 | -1.25644 | 0.002008 | 0.199337 | yes | down | 0.95 | 0.75 | 0.58 | 0.88 | 2.35 | 1.48 | 2.4 | 2.98 |
| ENSMUSG00000109663 | Gm5331 | 0.069153 | -3.85406 | 0.021311 | 1 | yes | down | 0 | 0 | 0 | 0.28 | 0 | 0.49 | 1.18 | 2.13 |
| ENSMUSG00000109857 | Gm53058 | 0.242328 | -2.04496 | 0.022046 | 1 | yes | down | 0.15 | 0.21 | 0 | 0.05 | 0.48 | 0.22 | 0.18 | 0.1 |
| ENSMUSG00000110136 | Gm45785 | 29.50394 | 4.882836 | 0.002993 | 1 | yes | up | 0.56 | 0.27 | 0 | 0.67 | 0 | 0 | 0 | 0 |
| ENSMUSG00000110353 | Gm33543 | 0.076873 | -3.70138 | 0.04312 | 1 | yes | down | 0 | 0 | 0 | 0 | 0.13 | 0.15 | 0.04 | 0.04 |
| ENSMUSG00000110403 | Gm45553 | 2.750036 | 1.45945 | 0.026728 | 0.383429 | yes | up | 0.83 | 2.77 | 3.18 | 2.62 | 0.86 | 1.16 | 0.52 | 0.41 |
| ENSMUSG00000110498 | A630001O12Rik | 2.258057 | 1.175082 | 0.041061 | 0.429508 | yes | up | 0.23 | 0.46 | 0.28 | 0.41 | 0.15 | 0.07 | 0.18 | 0.12 |
| ENSMUSG00000110838 | Gm47789 | 14.35988 | 3.843972 | 0.045262 | 1 | yes | up | 0 | 1.15 | 0.51 | 1.22 | 0 | 0 | 0 | 0 |
| ENSMUSG00000111712 | Gm39363 | 0.492782 | -1.02098 | 0.03955 | 0.42442 | yes | down | 0.78 | 0.88 | 0.99 | 0.52 | 1.38 | 1.7 | 1.23 | 1.23 |
| ENSMUSG00000111994 | A330049N07Rik | 0.460888 | -1.11751 | 0.039544 | 0.42442 | yes | down | 0.39 | 0.14 | 0.21 | 0.21 | 0.47 | 0.45 | 0.6 | 0.29 |
| ENSMUSG00000112121 | C230072F16Rik | 0.426773 | -1.22846 | 0.012693 | 0.304833 | yes | down | 0.27 | 0.51 | 0.34 | 0.47 | 1.33 | 0.64 | 0.7 | 0.52 |
| ENSMUSG00000112265 | Gm34983 | 3.163368 | 1.661461 | 0.032217 | 0.404137 | yes | up | 0.64 | 4.8 | 5.45 | 4.14 | 0.97 | 1.16 | 0 | 1.76 |
| ENSMUSG00000112545 | 1300014J16Rik | 0.474105 | -1.07672 | 0.009485 | 0.282598 | yes | down | 0.67 | 1.08 | 0.54 | 0.59 | 1.91 | 1.43 | 1.44 | 0.58 |
| ENSMUSG00000112652 | 4921516A02Rik | 0.387758 | -1.36677 | 0.009227 | 0.280171 | yes | down | 0.14 | 0.3 | 0.35 | 0.07 | 0.24 | 0.32 | 0.4 | 0.84 |
| ENSMUSG00000113035 | 5830428M24Rik | 0.181201 | -2.46434 | 0.045445 | 1 | yes | down | 0.05 | 0.1 | 0.07 | 0 | 0.43 | 0.19 | 0.27 | 0.23 |
| ENSMUSG00000113334 | D030007L05Rik | 0.055345 | -4.17541 | 0.029572 | 1 | yes | down | 0 | 0 | 0 | 0 | 0 | 0.16 | 0.13 | 0.03 |
| ENSMUSG00000113432 | 8430406P12Rik | 3.821086 | 1.933983 | 0.037659 | 1 | yes | up | 0.25 | 0.74 | 0.45 | 0.78 | 0.36 | 0 | 0 | 0.16 |
| ENSMUSG00000113650 | Gm47826 | 7.896501 | 2.981214 | 0.03737 | 1 | yes | up | 0.07 | 0.11 | 0.04 | 0.07 | 0 | 0 | 0.03 | 0 |
| ENSMUSG00000114091 | Gm40655 | 0.062543 | -3.999 | 0.047258 | 1 | yes | down | 0 | 0 | 0 | 0 | 0.03 | 0 | 0.05 | 0.19 |
| ENSMUSG00000114217 | Gm7054 | 0.10025 | -3.31833 | 0.036382 | 1 | yes | down | 0.08 | 0 | 0 | 0 | 0.29 | 0.25 | 0.08 | 0.34 |
| ENSMUSG00000114354 | Gm34961 | 2.442209 | 1.288187 | 0.000786 | 0.15404 | yes | up | 1.1 | 6.1 | 4.47 | 5.38 | 1.63 | 2.17 | 0.73 | 1.68 |
| ENSMUSG00000114378 | Gm49355 | 23.12335 | 4.531279 | 0.027855 | 1 | yes | up | 0.17 | 0 | 0.11 | 0 | 0 | 0 | 0 | 0 |
| ENSMUSG00000114419 | 5430414B19Rik | 12.22137 | 3.611334 | 0.0409 | 1 | yes | up | 0.08 | 0.28 | 0.31 | 0.29 | 0 | 0 | 0 | 0 |
| ENSMUSG00000114709 | Gm47920 | 4.406663 | 2.139687 | 0.039606 | 1 | yes | up | 0.26 | 0.27 | 0.09 | 0.28 | 0.08 | 0.07 | 0.03 | 0 |
| ENSMUSG00000114942 | Gm49361 | 19.31392 | 4.271569 | 0.016765 | 1 | yes | up | 0.06 | 0.12 | 0.06 | 0 | 0 | 0 | 0 | 0 |
| ENSMUSG00000114967 | Gm7473 | 2.177809 | 1.122877 | 0.02451 | 0.374585 | yes | up | 0.62 | 1.99 | 2.21 | 3.64 | 1.11 | 0.29 | 0.59 | 1.23 |
| ENSMUSG00000115087 | Gm48998 | 0.082934 | -3.59189 | 0.018969 | 1 | yes | down | 0 | 0.02 | 0 | 0 | 0.06 | 0.04 | 0.04 | 0.11 |
| ENSMUSG00000115389 | Gm48936 | 0.322655 | -1.63194 | 0.015141 | 0.322514 | yes | down | 0.06 | 0.09 | 0.15 | 0.09 | 0.34 | 0.08 | 0.27 | 0.29 |
| ENSMUSG00000115422 | 4930452G13Rik | 0.026748 | -5.22442 | 0.000121 | 1 | yes | down | 0 | 0 | 0 | 0 | 0.21 | 0.11 | 0.14 | 0.05 |
| ENSMUSG00000115988 | Gm49441 | 0.188968 | -2.40379 | 0.01207 | 1 | yes | down | 0.28 | 0.13 | 0 | 0.02 | 0.52 | 0.41 | 0.8 | 0.26 |
| ENSMUSG00000116016 | Gm49496 | 0.050327 | -4.31253 | 0.044292 | 1 | yes | down | 0 | 0 | 0 | 0 | 0 | 0.24 | 0 | 0.41 |
| ENSMUSG00000116207 | Nnt | 111.219 | 6.79726 | 0.04475 | 0.438119 | yes | up | 0 | 0.35 | 0 | 1.13 | 0 | 0 | 0 | 0 |
| ENSMUSG00000116287 | Gm3924 | 3.564261 | 1.833603 | 0.016043 | 1 | yes | up | 1.25 | 2.28 | 3.43 | 1.32 | 0.66 | 0.39 | 0.37 | 0.4 |
| ENSMUSG00000116445 | Gm49481 | 9.07475 | 3.181858 | 0.047544 | 1 | yes | up | 0.24 | 0.05 | 0.12 | 0.21 | 0 | 0.05 | 0 | 0 |
| ENSMUSG00000116542 | Gm17783 | 9.162718 | 3.195776 | 0.045932 | 1 | yes | up | 0.19 | 0.06 | 0.32 | 0.12 | 0 | 0 | 0.05 | 0 |
| ENSMUSG00000116617 | Gm49767 | 0.306355 | -1.70672 | 0.024031 | 1 | yes | down | 0.04 | 0.03 | 0.1 | 0.02 | 0.17 | 0.12 | 0.14 | 0.07 |
| ENSMUSG00000116625 | Gm8134 | 0.052381 | -4.25482 | 0.025262 | 1 | yes | down | 0 | 0 | 0 | 0 | 0.07 | 0 | 0.26 | 0.08 |
| ENSMUSG00000116743 | Gm49573 | 0.271416 | -1.88142 | 0.019801 | 1 | yes | down | 0.31 | 0.24 | 0 | 0.43 | 0.63 | 0.83 | 0.74 | 0.96 |
| ENSMUSG00000117042 | 2700054A10Rik | 0.441694 | -1.17888 | 0.01991 | 0.34892 | yes | down | 0.04 | 0.1 | 0.08 | 0.14 | 0.2 | 0.16 | 0.14 | 0.18 |
| ENSMUSG00000117234 | Gm7818 | 2.366403 | 1.242696 | 0.009266 | 0.280171 | yes | up | 0.4 | 1.62 | 1.53 | 1.48 | 0.21 | 0.53 | 0.34 | 0.65 |
| ENSMUSG00000117798 | Gm50164 | 5.1122 | 2.353944 | 0.008745 | 1 | yes | up | 0.16 | 0.21 | 0.18 | 0.13 | 0.04 | 0.02 | 0 | 0.06 |
| ENSMUSG00000117949 | Gm50180 | 5.622699 | 2.491263 | 0.044598 | 1 | yes | up | 4.55 | 4.24 | 5.42 | 4.52 | 2.49 | 0.95 | 0 | 0 |
| ENSMUSG00000117992 | Gm41760 | 9.613012 | 3.264989 | 0.027216 | 1 | yes | up | 0 | 0.18 | 0.15 | 0.19 | 0 | 0 | 0.02 | 0.02 |
| ENSMUSG00000118013 | Gm31706 | 2.024341 | 1.017452 | 0.043106 | 0.435723 | yes | up | 0.12 | 0.32 | 0.35 | 0.44 | 0.13 | 0.08 | 0.13 | 0.17 |
| ENSMUSG00000118607 | Gm7592 | 4.57096 | 2.192497 | 0.004298 | 1 | yes | up | 0.17 | 0.26 | 0.15 | 0.27 | 0.09 | 0.03 | 0.03 | 0.02 |
| ENSMUSG00000118922 | n-R5s103 | 0.052565 | -4.24975 | 0.021344 | 1 | yes | down | 0 | 0 | 0 | 0 | 270.56 | 68.76 | 0 | 150.4 |
| MSTRG.11991 | 0.41313 | -1.27533 | 0.000107 | 0.046755 | yes | down | 3.4 | 1.16 | 2.12 | 2.1 | 6.73 | 4.82 | 4.98 | 2.94 |  |
| MSTRG.12374 | 3.665324 | 1.873941 | 0.019532 | 0.346302 | yes | up | 1.59 | 1.36 | 0.82 | 1.43 | 0.24 | 0.21 | 0.82 | 0 |  |
| MSTRG.1249 | 0.159218 | -2.65093 | 0.008189 | 0.276708 | yes | down | 0.14 | 0.08 | 0 | 0 | 0.12 | 0.14 | 0.7 | 0.22 |  |
| MSTRG.13939 | 0.278806 | -1.84267 | 0.001308 | 0.177309 | yes | down | 8.45 | 2.12 | 2.72 | 1.14 | 14.96 | 18.81 | 11.62 | 5.02 |  |
| MSTRG.14019 | 0.440717 | -1.18208 | 0.006287 | 0.263612 | yes | down | 19.91 | 8.8 | 9.6 | 11.88 | 26.07 | 19.11 | 33.67 | 21.1 |  |
| MSTRG.14428 | 0.439002 | -1.1877 | 0.001392 | 0.177309 | yes | down | 0.9 | 1.14 | 1.23 | 0.67 | 2.72 | 2.69 | 1.48 | 1.04 |  |
| MSTRG.14823 | 0.384622 | -1.37849 | 0.049949 | 0.447273 | yes | down | 1.01 | 0.27 | 1.58 | 0.24 | 2.73 | 1.6 | 1.72 | 1.06 |  |
| MSTRG.15308 | 2.647399 | 1.404576 | 0.010832 | 0.291625 | yes | up | 0.18 | 1.99 | 2.05 | 4.81 | 0.51 | 0.23 | 0.45 | 0.66 |  |
| MSTRG.16388 | 0.379672 | -1.39717 | 0.007592 | 0.269607 | yes | down | 0.52 | 1.95 | 1.51 | 0.96 | 3.81 | 3.8 | 2.29 | 1.31 |  |
| MSTRG.16440 | 2.400414 | 1.263283 | 0.029191 | 0.393342 | yes | up | 1.18 | 1.14 | 1.66 | 2.42 | 0.84 | 0.64 | 0.51 | 0.33 |  |
| MSTRG.16554 | 2.044199 | 1.031536 | 0.001029 | 0.172068 | yes | up | 11.91 | 28.5 | 30.23 | 26.44 | 15.93 | 9.12 | 3.91 | 12.37 |  |
| MSTRG.20933 | 5.044328 | 2.334662 | 0.047289 | 1 | yes | up | 2.84 | 6.62 | 4.68 | 0.59 | 2.35 | 0.5 | 0 | 0 |  |
| MSTRG.20948 | 0.414838 | -1.26938 | 0.045999 | 0.440788 | yes | down | 4.31 | 0.95 | 2.8 | 1.15 | 3.97 | 7.82 | 6.3 | 2.26 |  |
| MSTRG.21546 | 0.483013 | -1.04987 | 0.006838 | 0.26411 | yes | down | 1.94 | 0.94 | 1.12 | 1.39 | 3.19 | 2.13 | 3.22 | 1.41 |  |
| MSTRG.21737 | 2.007864 | 1.005662 | 0.03299 | 0.4049 | yes | up | 1 | 3.89 | 2.7 | 5.74 | 2.56 | 1.19 | 1.36 | 0.6 |  |
| MSTRG.21751 | 2.935055 | 1.553387 | 0.019522 | 0.346302 | yes | up | 1.75 | 7.48 | 5.72 | 5.43 | 1.66 | 1.24 | 2.77 | 0 |  |
| MSTRG.22101 | 0.155107 | -2.68867 | 0.003939 | 0.240678 | yes | down | 0.54 | 0.06 | 0 | 0.07 | 0.63 | 0.93 | 2.11 | 0.34 |  |
| MSTRG.251 | 2.623694 | 1.3916 | 0.000369 | 0.097642 | yes | up | 4.7 | 11.18 | 12.79 | 11.83 | 1.35 | 3.63 | 3.13 | 4.48 |  |
| MSTRG.2580 | 2.523132 | 1.335215 | 0.01048 | 0.289463 | yes | up | 1.11 | 1.6 | 1.38 | 1.94 | 1.09 | 0.23 | 0.44 | 0.46 |  |
| MSTRG.4882 | 2.785611 | 1.477994 | 0.02203 | 0.361135 | yes | up | 0.3 | 1.52 | 2.11 | 1.24 | 0.66 | 0.2 | 0.19 | 0.53 |  |
| MSTRG.4893 | 0.242644 | -2.04309 | 0.001809 | 0.190898 | yes | down | 2.62 | 0.29 | 0.64 | 0.62 | 3.77 | 4.3 | 5.96 | 2.21 |  |
| MSTRG.6822 | 5.948143 | 2.572439 | 0.026852 | 1 | yes | up | 0.46 | 1.33 | 3.78 | 1.7 | 0.56 | 0 | 0 | 0.51 |  |
| MSTRG.6986 | 0.075435 | -3.72862 | 0.034747 | 1 | yes | down | 0.12 | 0 | 0 | 0 | 0.17 | 0.3 | 1.09 | 0 |  |
| MSTRG.8113 | 0.32836 | -1.60665 | 0.000165 | 0.062337 | yes | down | 14.65 | 6.4 | 6.86 | 3.82 | 30.74 | 26.32 | 21.7 | 11.51 |  |
| MSTRG.9535 | 2.740842 | 1.454619 | 0.005923 | 0.260899 | yes | up | 0.54 | 3.86 | 2.09 | 3.07 | 0.28 | 0.51 | 0.57 | 1.33 |  |

Table 6. Interaction protein by ADM promoter region DNA pull-down in hepatocytes

| Gene Symbol | Confidence | Accession | Contaminant | Peptides | PSMs | Unique Peptides | AAs | MW [kDa] | calc. pI | Score |
| --- | --- | --- | --- | --- | --- | --- | --- | --- | --- | --- |
| S1 (-2000~-1482) | | | | | | | | | | |
| Flna | High | Q8BTM8 | TRUE | 1 | 6 | 1 | 2647 | 281 | 6.04 | 18.63 |
| Rpl14 | High | Q9CR57 | TRUE | 1 | 5 | 1 | 217 | 23.5 | 11.02 | 5.84 |
| Hnrnpa2b1 | High | O88569 | TRUE | 3 | 4 | 3 | 353 | 37.4 | 8.95 | 5.04 |
| Npm1 | High | Q61937 | TRUE | 2 | 2 | 2 | 292 | 32.5 | 4.77 | 4.38 |
| Pkm | High | P52480 | TRUE | 1 | 1 | 1 | 531 | 57.8 | 7.47 | 4.23 |
| Hnrnpm | High | Q9D0E1 | TRUE | 2 | 2 | 2 | 729 | 77.6 | 8.63 | 3.62 |
| Snu13 | High | Q9D0T1 | TRUE | 1 | 3 | 1 | 128 | 14.2 | 8.46 | 3.56 |
| Psmb4 | High | P99026 | TRUE | 1 | 1 | 1 | 264 | 29.1 | 5.64 | 3.04 |
| Anxa2 | High | P07356 | TRUE | 1 | 1 | 1 | 339 | 38.7 | 7.69 | 2.93 |
| Eftud2 | High | O08810 | TRUE | 1 | 1 | 1 | 971 | 109.3 | 5 | 2.49 |
| Hspa1a | High | Q61696 | TRUE | 1 | 1 | 1 | 641 | 70 | 5.72 | 2.38 |
| Eef1d | High | P57776 | TRUE | 1 | 1 | 1 | 281 | 31.3 | 5.02 | 2.34 |
| Ywhaz | High | P63101 | TRUE | 1 | 1 | 1 | 245 | 27.8 | 4.79 | 1.91 |
| Ddx17 | High | Q501J6 | TRUE | 1 | 1 | 1 | 650 | 72.4 | 8.59 | 1.85 |
| Nfib | High | P97863 | TRUE | 1 | 1 | 1 | 570 | 63.5 | 8.66 | 1.84 |
| Sde2 | High | Q8K1J5 | TRUE | 1 | 1 | 1 | 448 | 48.6 | 5.66 | 1.72 |
| Hmgb1 | High | P63158 | TRUE | 1 | 3 | 1 | 215 | 24.9 | 5.74 | 1.69 |
| Hnrnpu | High | Q8VEK3 | TRUE | 1 | 1 | 1 | 800 | 87.9 | 6.24 | 0 |
| Hnrnpk | High | P61979 | TRUE | 1 | 1 | 1 | 463 | 50.9 | 5.54 | 0 |
| Sap18 | High | O55128 | TRUE | 1 | 1 | 1 | 153 | 17.6 | 9.35 | 0 |
| Rps7 | High | P62082 | TRUE | 1 | 1 | 1 | 194 | 22.1 | 10.1 | 0 |
| Rpl11 | High | Q9CXW4 | TRUE | 1 | 1 | 1 | 178 | 20.2 | 9.6 | 0 |
| Sf3b4 | High | Q8QZY9 | TRUE | 1 | 1 | 1 | 424 | 44.3 | 8.56 | 0 |
| Hcfc1 | High | Q61191 | TRUE | 1 | 1 | 1 | 2045 | 210.3 | 7.18 | 0 |
| Snrnp70 | High | Q62376 | TRUE | 1 | 1 | 1 | 448 | 52 | 9.94 | 0 |
| Sf3a2 | High | Q62203 | TRUE | 1 | 1 | 1 | 475 | 49.9 | 9.54 | 0 |
| S2(-1501~~-920) | | | | | | | | | | |
| Flna | High | Q8BTM8 | TRUE | 1 | 1 | 1 | 2647 | 281 | 6.04 | 3.29 |
| Hnrnpa2b1 | High | O88569 | TRUE | 1 | 1 | 1 | 353 | 37.4 | 8.95 | 2.98 |
| Rpl14 | High | Q9CR57 | TRUE | 1 | 1 | 1 | 217 | 23.5 | 11.02 | 2.46 |
| S3(-940~~-510) | | | | | | | | | | |
| Hnrnpa2b1 | High | O88569 | TRUE | 1 | 5 | 1 | 353 | 37.4 | 8.95 | 12.98 |
| Flna | High | Q8BTM8 | TRUE | 1 | 3 | 1 | 2647 | 281 | 6.04 | 7.78 |
| Hnrnpm | High | Q9D0E1 | TRUE | 2 | 2 | 2 | 729 | 77.6 | 8.63 | 4.59 |
| Phb1 | High | P67778 | TRUE | 1 | 1 | 1 | 272 | 29.8 | 5.76 | 3.11 |
| Rps25 | High | P62852 | TRUE | 1 | 1 | 1 | 125 | 13.7 | 10.11 | 2.5 |
| Glud1 | High | P26443 | TRUE | 1 | 1 | 1 | 558 | 61.3 | 8 | 2.46 |
| Ddx17 | High | Q501J6 | TRUE | 1 | 1 | 1 | 650 | 72.4 | 8.59 | 2.39 |
| Srsf2 | High | Q62093 | TRUE | 1 | 1 | 1 | 221 | 25.5 | 11.85 | 2.25 |
| Luc7l | High | Q9CYI4 | TRUE | 1 | 1 | 1 | 371 | 43.9 | 9.88 | 1.97 |
| S4(-538 ~~ 0) | | | | | | | | | | |
| Hnrnpa2b1 | High | O88569 | TRUE | 5 | 9 | 5 | 353 | 37.4 | 8.95 | 27.24 |
| Rps25 | High | P62852 | TRUE | 1 | 1 | 1 | 125 | 13.7 | 10.11 | 2.6 |
| Glud1 | High | P26443 | TRUE | 1 | 1 | 1 | 558 | 61.3 | 8 | 2.57 |
| Syncrip | High | Q7TMK9 | TRUE | 1 | 1 | 1 | 623 | 69.6 | 8.59 | 2.57 |
| Snu13 | High | Q9D0T1 | TRUE | 1 | 1 | 1 | 128 | 14.2 | 8.46 | 2.24 |
| Srsf1 | High | Q6PDM2 | TRUE | 1 | 1 | 1 | 248 | 27.7 | 10.36 | 1.99 |
| Hmgb1 | High | P63158 | TRUE | 1 | 1 | 1 | 215 | 24.9 | 5.74 | 1.87 |
| Sf3b6 | High | P59708 | TRUE | 1 | 1 | 1 | 125 | 14.6 | 9.38 | 0 |
| Mecp2 | High | Q9Z2D6 | TRUE | 1 | 1 | 1 | 484 | 52.3 | 9.96 | 0 |
| Uba52 | High | P62984 | TRUE | 1 | 1 | 1 | 128 | 14.7 | 9.83 | 0 |
| Hnrnpk | High | P61979 | TRUE | 1 | 1 | 1 | 463 | 50.9 | 5.54 | 0 |
| Nfib | High | P97863 | TRUE | 1 | 1 | 1 | 570 | 63.5 | 8.66 | 0 |

Table 7. Interaction protein by UCP1 promoter region DNA pull-down in HIB1B cells

| Gene Symbol | Confidence | Accession | Contaminant | Peptides | PSMs | Unique Peptides | AAs | MW [kDa] | calc. pI | Score |
| --- | --- | --- | --- | --- | --- | --- | --- | --- | --- | --- |
| S1(-2000~~-1477) | | | | | | | | | | |
| Ddx21 | High | Q9JIK5 | TRUE | 4 | 4 | 4 | 851 | 93.5 | 9.11 | 13.23 |
| Hnrnpm | High | Q9D0E1 | TRUE | 5 | 5 | 5 | 729 | 77.6 | 8.63 | 5.79 |
| Hnrnpab | High | Q99020 | TRUE | 2 | 2 | 2 | 285 | 30.8 | 7.91 | 5.19 |
| Hnrnpa1 | High | P49312 | TRUE | 2 | 2 | 2 | 320 | 34.2 | 9.23 | 4.79 |
| Hspa8 | High | P63017 | TRUE | 1 | 1 | 1 | 646 | 70.8 | 5.52 | 3.74 |
| Cdc5l | High | Q6A068 | TRUE | 2 | 2 | 2 | 802 | 92.1 | 8.02 | 3.31 |
| Nop2 | High | Q922K7 | TRUE | 1 | 1 | 1 | 793 | 86.7 | 9.22 | 3.1 |
| NONO | High | Q99K48 | TRUE | 1 | 1 | 1 | 473 | 54.5 | 8.95 | 2.88 |
| Rpl14 | High | Q9CR57 | TRUE | 1 | 1 | 1 | 217 | 23.5 | 11.02 | 2.85 |
| Krr1 | High | Q8BGA5 | TRUE | 1 | 1 | 1 | 380 | 43.5 | 9.79 | 2.74 |
| Hnrnpu | High | Q8VEK3 | TRUE | 1 | 1 | 1 | 800 | 87.9 | 6.24 | 2.63 |
| Nsun2 | High | Q1HFZ0 | TRUE | 1 | 1 | 1 | 757 | 85.4 | 6.58 | 2.53 |
| Ftsj3 | High | Q9DBE9 | TRUE | 1 | 1 | 1 | 838 | 95.5 | 8.38 | 2.2 |
| Prpf19 | High | Q99KP6 | TRUE | 1 | 1 | 1 | 504 | 55.2 | 6.61 | 2.19 |
| Ddx18 | High | Q8K363 | TRUE | 1 | 1 | 1 | 660 | 74.1 | 9.52 | 2.11 |
| Ddx17 | High | Q501J6 | TRUE | 1 | 1 | 1 | 650 | 72.4 | 8.59 | 2.02 |
| Srpk1 | High | O70551 | TRUE | 1 | 1 | 1 | 648 | 73 | 6.19 | 1.92 |
| Sfpq | High | Q8VIJ6 | TRUE | 1 | 1 | 1 | 699 | 75.4 | 9.44 | 1.84 |
| Gtpbp4 | High | Q99ME9 | TRUE | 1 | 1 | 1 | 634 | 74.1 | 9.52 | 1.74 |
| Rbm17 | High | Q8JZX4 | TRUE | 1 | 1 | 1 | 405 | 45.3 | 5.82 | 0 |
| Ruvbl2 | High | Q9WTM5 | TRUE | 1 | 1 | 1 | 463 | 51.1 | 5.64 | 0 |
| Pabpc1 | High | P29341 | TRUE | 1 | 1 | 1 | 636 | 70.6 | 9.5 | 0 |
| Rps14 | High | P62264 | TRUE | 1 | 1 | 1 | 151 | 16.3 | 10.05 | 0 |
| Hnrnpa2b1 | High | O88569 | TRUE | 2 | 2 | 2 | 353 | 37.4 | 8.95 | 0 |
| Qki | High | Q9QYS9 | TRUE | 1 | 1 | 1 | 341 | 37.6 | 8.56 | 0 |
| Ruvbl1 | High | P60122 | TRUE | 1 | 1 | 1 | 456 | 50.2 | 6.42 | 0 |
| Ddx52 | High | Q8K301 | TRUE | 1 | 1 | 1 | 598 | 67.4 | 9.57 | 0 |
| Hnrnpc | High | Q9Z204 | TRUE | 1 | 1 | 1 | 313 | 34.4 | 5.05 | 0 |
| S2(-1501~~-1104) | | | | | | | | | | |
| Flna | High | Q8BTM8 | TRUE | 1 | 2 | 1 | 2647 | 281 | 6.04 | 7.44 |
| Hnrnpm | High | Q9D0E1 | TRUE | 2 | 2 | 2 | 729 | 77.6 | 8.63 | 4.69 |
| Hnrnpa3 | High | Q8BG05 | TRUE | 1 | 1 | 1 | 379 | 39.6 | 9.01 | 3.57 |
| Hnrnpc | High | Q9Z204 | TRUE | 1 | 1 | 1 | 313 | 34.4 | 5.05 | 2.89 |
| Poldip3 | High | Q8BG81 | TRUE | 1 | 1 | 1 | 420 | 46.1 | 10.05 | 2.67 |
| Rbfox2 | High | Q8BP71 | TRUE | 1 | 1 | 1 | 449 | 47.3 | 6.55 | 2.54 |
| Nup93 | High | Q8BJ71 | TRUE | 1 | 1 | 1 | 819 | 93.2 | 5.72 | 2.52 |
| Utp14a | High | Q640M1 | TRUE | 1 | 1 | 1 | 767 | 87.2 | 9.2 | 2.04 |
| Ddx18 | High | Q8K363 | TRUE | 1 | 1 | 1 | 660 | 74.1 | 9.52 | 2.02 |
| S3(-1126~~-494) | | | | | | | | | | |
| Hnrnpa3 | High | Q8BG05 | TRUE | 1 | 1 | 1 | 379 | 39.6 | 9.01 | 3.28 |
| Krr1 | High | Q8BGA5 | TRUE | 1 | 1 | 1 | 380 | 43.5 | 9.79 | 3.15 |
| Flna | High | Q8BTM8 | TRUE | 1 | 1 | 1 | 2647 | 281 | 6.04 | 2.86 |
| Hnrnpm | High | Q9D0E1 | TRUE | 1 | 1 | 1 | 729 | 77.6 | 8.63 | 2.23 |
| S4(-514~~0) | | | | | | | | | | |
| Hnrnpm | High | Q9D0E1 | TRUE | 3 | 3 | 3 | 729 | 77.6 | 8.63 | 7.1 |
| Flna | High | Q8BTM8 | TRUE | 1 | 1 | 1 | 2647 | 281 | 6.04 | 4.79 |
| Nup93 | High | Q8BJ71 | TRUE | 1 | 1 | 1 | 819 | 93.2 | 5.72 | 2.4 |
| Ddx21 | High | Q9JIK5 | TRUE | 1 | 1 | 1 | 851 | 93.5 | 9.11 | 2.38 |
| Jup | High | Q02257 | TRUE | 1 | 1 | 1 | 745 | 81.7 | 6.14 | 1.93 |

Supporting Information

Supplemental figure


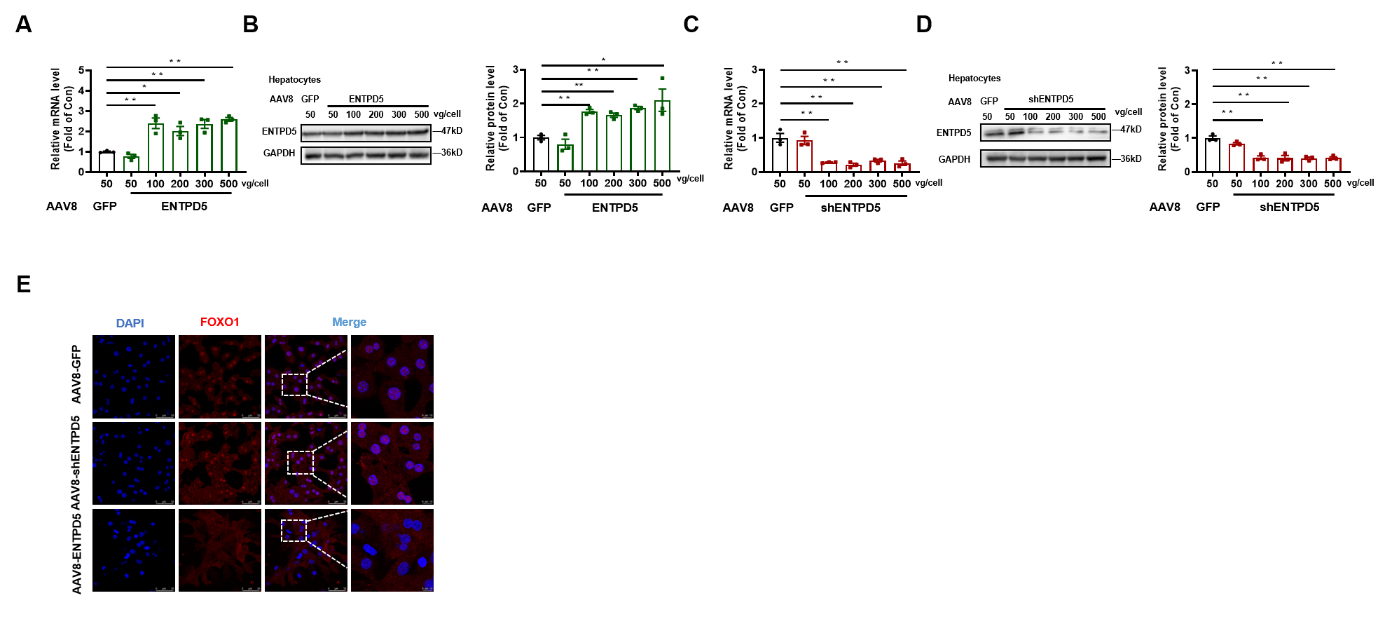


Figure S1. Verification of AAV8-ENTPD5 and AAV8-shENTPD5 in mouse hepatocytes. A-B) Relative mRNA (A) and protein level (B) of ENTPD5 in mouse primary hepatocytes after infection with AAV8-GFP (50 vg/cell) or AAV8-ENTPD5 (50,100, 200, 300, 500 vg/cell) for 36 h (N=3). C-D) Relative mRNA (C) and protein level (D) of ENTPD5 in mouse primary hepatocytes after infection with AAV8-GFP (50 vg/cell) or AAV8-shENTPD5 (50,100, 200, 300, 500 vg/cell) for 36 hours (N=3) E) ENTPD5 overexpression or silencing on the nuclear exclusion of FOXO1 in mouse hepatocytes. (scale bar: 10μm 50μm) Hepatocytes were infected with 100 vg/cell of AAV8-ENTPD, AAV8-shENTPD8 or AAV8-GFP, respectively, for 36 h ***P*<0.01 versus control cells.


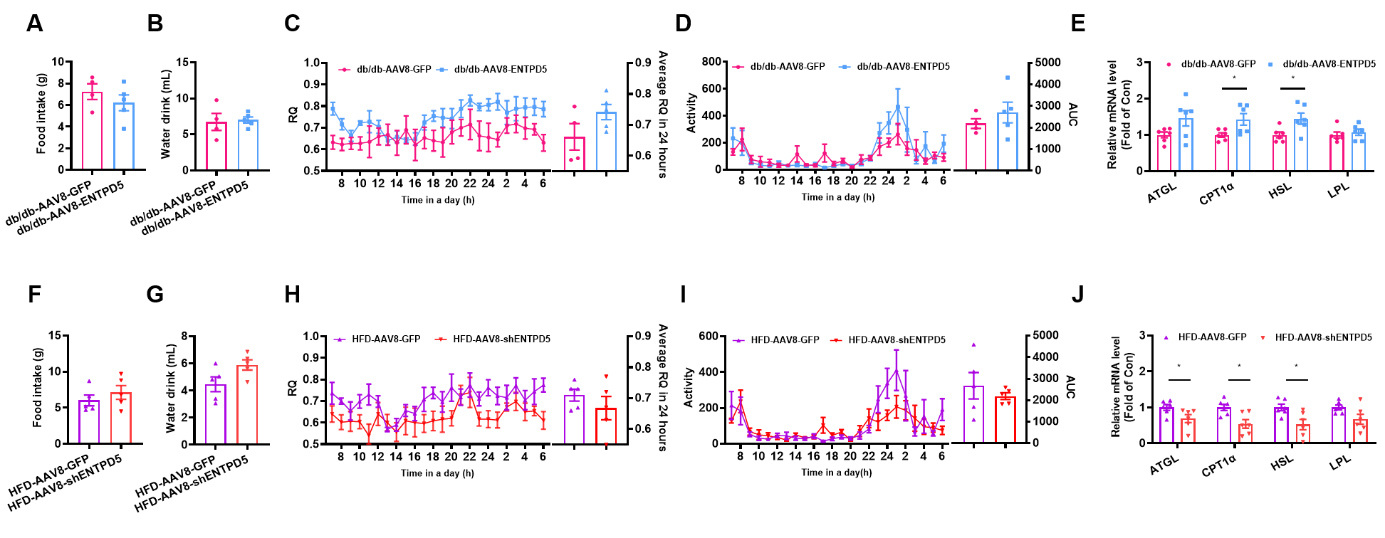


Figure S2. Hepatic modulation of ENTPD5 on food intake and water drink of obese mice. A-D) Hepatic ENTPD5 overexpression had little effect on food intake (A), water drink (B), the curves of respiratory quotient (RQ) (C), and activity (D) of db/db mice as assayed in metabolic cages (N=4-5). E) Relative mRNAs of lipolytic genes in WAT of db/db mice (N=6). F-I) The Influence of hepatic ENTPD5 silencing on food intake, water drink, RQ curve, and activity of HFD-fed mice as assayed in metabolic cages (N=5). J) Relative mRNA of lipolytic genes in WAT of HFD mice (N=6). **P*<0.05 versus control mouse group.


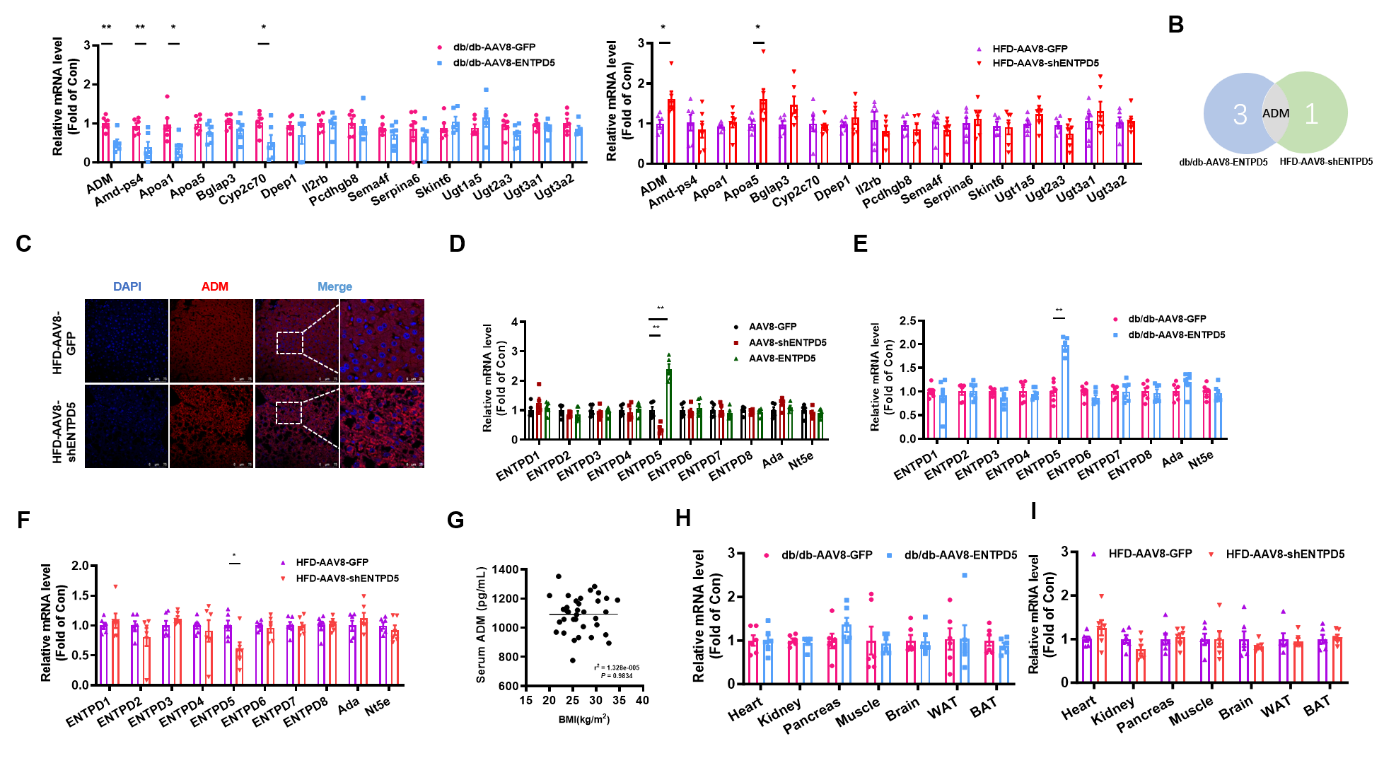


**Figure S3. ENTPD5 overexpression or silencing had little effect on the expressions of other ATP-metabolizing enzymes in hepatocytes and mouse livers**. A) Hepatic overexpression or silencing on the mRNA levels of hepatocyte-secreted protein genes identified from RNA-sequencing hepaocytes in livers of db/db mice with hepatic ENTPD5 overexpression (left) and HFD mice with hepatic ENTPD5 knockdown (right) (N=6).B) Venn chart showed that ADM was the only gene with consistent change in mouse livers with hepatic ENTPD5 overexpression or silencing. C) The confocal images of ADM protein staining in HFD mouse livers with ENTPD5 knockdown. (scale bar: 25μm 75μm). D) The Influence of ENTPD5 overexpression or silencing on the mRNA levels of ATP-hydrolytic enzymes in hepatocytes (N=5). E-F) The Influence of hepatic ENTPD5 overexpression or silencing on the mRNA levels of ATP-hydrolytic enzymes in db/db mouse or HFD mouse livers (N=6). G) The correlation between serum adiponectin levels and BMI in obese adolescents after weight loss (N=35). H-I) The influence of hepatic ENTPD5 overexpression or knockdown on the expression of ADM in other major organs beyond liver (N=6). **P*<0.05, ***P*<0.01versus control cells or mouse group, or between two-indicated groups.


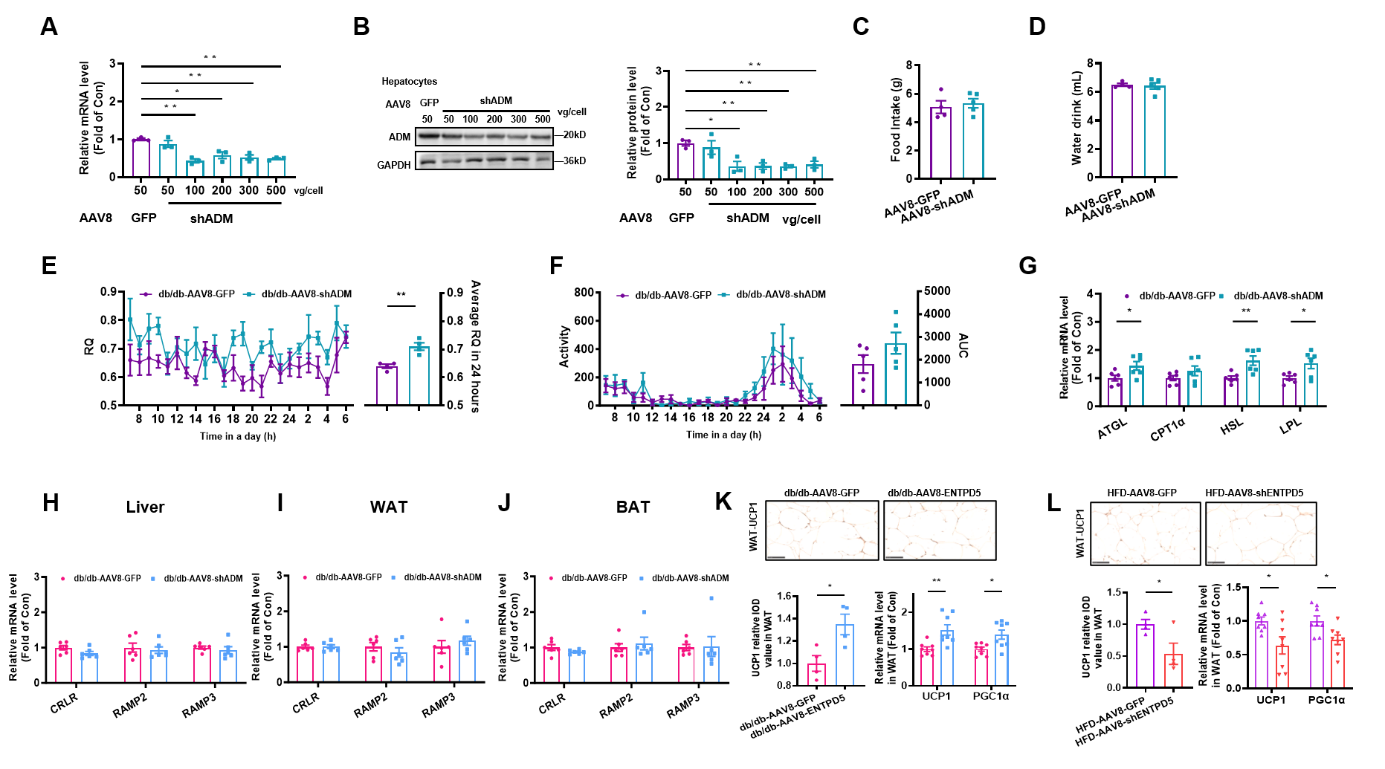


**Figure S4. The Influence of** **hepatic silencing of ADM on food intake and water drink of db/db mice.** A-B) Relative mRNA and protein levels of ADM in mouse primary hepatocytes infected with AAV8-GFP (50vg/cell) or AAV8-shADM (50,100, 200, 300, 500 vg/cell) for 36 hours (N=3 for both WB and mRNA detection). C–F) The influence of hepatic silencing of ADM on food intake (C), water drink (D), RQ (E) and activity (F) of db/db mice as assayed in metabolic cages (N=4). G) Relative mRNAs of lipolytic genes WAT of db/db mice after hepatic ADM knockdown (N=6). (H-J) The influence of hepatic ADM knockdown on mRNA expressions of ADM receptors in liver (H, N=6), WAT (I, N=6) and BAT (J, N=6) of mice. (K) The expression of key thermogenic genes in WAT in db/db mouse with hepatic ENTPD5 overexpression. Immunohistochemical analysis showed the expression of UCP1 in WAT, while bar graphs represented the statistical analysis of grayscale values from the staining (lower left, N=4), and the mRNA expression levels of key thermogenic genes (lower right，N=8) (Scale bar:100μm). (L) The expression of key thermogenic genes in WAT of HFD mice with with hepatic ENTPD5 knockdown. Immunohistochemical analysis showed the expression of UCP1 in WAT, while bar graphs represented the statistical analysis of grayscale values from the staining (lower left, N=4), and the mRNA expression levels of key thermogenic genes (lower right，N=8). (Scale bar:100μm). WAT, white adipose tissue. **P*<0.05, ***P*<0.01versus control cells or mouse group.


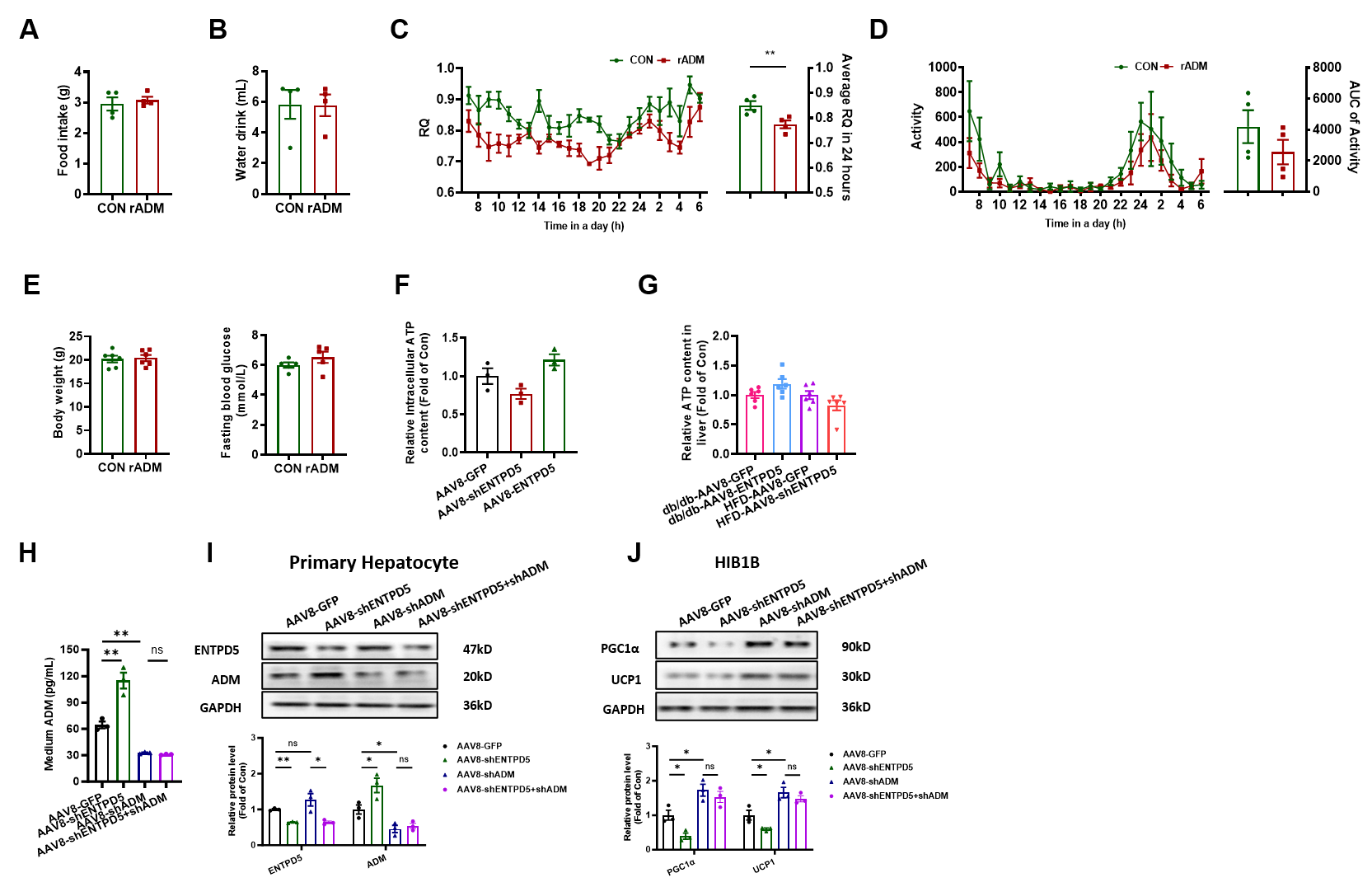


**Figure S5. The influence of rADM treatment on food intake and water drink of C57BL/6 mice** A–E) Treatment with rADM for 1 week on food intake (A), water drink (B), RQ (C), activity (D) assayed in metabolic cages, fasting blood glucose and body weight (E) of normal C57BL/6 mice (N=4-6). F-G) ENTPD5 overexpression or knockdown had little effect on ATP content in cultured hepatocytes (F) (N=3) and obese mouse livers (G) (N=6). rADM, recombinant ADM protein; H-J) ADM protein levels in the medium of hepatocyte-brown adipocyte cell line HIB1B coculture system with ENTPD5 and ADM double knockdown in mouse hepatocytes (H). Protein expression levels of ENTPD5 and ADM in hepatocytes (N=3), and bar graphs illustrated densitometric values from WB (I). Protein expression levels of UCP1in HIB1B cells (N=3), and bar graphs illustrated densitometric values from WB (J). **P*<0.05, ***P*<0.01versus control cells or mouse group.


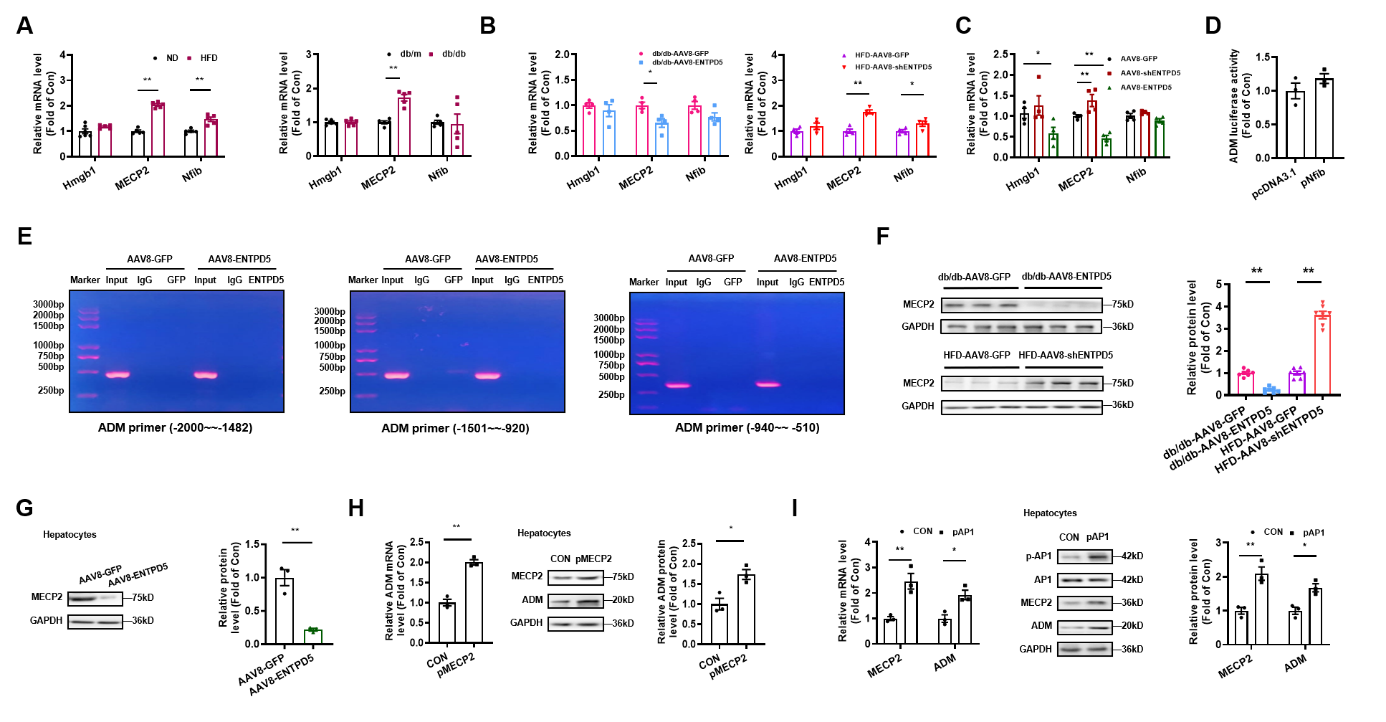


**Figure S6. The influence of ENTPD5 overexpression or silencing on MECP2 expression in hepatocytes and mouse livers.** A) Change in mRNA expressions of Hmgb1, MECP2 and Nifb in db/db and HFD mouse livers (N=5-6). B) Change in mRNA expressions of Hmgb1, MECP2 and Nifb in obese mouse livers with ENTPD5 overexpression or silencing (N=4). C) Change in mRNA expressions of Hmgb1, MECP2 and Nifb in mouse primary hepatocytes with ENTPD5 overexpression or silencing (N=4). D) Luciferase reporter assay revealed that Nifb overexpression failed to affect mouse ADM gene promoter activity in HEK293T cells (N=3). E) ChIP assays failed to identify the binding of MECP2 with the -2000 ~ -1482 region of mouse ADM gene promoter. F) The influence of overexpression or silencing of ENTPD5 on protein level of MECP2 in obese mouse livers (N=7). G) ENTPD5 overexpression reduced the protein expression of MECP2 in mouse hepatocytes (N=3). H) Plasmid overexpression of MECP2 upregulated the expressions of ADM mRNA and proteins in hepatocytes (N=3). I) The influence of MECP2 or ADM overexpression on the mRNA (N=3) and protein expression (N=3) of AP-1 in primary hepatocytes. **P*<0.05, ***P*<0.01versus control cells or mouse group.


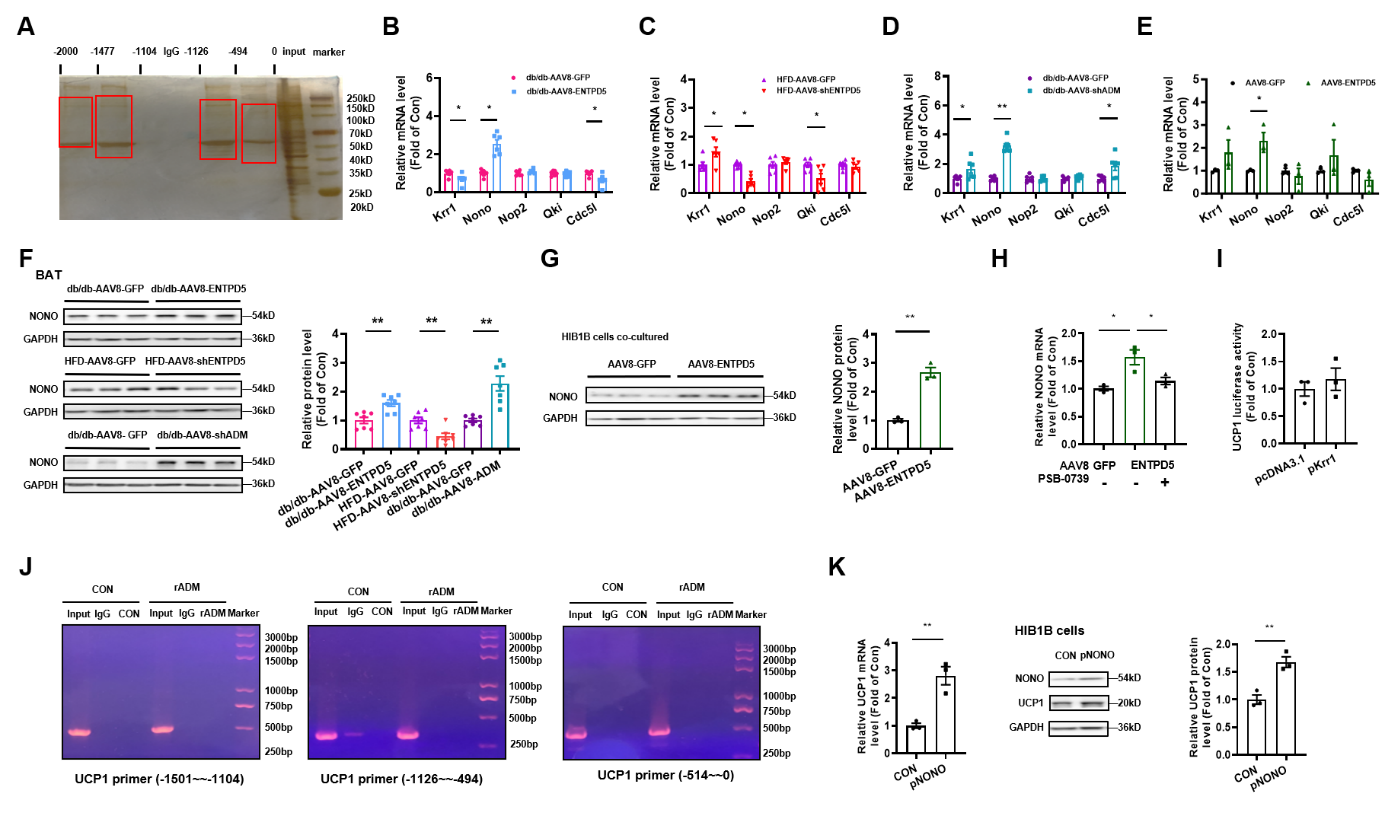


**Figure S7. ADM inhibited UCP1 expression via NONO in HIB1B cells.** A) Representative silver-stained gel image of DNA pull-down products in HIB1B cell. The protein bands in the marked red circles were subjected to mass spectrometry analysis. B-D) Change in mRNAs of Krr1, NONO, Nop2, Qki and Cdc5l in BAT of obese mice with hepatic ENTPD5 overexpression (N=6) or silencing (N=6), or ADM inhibition (N=6). E) Change in mRNAs of Krr1, NONO, Nop2, Qki and Cdc5l in HIB1B cells cocultured with mouse primary hepatocytes treated with AAV8-GFP or AAV8-ENTPD5 (N=3). F) Change in NONO protein level in BAT of obese mice with hepatic ENTPD5 overexpression or silencing, or ADM inhibition (N=7). G) Change in NONO protein level in HIB1B cells cocultured with mouse primary hepatocytes with ENTPD5 overexpression (N=3). H) Change in NONO mRNA in HIB1B cells cocultured with mouse primary hepatocytes with ENTPD5 overexpression in the absence or presence of P2Y_12_ inhibitor PSB-0739 (20μM) (N=3). I) Luciferase reporter assay revealed that Krr1 overexpression had no significant effect on the mouse UCP1 gene promoter activity in HEK293T cells (N=3). J) ChIP assays failed to identify the binding of NONO with the -1501 ~ 0 region of mouse UCP1 gene promoter. K) Plasmid overexpression of NONO increased the expressions of UCP1 mRNA(N=3) and proteins(N=3) in HIB1B cells. **P*<0.05, ***P*<0.01versus control cells or mouse group, or between two-indicated groups.


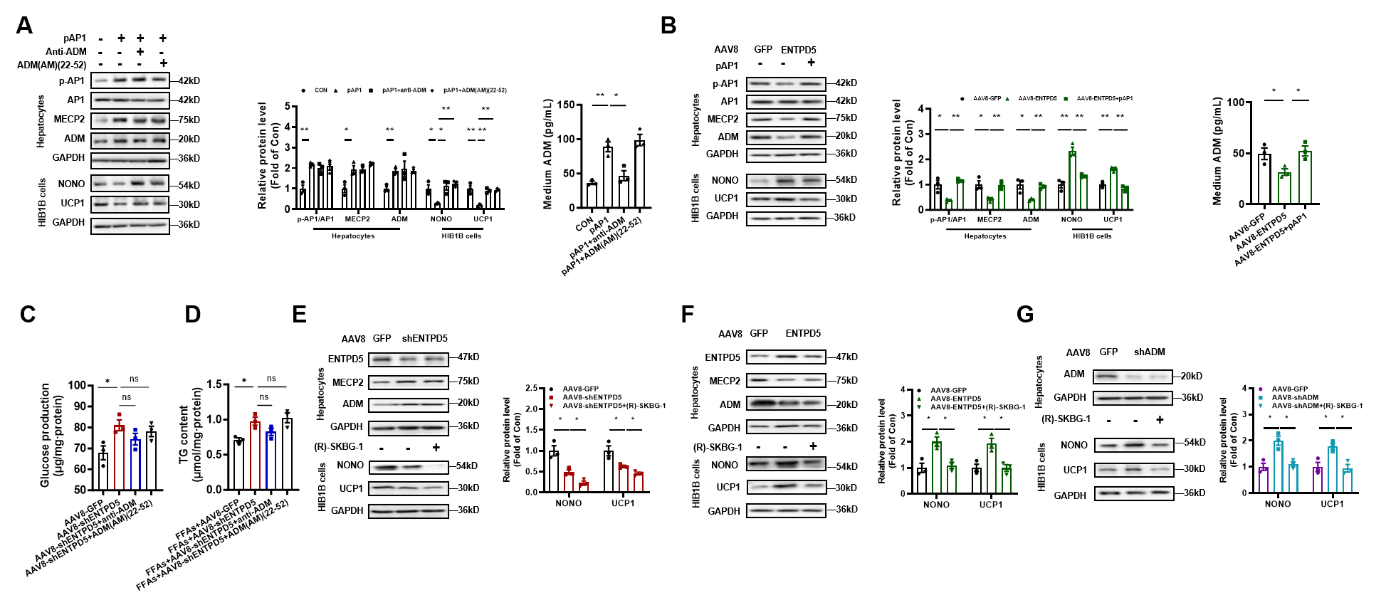


**Figure S8. ENTPD5’s regulatory effects on gluconeogenesis and lipid deposition were not dependent on ADM secretion in hepatocy**tes. A) In hepatocyte-HIB1B coculture system, plasmid overexpression of AP1 in hepatocytes decreased the expressions of NONO and UCP1 proteins in HIB1B cells. Treatment with anti-ADM antibodies or ADM receptor antagonist reversed the inhibition of NONO and UCP1 expressions in HIB1B cells induced by hepatic AP1 overexpression. The influence of anti-ADM antibodies or ADM receptor antagonist on ADM level in the medium of coculture system (N=3). B) In hepatocyte-HIB1B coculture system, hepatic ENDPD5 overexpression reduced ADM expression and secretion in hepatocytes, and decreased the expressions of NONO and UCP1 in HIB1B cells, but were reversed by hepatic AP1 overexpression (N=3). C) Elevation in glucose production induced by ENTPD5 silencing was not affected by incubation with anti-ADM antibodies or antagonist of ADM receptor (ADM(AM)22-52) in mouse hepatocytes (N=3). D) Increase in TG content induced by ENTPD5 inhibition in the presence of FFAs was not affected by incubation with anti-ADM antibodies or antagonist of ADM receptor (N=3). E）Co-culture experiment: effect of ENTPD5 knockdown on MECP2 and ADM protein levels in primary hepatocytes of the upper chamber (N=3), and effect of NONO inhibitor on UCP1 protein expression levels in HIB1B cells co-cultured with primary hepatocytes in the lower chamber (N=3). F) Co-culture experiment: effect of ENTPD5 overexpressionn on MECP2 and ADM protein levels in primary hepatocytes of the upper chamber (N=3), and effect of NONO inhibitor on UCP1 protein expression levels in HIB1B cells co-cultured with primary hepatocytes in the lower chamber (N=3).G）Co-culture experiment: effect of ADM knockdown on ADM protein levels in primary hepatocytes of the upper chamber (N=3), and effect of NONO inhibitor on UCP1 protein expression levels in HIB1B cells co-cultured with primary hepatocytes in the lower chamber (N=3). **P*<0.05 versus control cells or between two indicated groups.
