# Supplementary material for: Hepatic ENTPD5 Is Critical for Maintaining Metabolic Homeostasis and Promoting Brown Adipose Tissue Thermogenesis
Source: Adv Sci (Weinh). 2025 Aug 11;12(40):e03603. doi: 10.1002/advs.202503603 (PMC12561356; doi:10.1002/advs.202503603)
Supplement: Supplementary file 2 — Supporting Information [file ADVS-12-e03603-s001.pptx]

## Slide 1
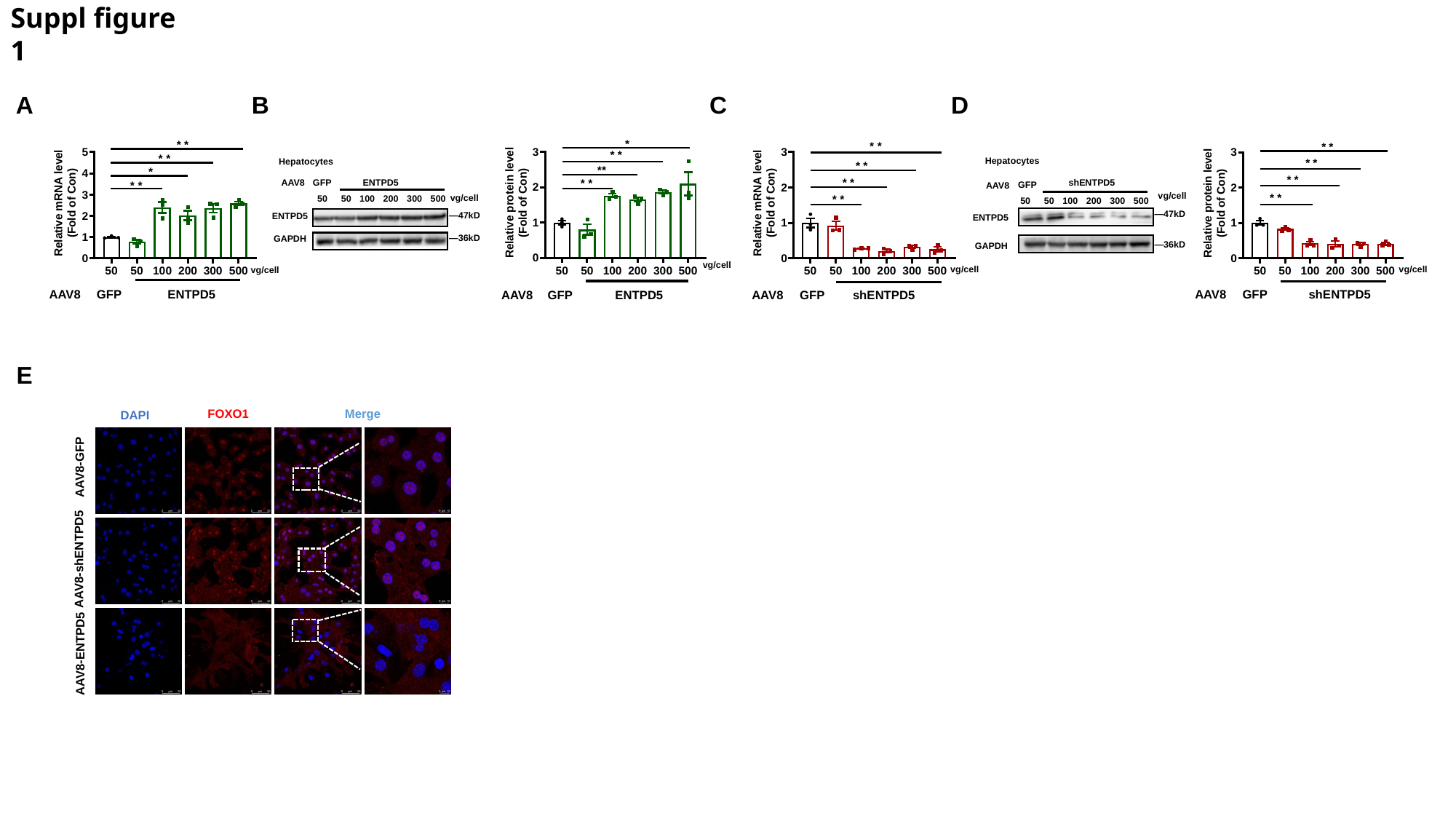

Suppl figure 1
A
B
C
D
AAV8
GFP
ENTPD5
AAV8
GFP
shENTPD5
vg/cell
AAV8
GFP
ENTPD5
AAV8
GFP
shENTPD5
Hepatocytes
GFP
AAV8
shENTPD5
500
50
50
100
200
300
—47kD
ENTPD5
—36kD
GAPDH
Hepatocytes
AAV8
GFP
ENTPD5
vg/cell
50
50
100
200
300
500
—47kD
ENTPD5
—36kD
GAPDH
vg/cell
vg/cell
vg/cell
vg/cell
E
FOXO1
Merge
DAPI
AAV8-GFP
AAV8-shENTPD5
AAV8-ENTPD5

## Slide 2
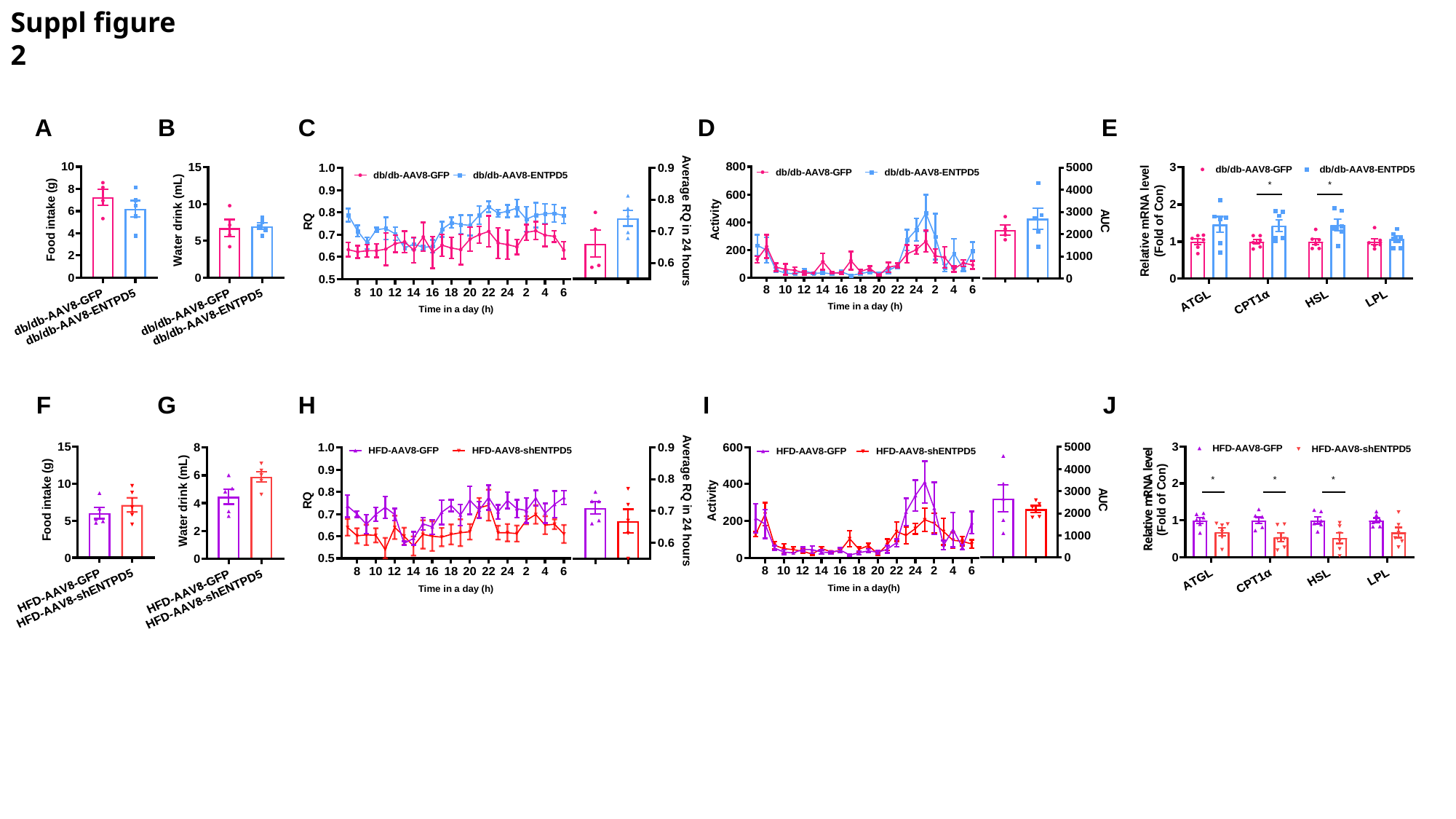

Suppl figure 2
A
B
C
D
E
F
G
H
I
J

## Slide 3
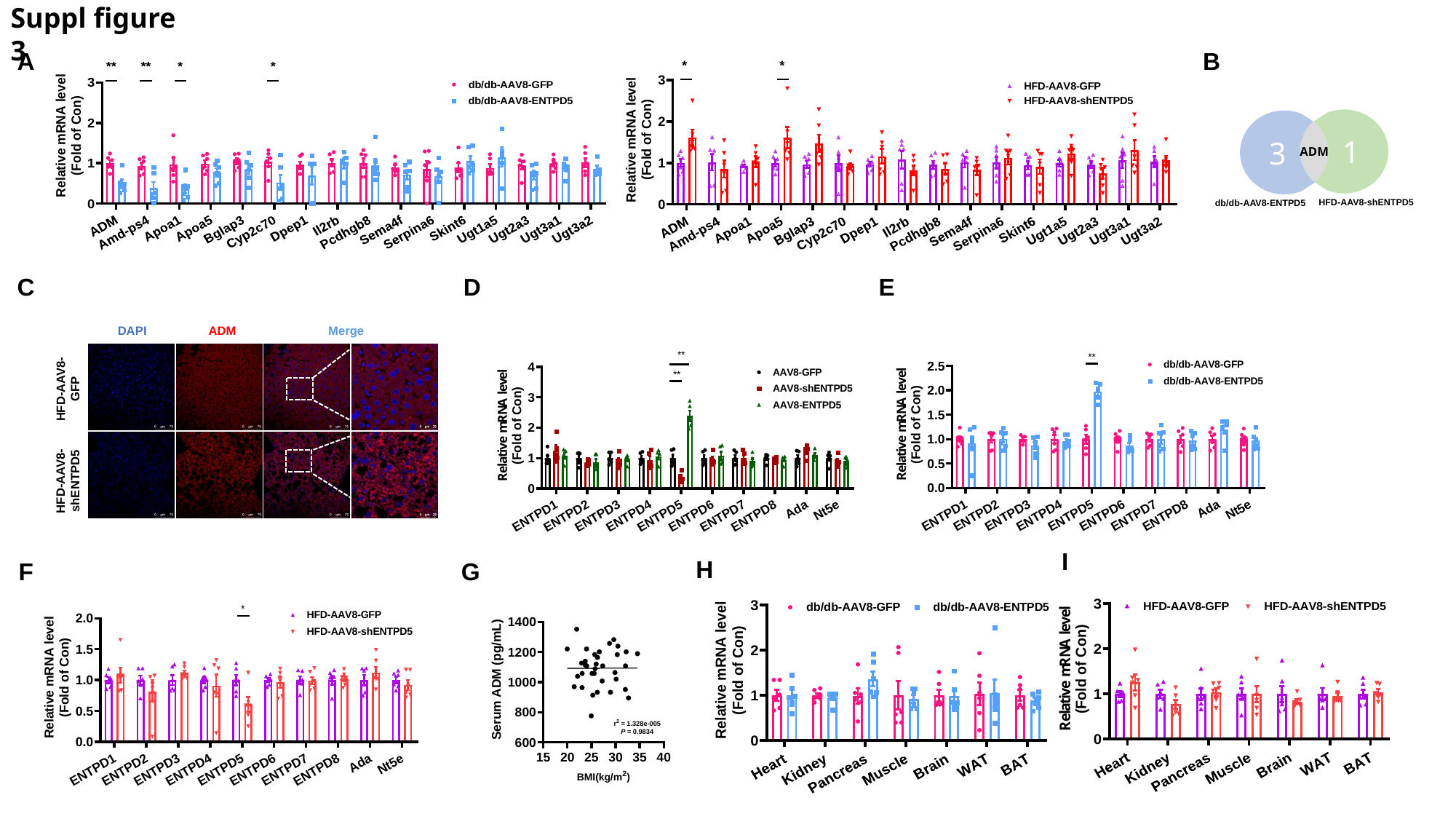

Suppl figure 3
A
B
1
3
ADM
HFD-AAV8-shENTPD5
db/db-AAV8-ENTPD5
C
D
E
DAPI
ADM
Merge
HFD-AAV8-
GFP
HFD-AAV8-
shENTPD5
I
H
F
G

## Slide 4
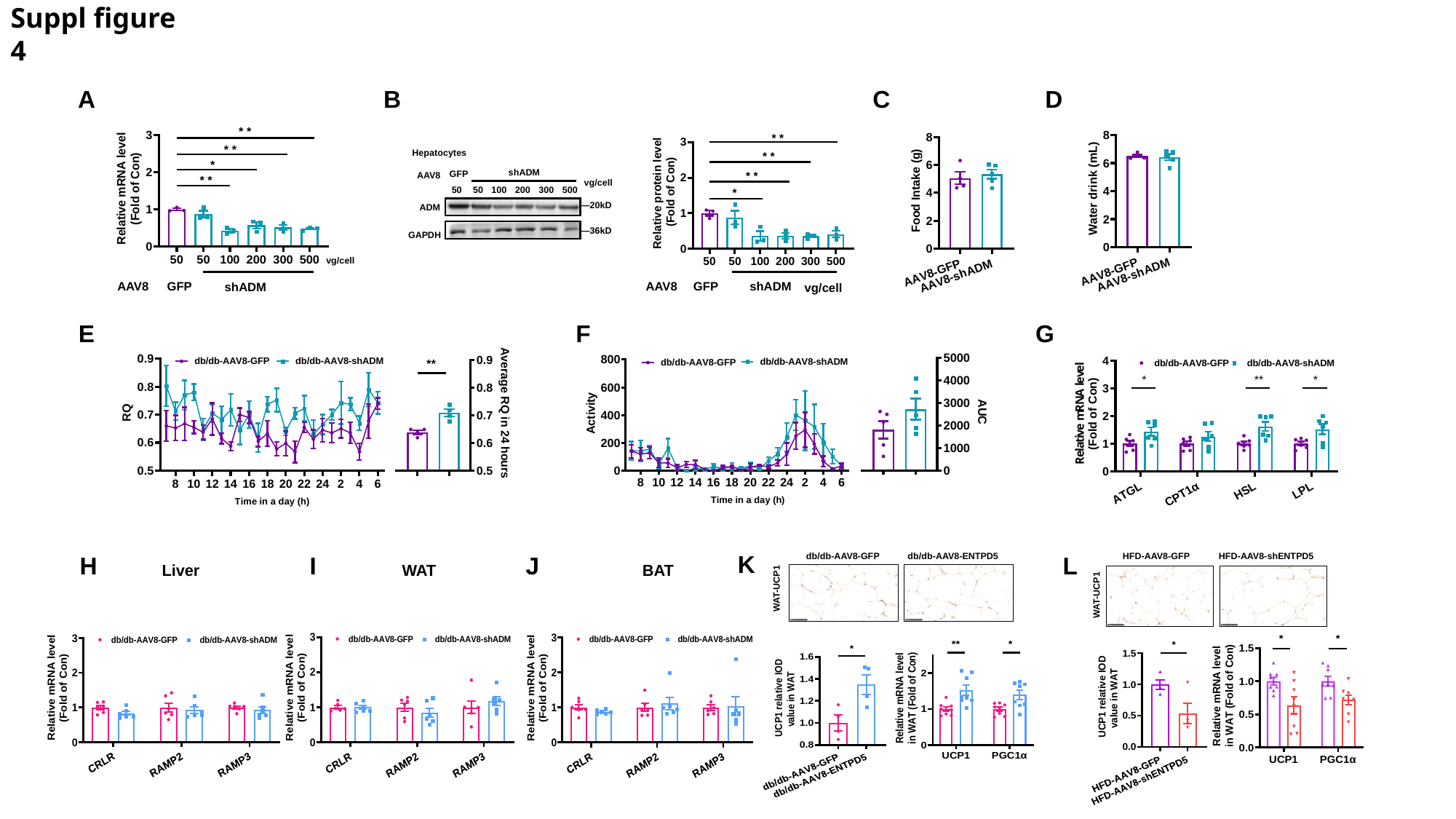

Suppl figure 4
A
B
C
D
AAV8
GFP
shADM
vg/cell
AAV8
GFP
shADM
Hepatocytes
GFP
shADM
AAV8
500
50
50
100
200
300
—20kD
ADM
—36kD
GAPDH
vg/cell
vg/cell
E
F
G
K
db/db-AAV8-ENTPD5
db/db-AAV8-GFP
WAT-UCP1
H
I
J
L
HFD-AAV8-shENTPD5
HFD-AAV8-GFP
WAT-UCP1
Liver
WAT
BAT

## Slide 5
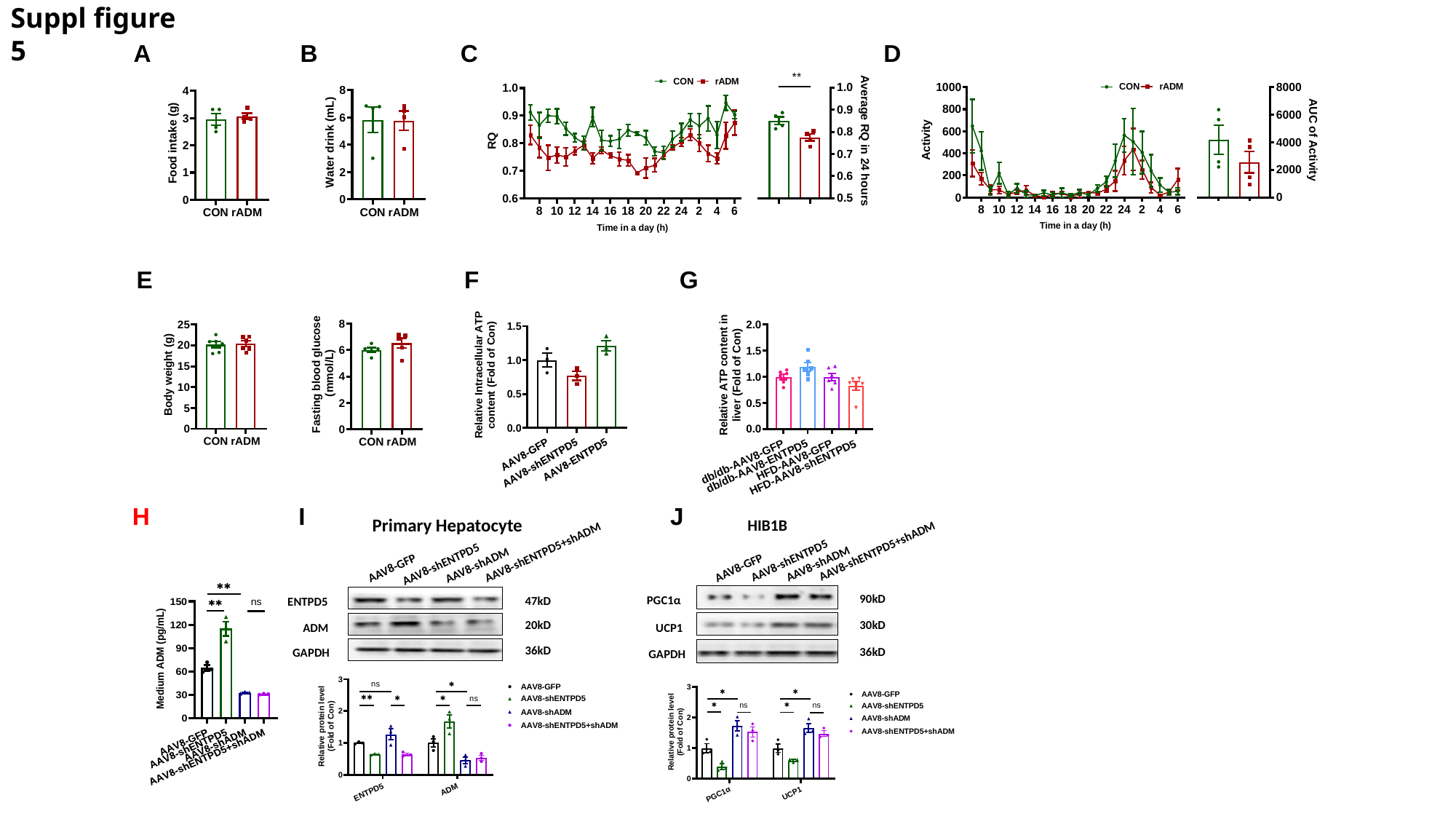

Suppl figure 5
A
B
C
D
E
F
G
H
I
J
Primary Hepatocyte
AAV8-shENTPD5+shADM
AAV8-shADM
AAV8-GFP
ENTPD5
ADM
GAPDH
47kD
20kD
36kD
AAV8-shENTPD5
HIB1B
AAV8-shENTPD5
AAV8-shADM
AAV8-GFP
90kD
PGC1α
UCP1
GAPDH
30kD
36kD
AAV8-shENTPD5+shADM

## Slide 6
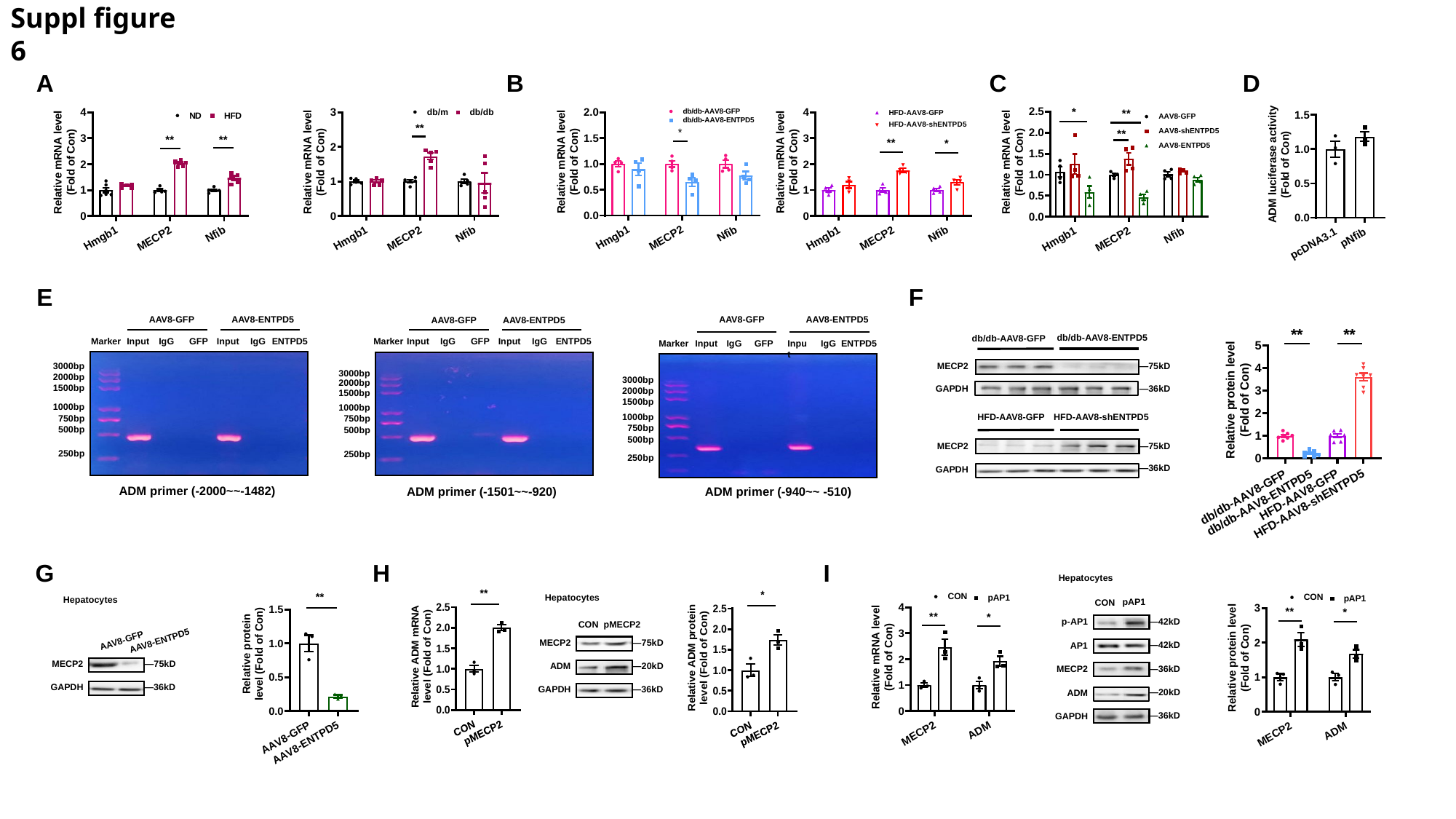

Suppl figure 6
A
B
C
D
E
F
AAV8-GFP
AAV8-ENTPD5
Marker
Input
IgG
GFP
Input
IgG
ENTPD5
3000bp
2000bp
1500bp
1000bp
750bp
500bp
250bp
ADM primer (-940~~ -510)
AAV8-GFP
AAV8-ENTPD5
Marker
Input
IgG
GFP
Input
IgG
ENTPD5
3000bp
2000bp
1500bp
1000bp
750bp
500bp
250bp
ADM primer (-2000~~-1482)
AAV8-GFP
AAV8-ENTPD5
Marker
Input
IgG
GFP
Input
IgG
ENTPD5
3000bp
2000bp
1500bp
1000bp
750bp
500bp
250bp
ADM primer (-1501~~-920)
db/db-AAV8-ENTPD5
db/db-AAV8-GFP
—75kD
MECP2
—36kD
GAPDH
HFD-AAV8-GFP
HFD-AAV8-shENTPD5
—75kD
MECP2
—36kD
GAPDH
G
H
I
Hepatocytes
pAP1
CON
—42kD
p-AP1
—42kD
AP1
—36kD
MECP2
—20kD
ADM
—36kD
GAPDH
Hepatocytes
CON
pMECP2
—75kD
MECP2
—20kD
ADM
—36kD
GAPDH
Hepatocytes
AAV8-GFP
AAV8-ENTPD5
—75kD
MECP2
—36kD
GAPDH

## Slide 7
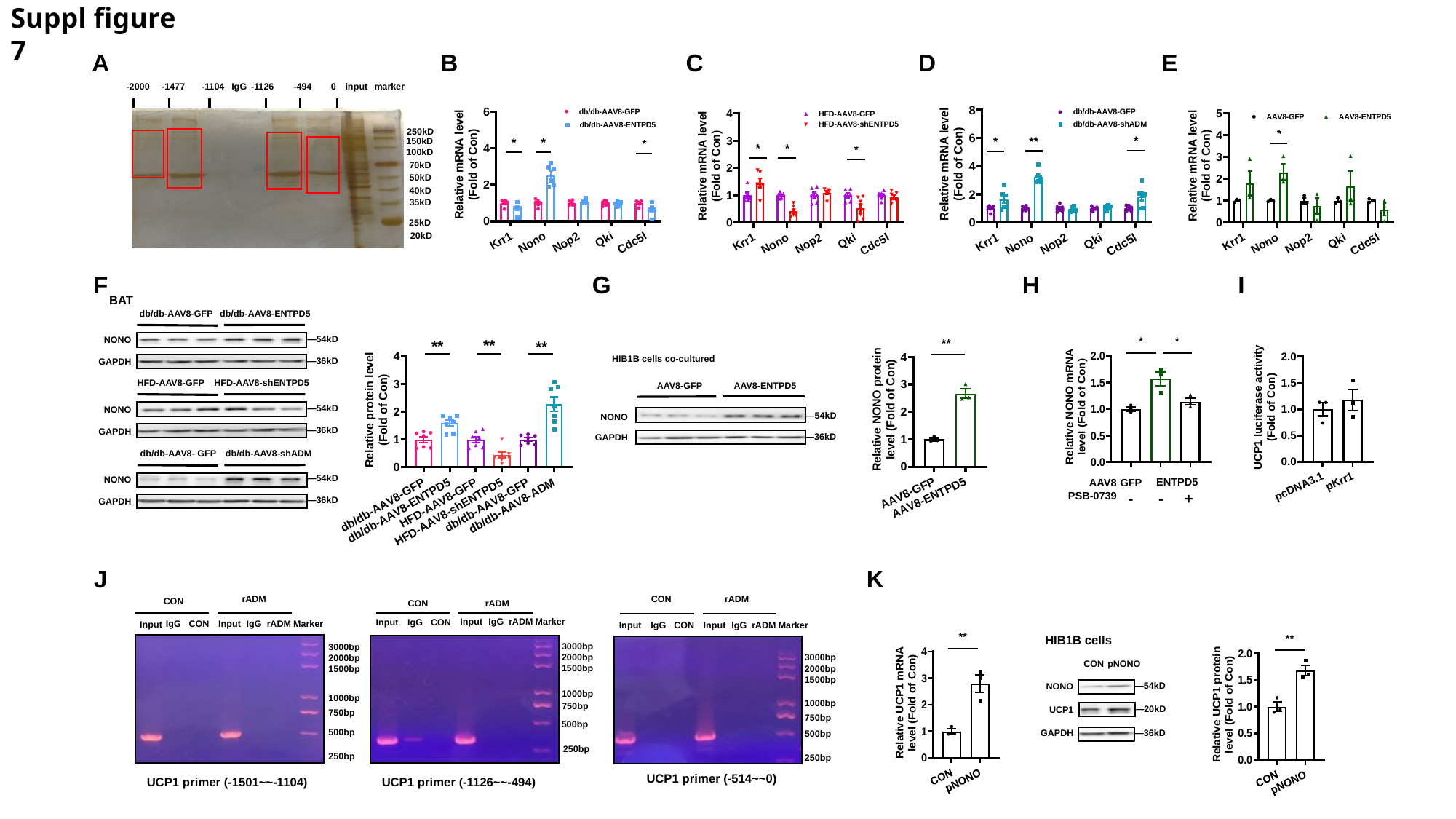

Suppl figure 7
A
B
C
D
E
-2000
-1477
-1104
IgG
-1126
-494
0
input
marker
250kD
150kD
100kD
70kD
50kD
40kD
35kD
25kD
20kD
F
G
H
I
BAT
db/db-AAV8-GFP
db/db-AAV8-ENTPD5
—54kD
NONO
—36kD
GAPDH
HFD-AAV8-GFP
HFD-AAV8-shENTPD5
—54kD
NONO
—36kD
GAPDH
db/db-AAV8- GFP
db/db-AAV8-shADM
—54kD
NONO
—36kD
GAPDH
ENTPD5
GFP
AAV8
PSB-0739
- - +
HIB1B cells co-cultured
AAV8-GFP
AAV8-ENTPD5
—54kD
NONO
GAPDH
—36kD
J
K
CON
rADM
Input
IgG
CON
Input
IgG
rADM
Marker
3000bp
2000bp
1500bp
1000bp
750bp
500bp
250bp
UCP1 primer (-514~~0)
rADM
CON
IgG
rADM
Marker
Input
IgG
CON
Input
3000bp
2000bp
1500bp
1000bp
750bp
500bp
250bp
UCP1 primer (-1501~~-1104)
CON
rADM
IgG
rADM
Marker
Input
IgG
CON
Input
3000bp
2000bp
1500bp
1000bp
750bp
500bp
250bp
UCP1 primer (-1126~~-494)
HIB1B cells
pNONO
CON
—54kD
NONO
—20kD
UCP1
—36kD
GAPDH

## Slide 8
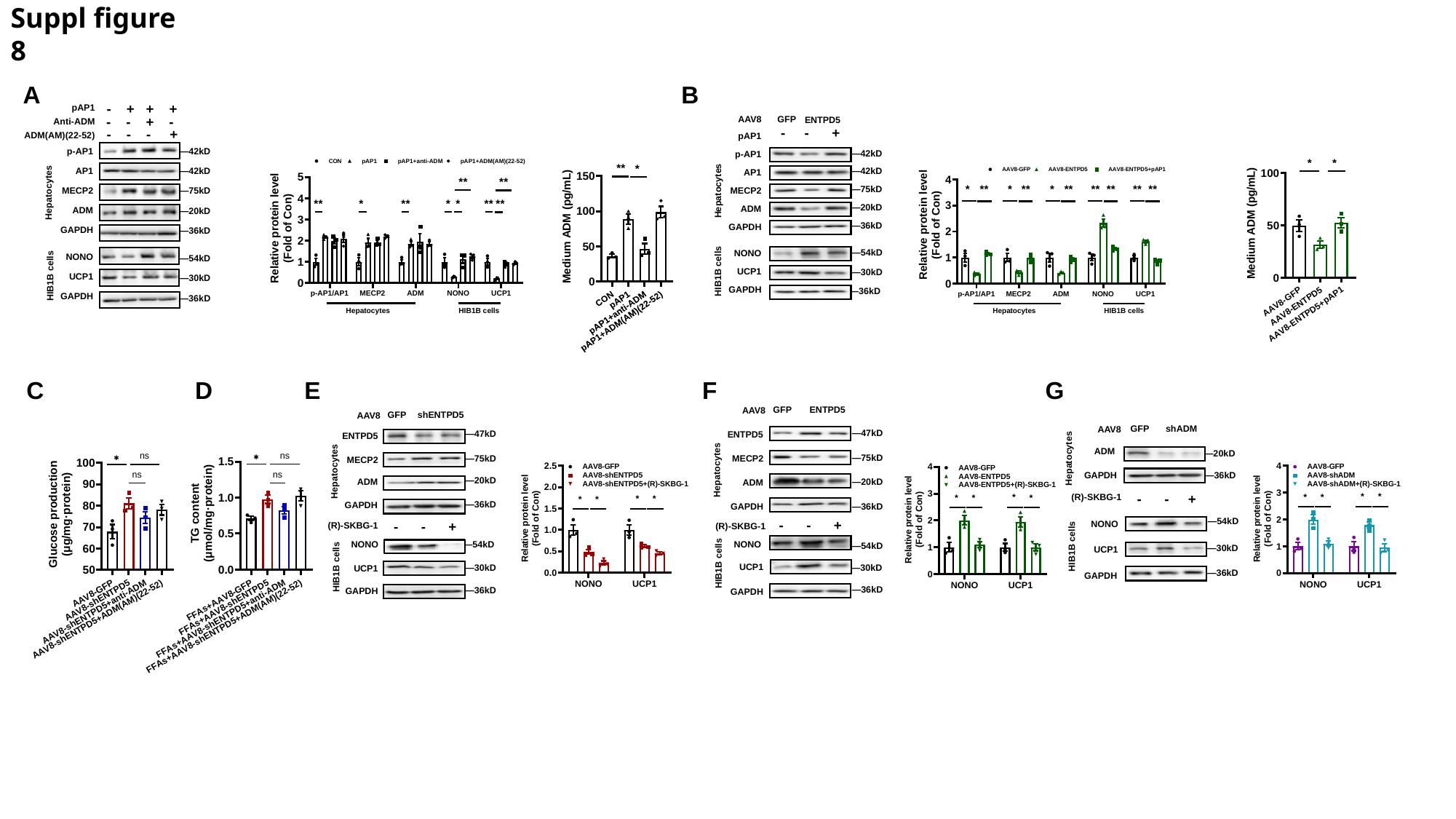

Suppl figure 8
A
B
- + + +
pAP1
- - + -
Anti-ADM
- - - +
ADM(AM)(22-52)
—42kD
p-AP1
—42kD
AP1
—75kD
Hepatocytes
MECP2
—20kD
ADM
—36kD
GAPDH
—54kD
NONO
HIB1B cells
—30kD
UCP1
GAPDH
—36kD
AAV8
GFP
ENTPD5
- - +
pAP1
—42kD
p-AP1
—42kD
AP1
—75kD
Hepatocytes
MECP2
—20kD
ADM
—36kD
GAPDH
—54kD
NONO
HIB1B cells
—30kD
UCP1
—36kD
GAPDH
C
D
E
F
G
GFP
ENTPD5
AAV8
—47kD
ENTPD5
—75kD
MECP2
Hepatocytes
—20kD
ADM
—36kD
GAPDH
- - +
(R)-SKBG-1
—54kD
NONO
HIB1B cells
—30kD
UCP1
—36kD
GAPDH
GFP
shENTPD5
AAV8
—47kD
ENTPD5
—75kD
MECP2
Hepatocytes
—20kD
ADM
—36kD
GAPDH
(R)-SKBG-1
- - +
—54kD
NONO
HIB1B cells
—30kD
UCP1
—36kD
GAPDH
GFP
shADM
AAV8
—20kD
ADM
Hepatocytes
—36kD
GAPDH
(R)-SKBG-1
- - +
—54kD
NONO
HIB1B cells
—30kD
UCP1
—36kD
GAPDH
