# Supplementary material for: Hepatic ENTPD5 Is Critical for Maintaining Metabolic Homeostasis and Promoting Brown Adipose Tissue Thermogenesis
Source: Adv Sci (Weinh). 2025 Aug 11;12(40):e03603. doi: 10.1002/advs.202503603 (PMC12561356; doi:10.1002/advs.202503603)

Image for Fig. 1G ENTPD5

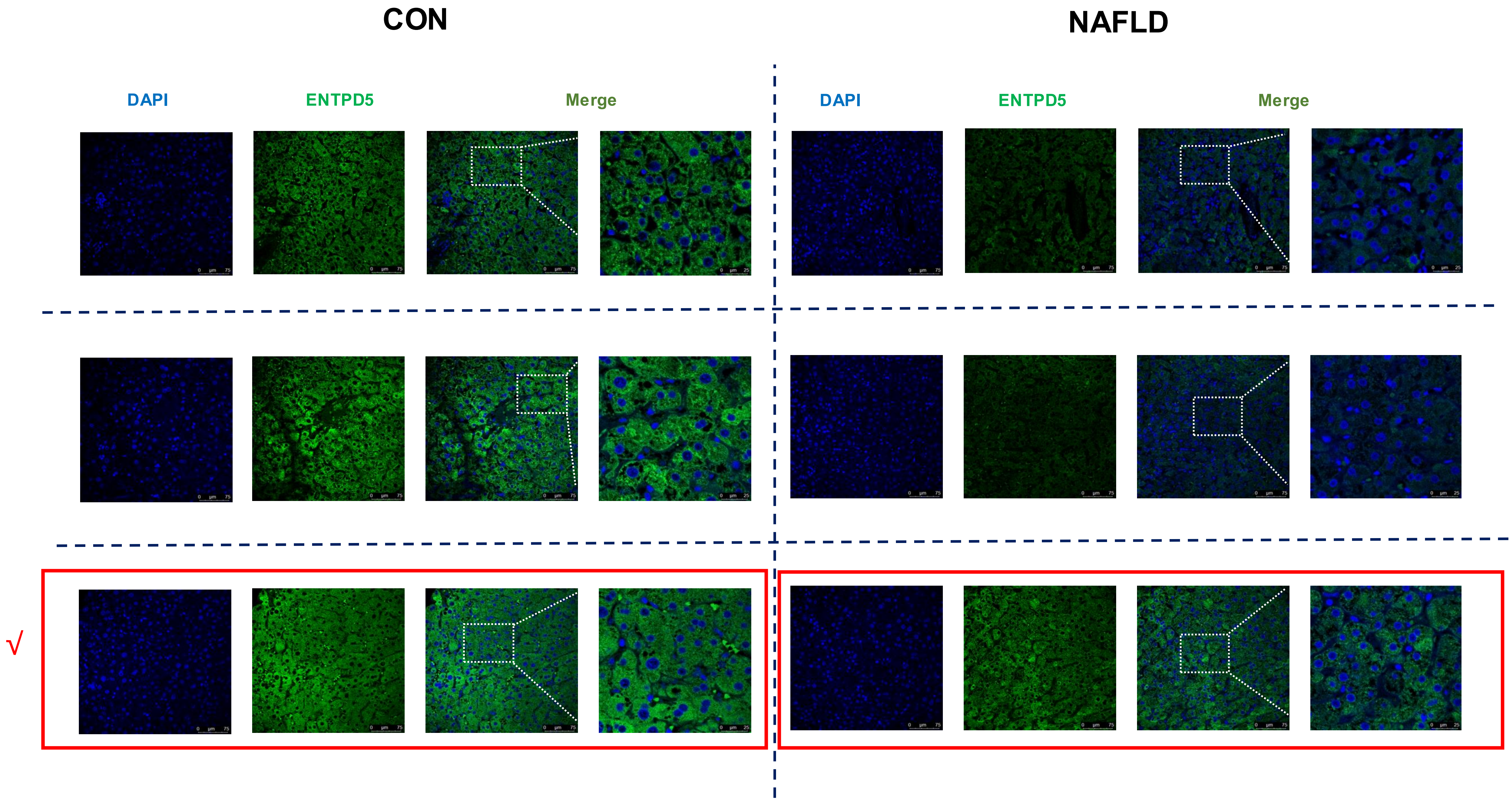

Image for Fig. 1H ENTPD5

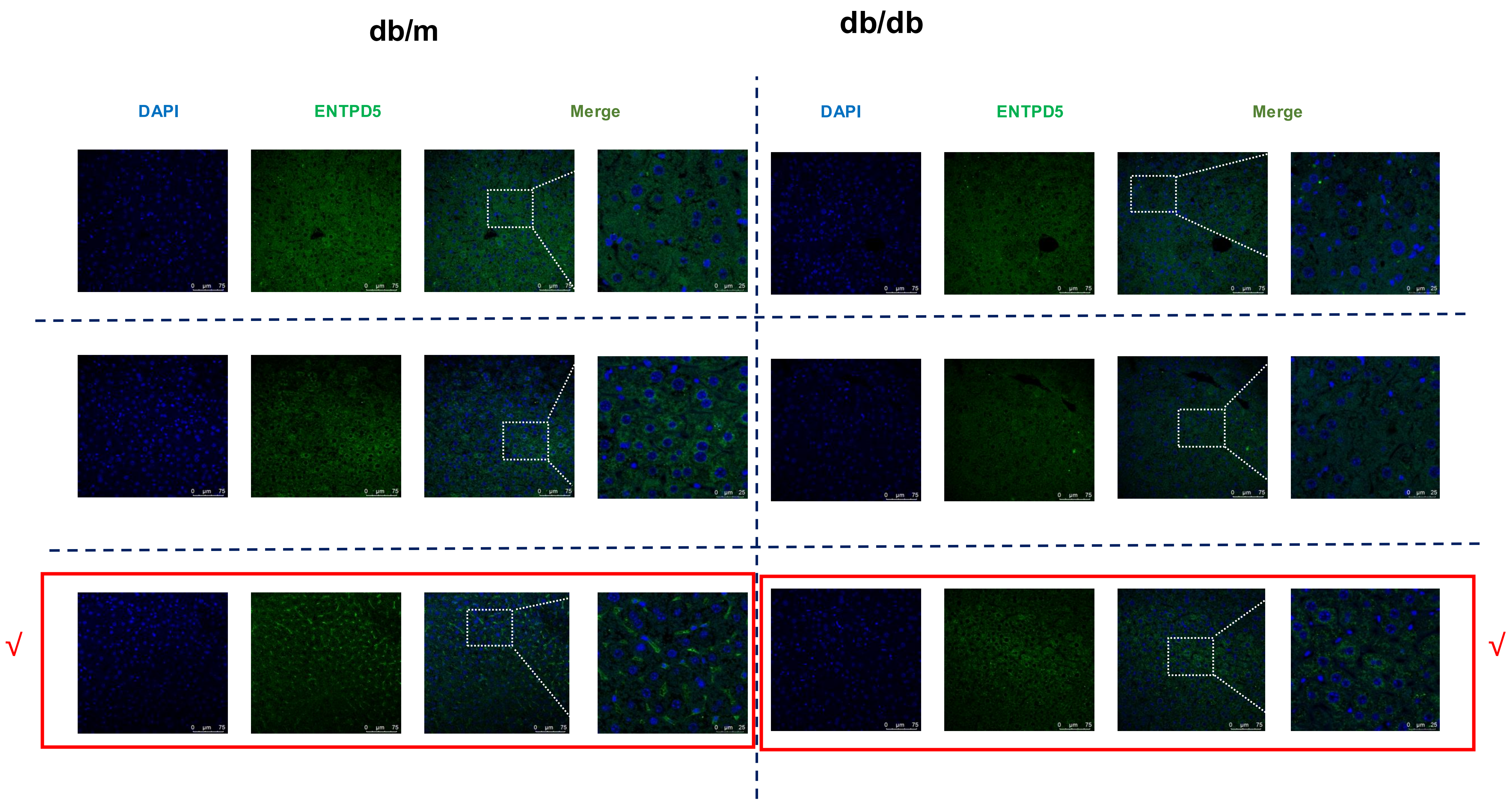

Image for Fig. 1I ENTPD5

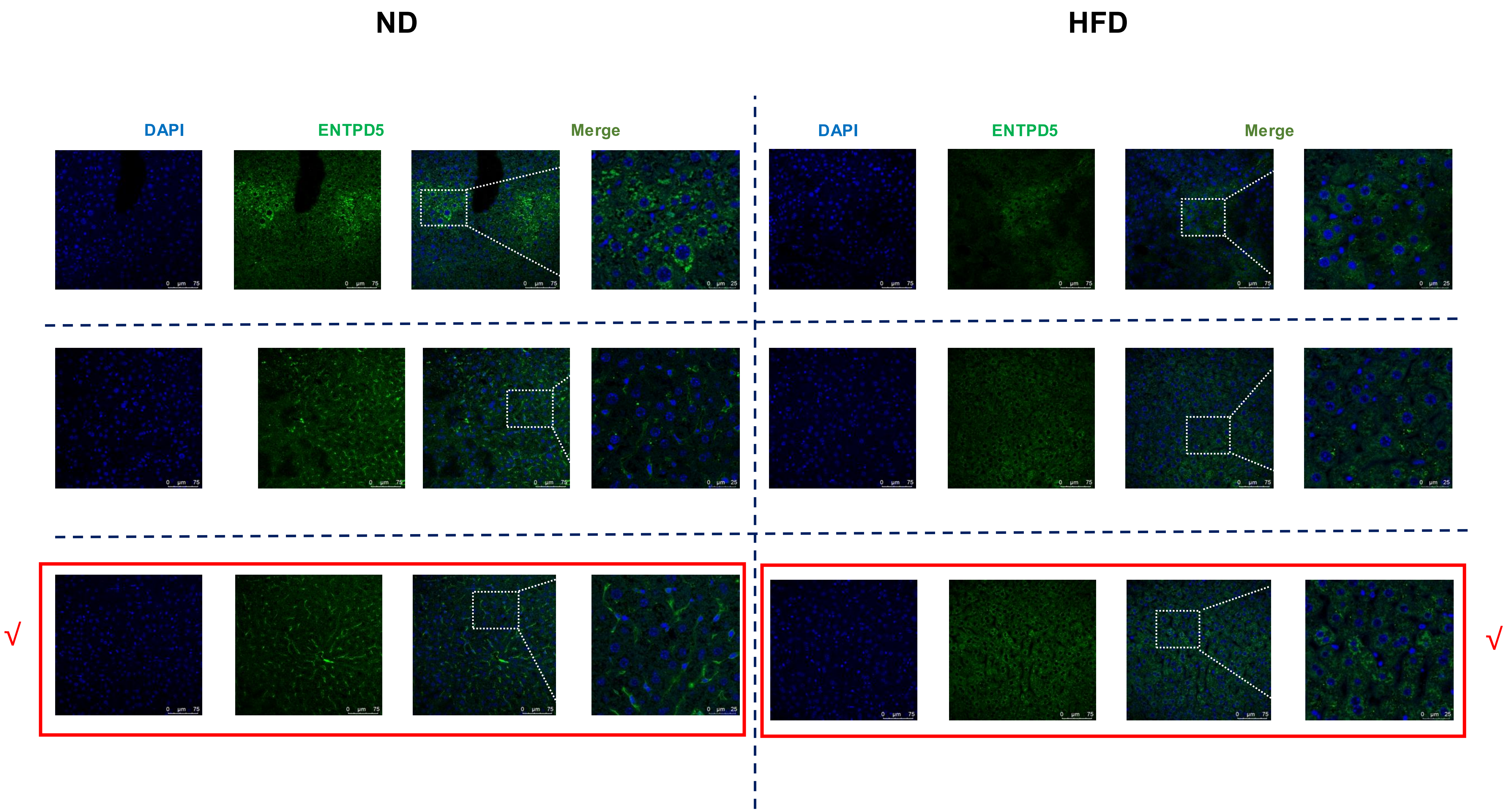

Image for Fig. 1J ENTPD5

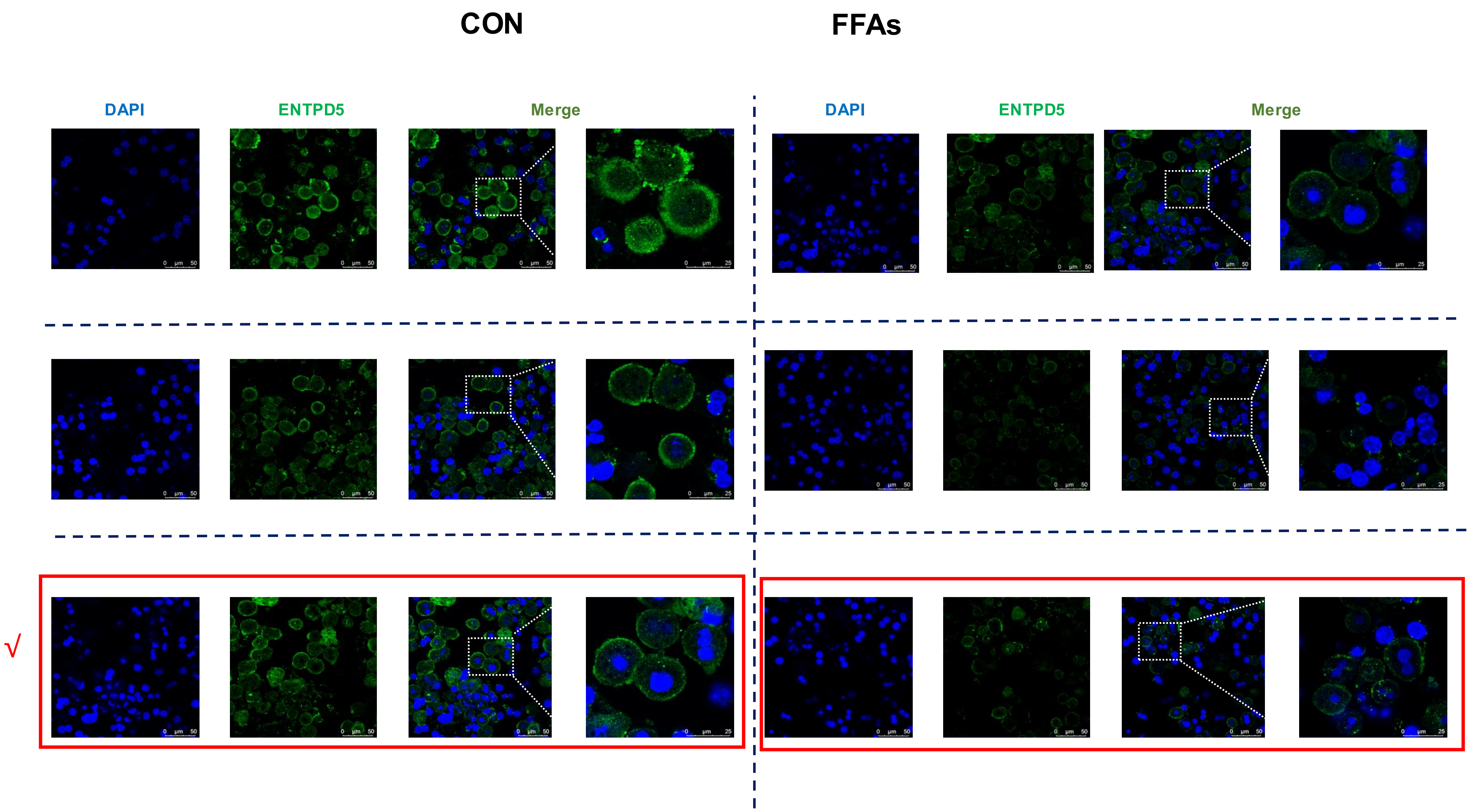

Full unedited gel for Fig. 1K ENTPD5 GAPDH

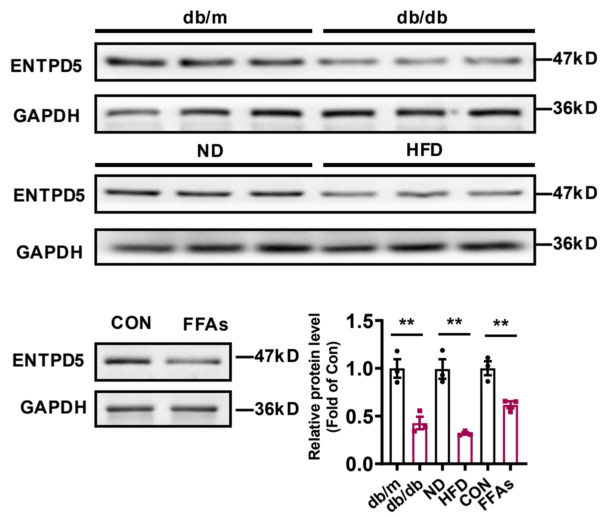

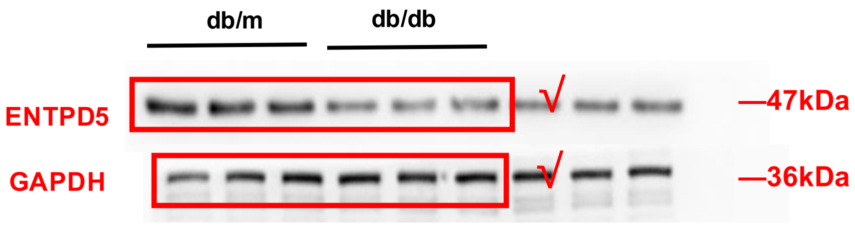

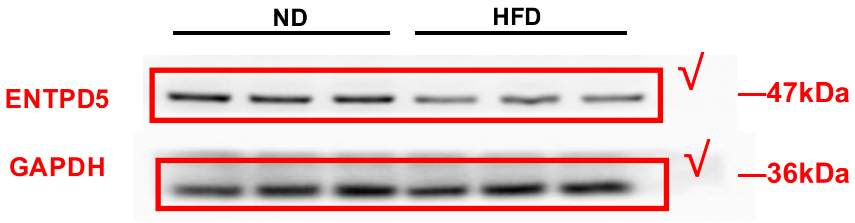

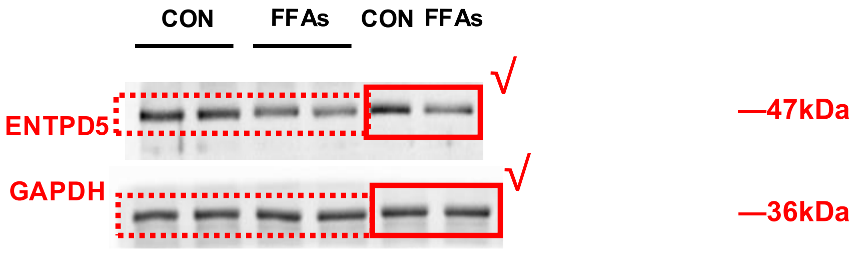

Image for Fig. 1L LIPID

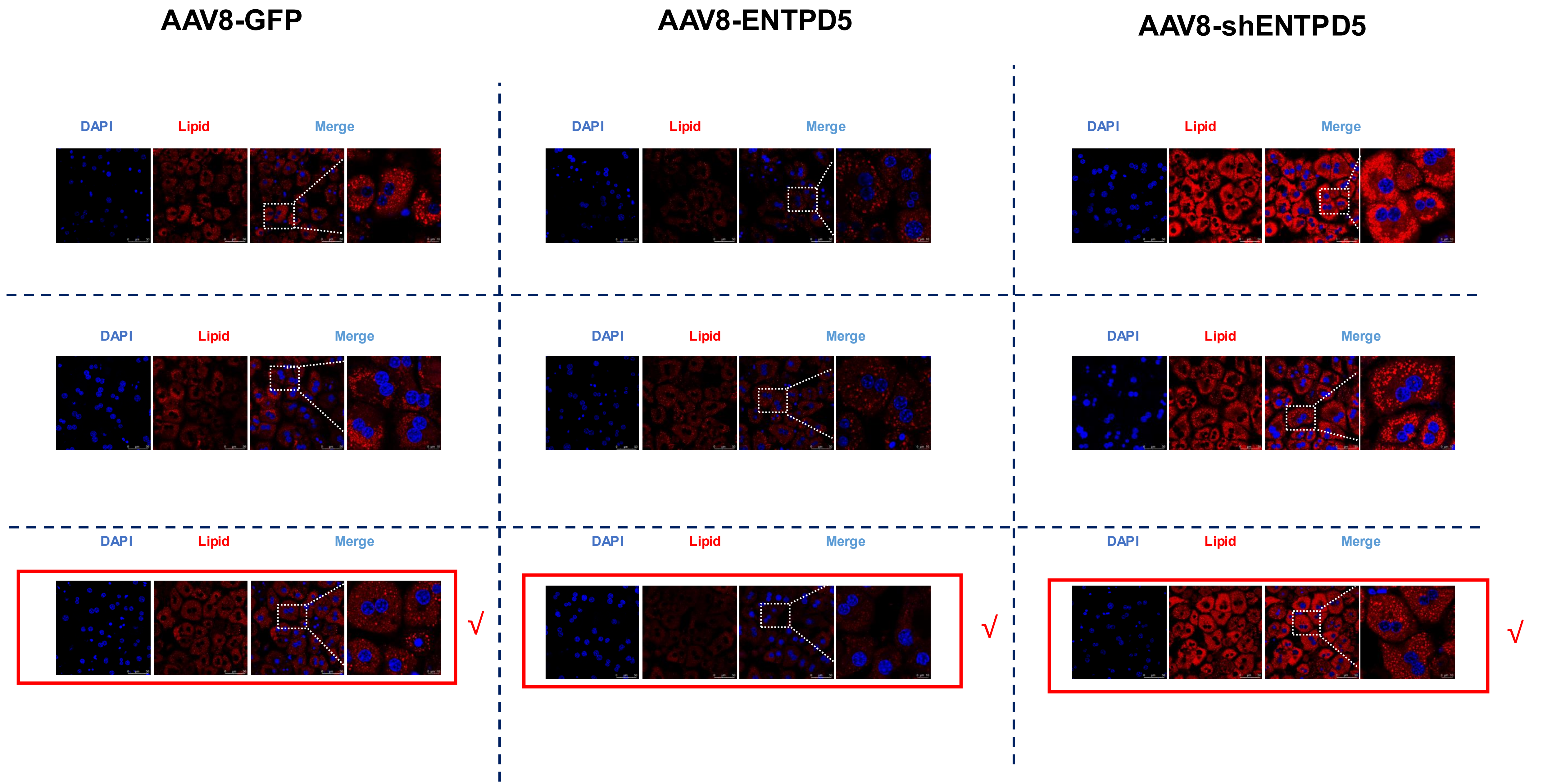

Image for Suppl Fig. 1E

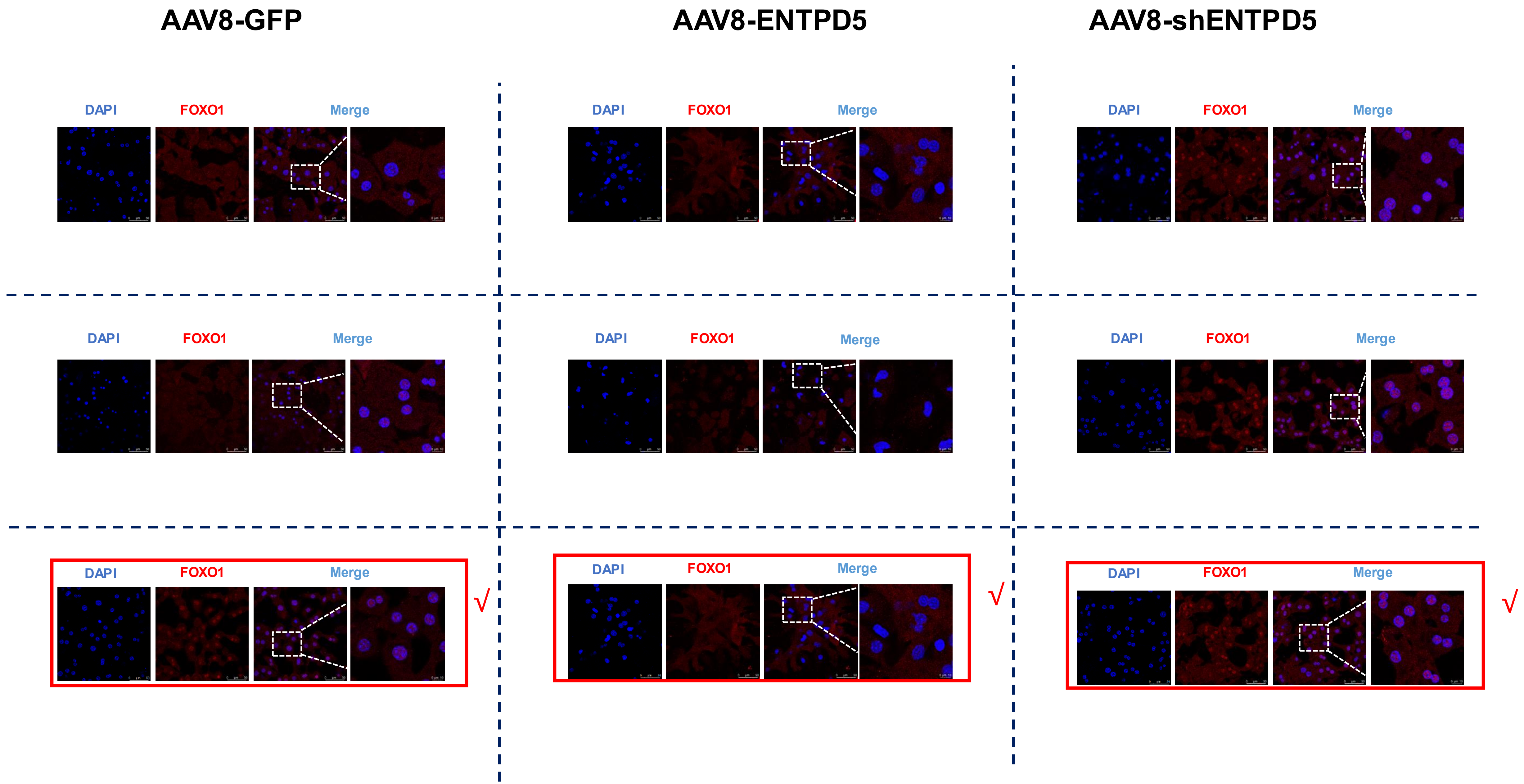

Full unedited gel for S-Fig. 1B ENTPD5 GAPDH

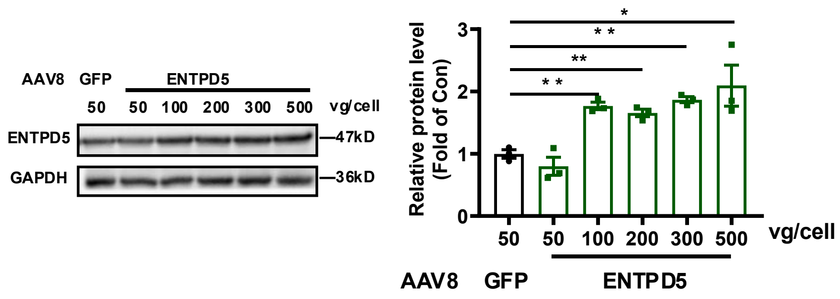

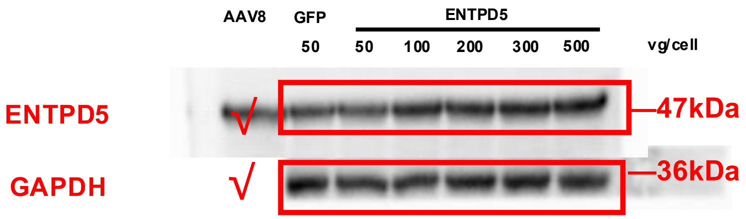

Full unedited gel for S-Fig. 1D ENTPD5 GAPDH

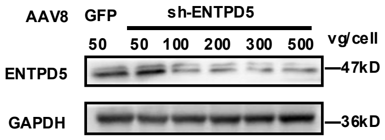

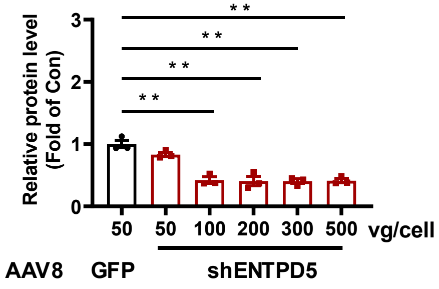

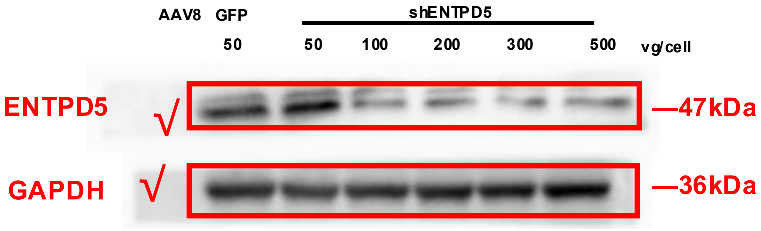

Full unedited gel for Fig. 4F ENTPD5 p-AKT AKT G6Pase PEPCK FASN GAPDH

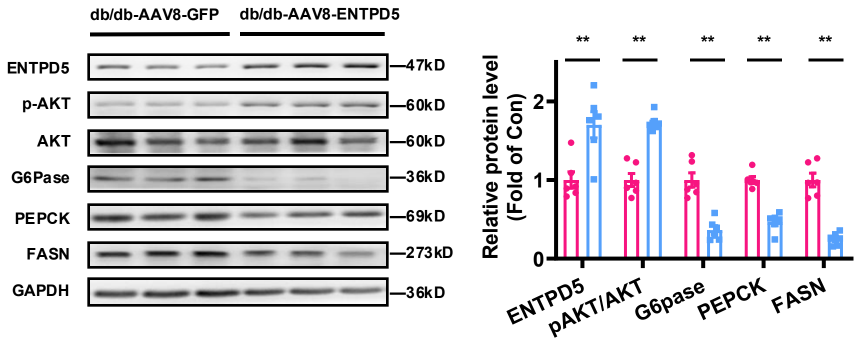

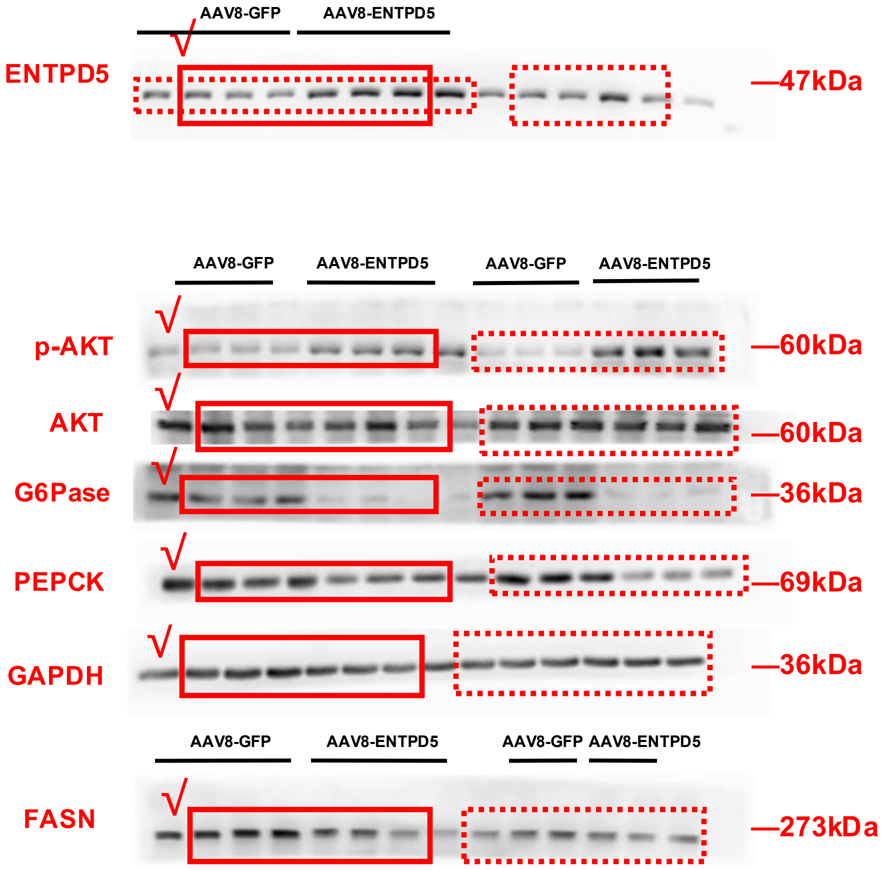

Full unedited gel for Fig. 4L ENTPD5 p-AKT AKT G6Pase PEPCK FASN GAPDH

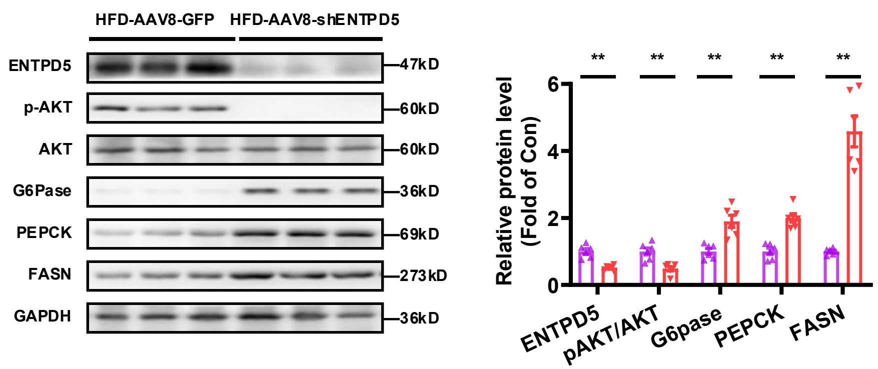

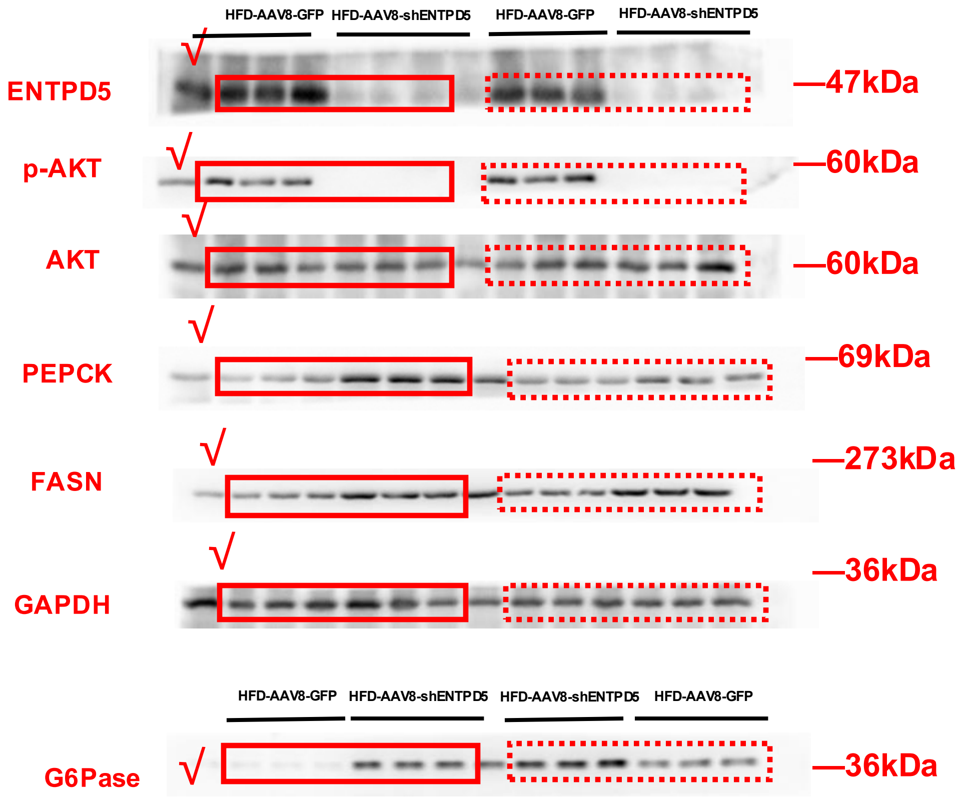

Full unedited gel for Fig.5 B UCP1 PGC1α GAPDH

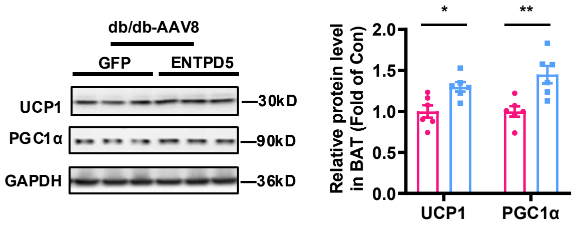

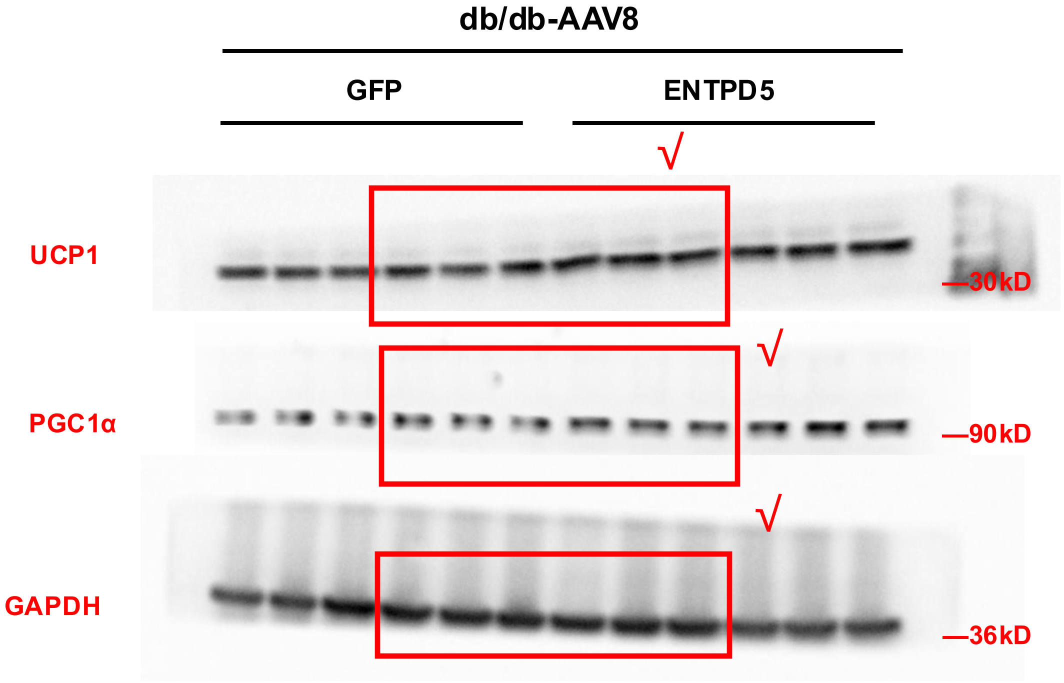

Full unedited gel for Fig.5D UCP1 PGC1α GAPDH

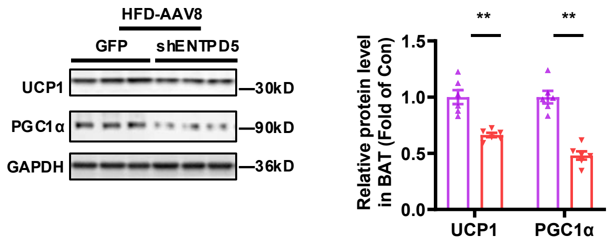

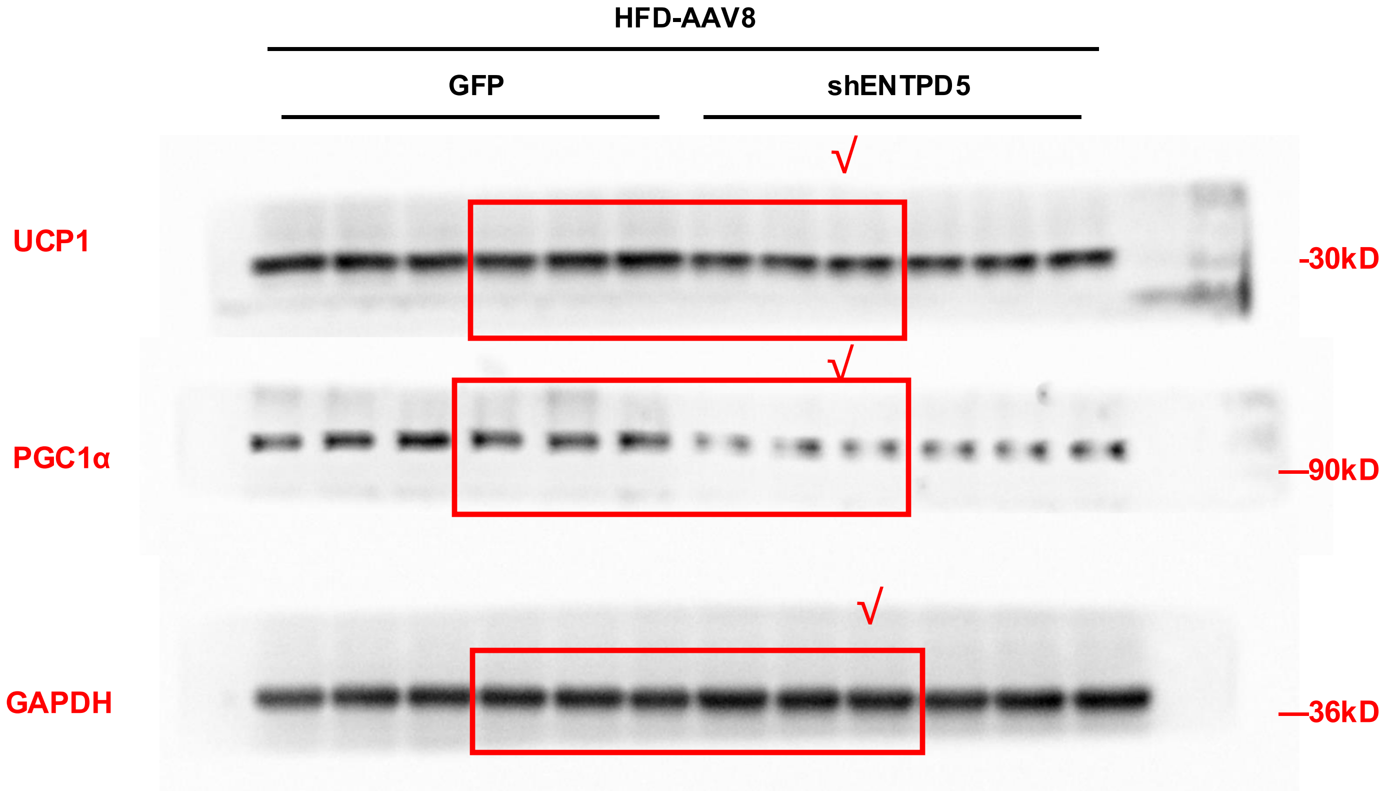

Full unedited gel for Fig.5G ADM GAPDH

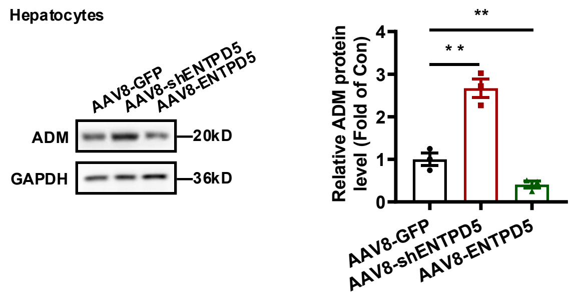

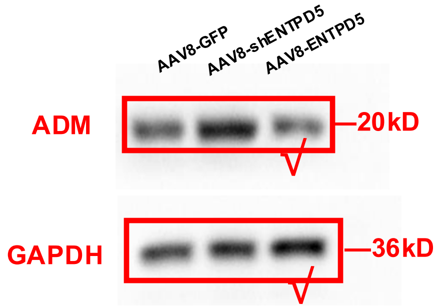

Image for Suppl Fig. 5H ADM

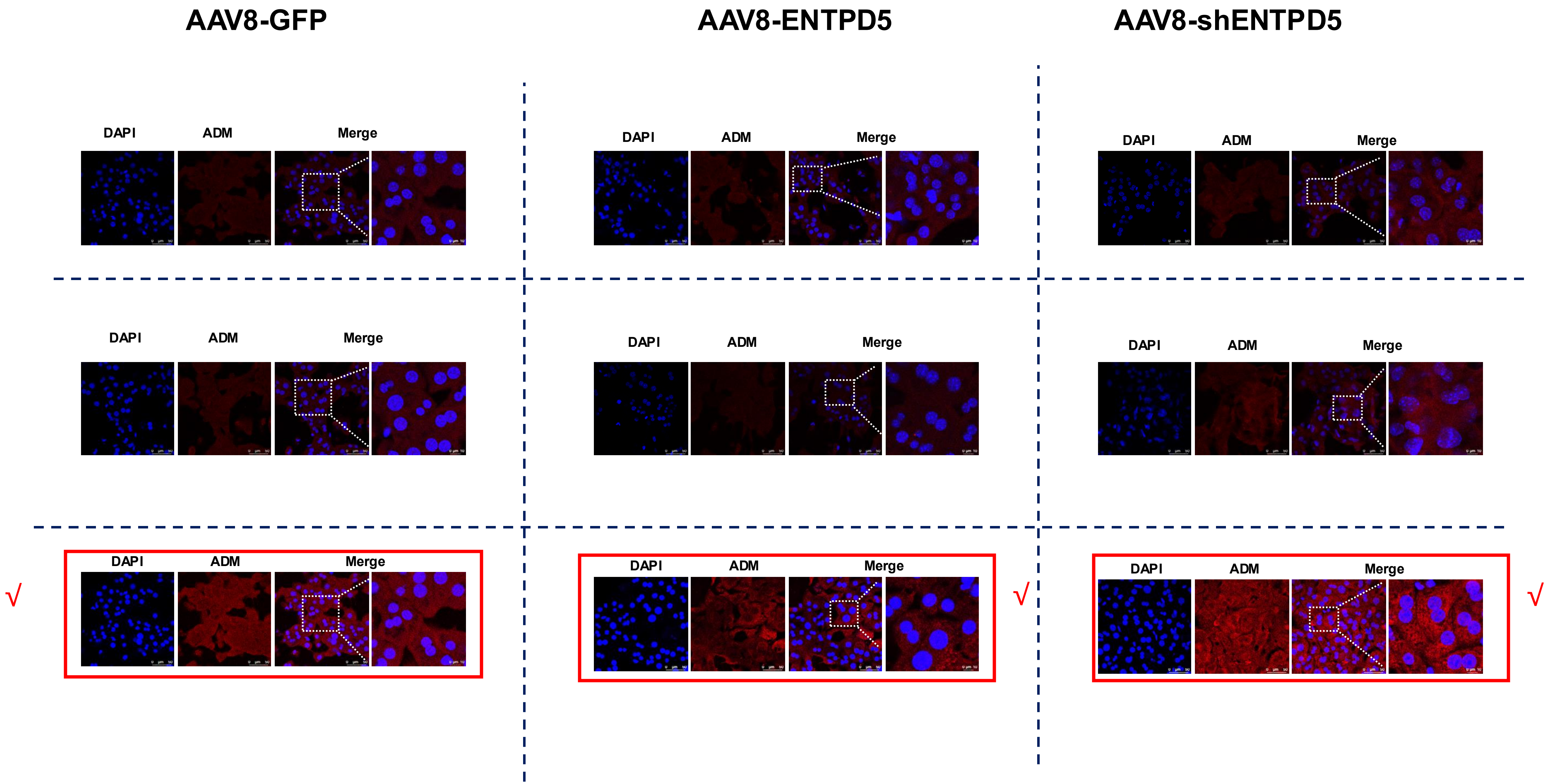

Image for Fig. 5I ADM

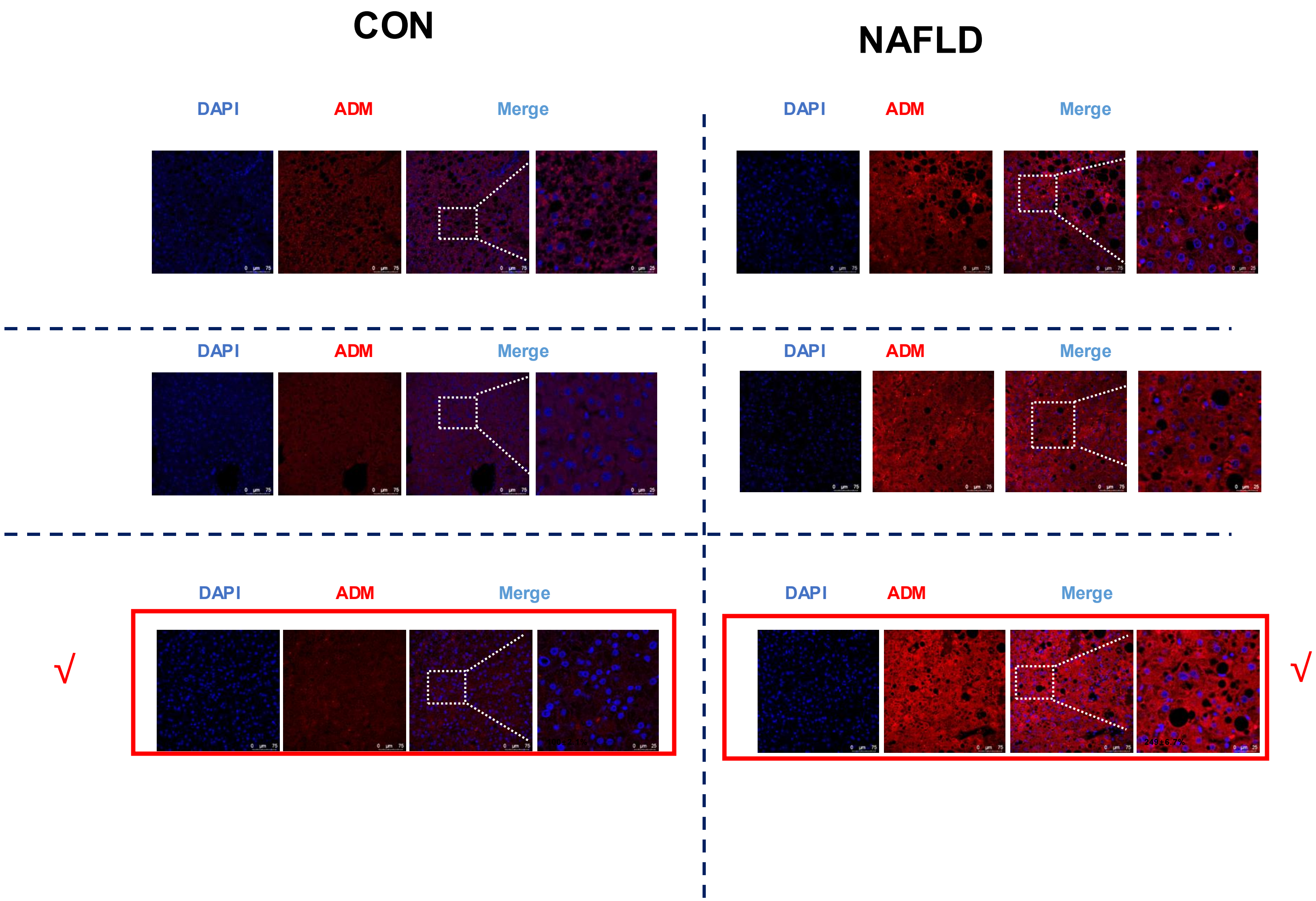

Image for Fig. 5M db/db

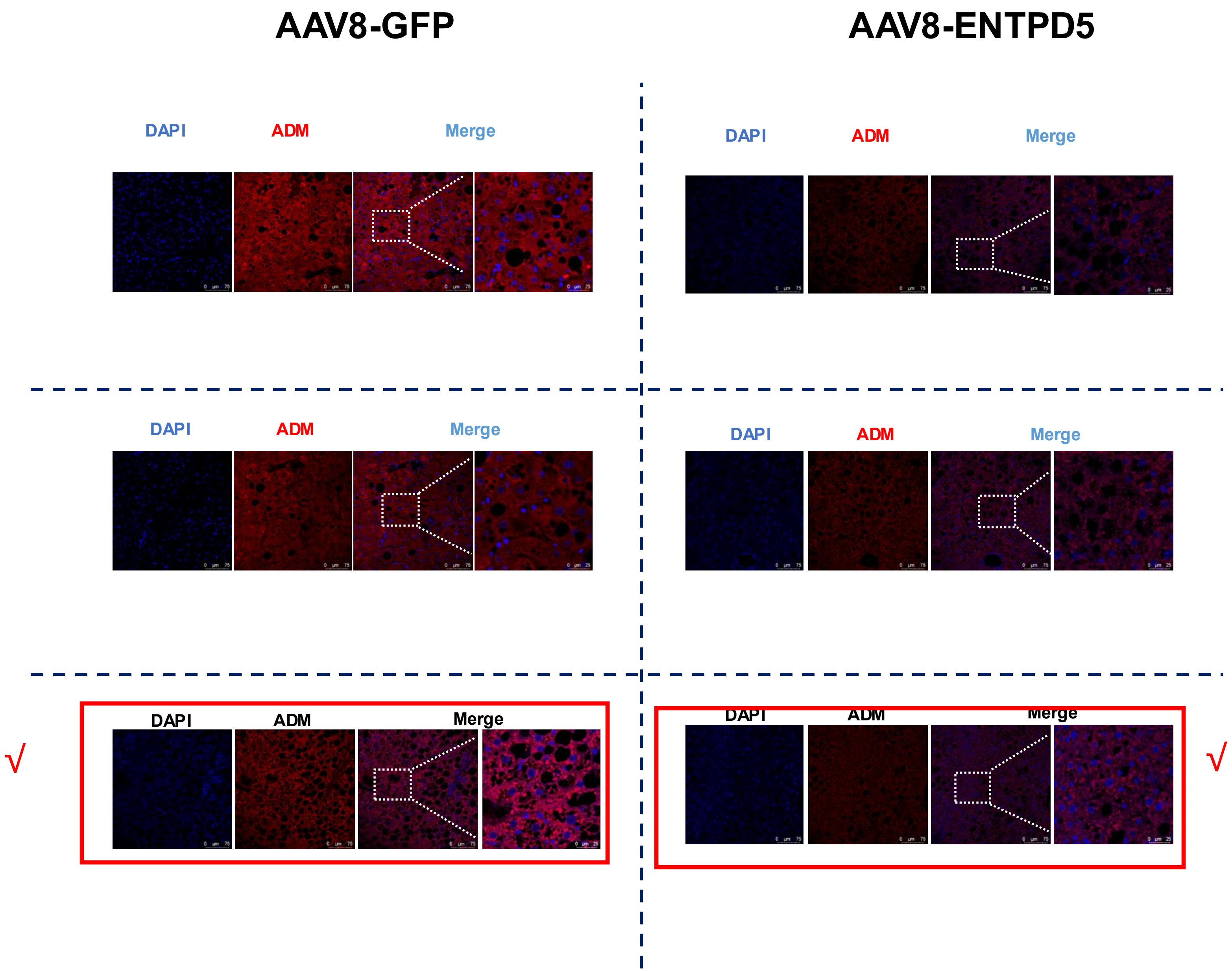

Image for S-Fig. 3C HFD

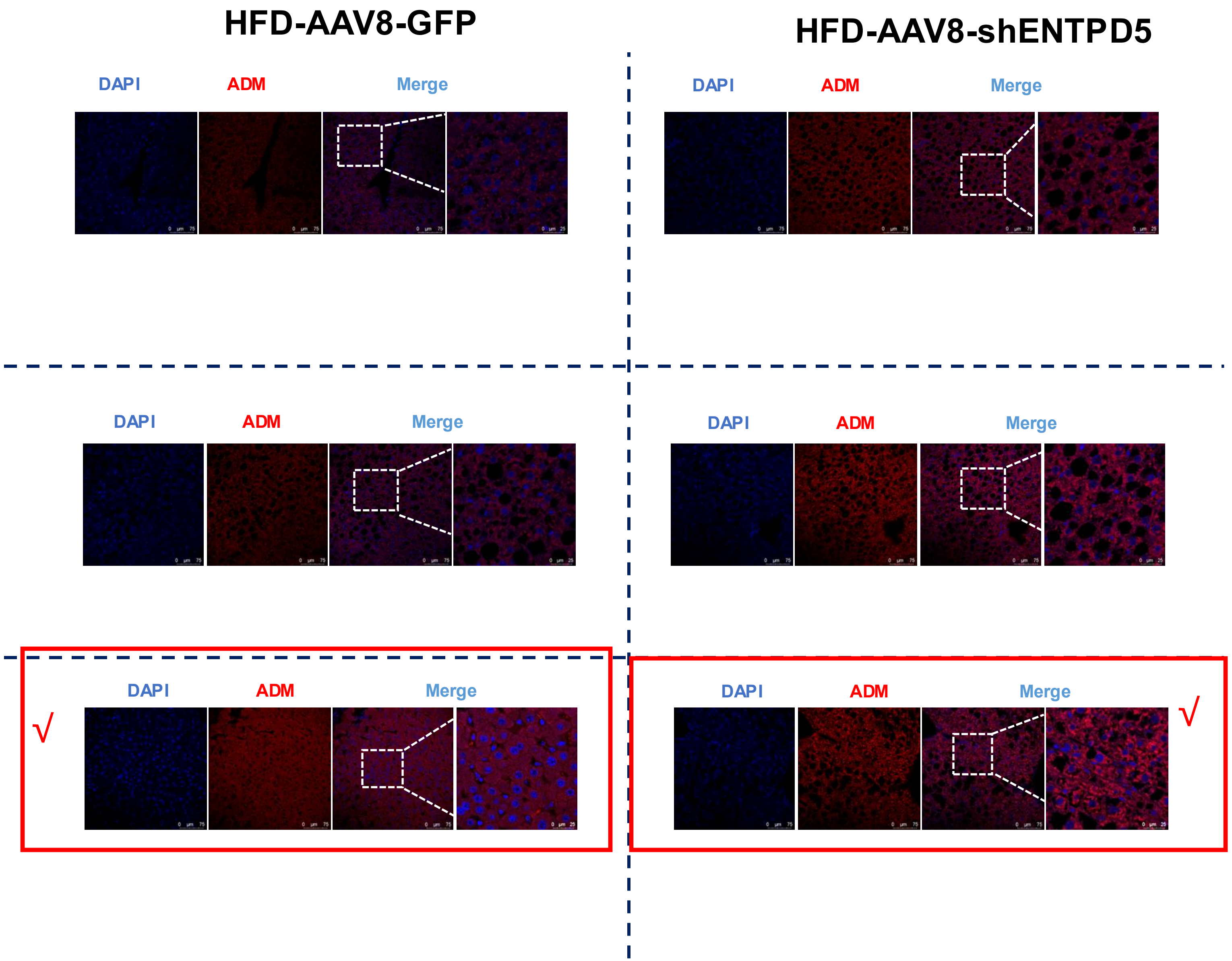

Full unedited gel for Fig.5J ADM GAPDH

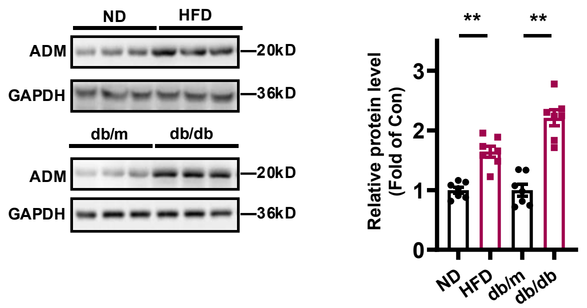

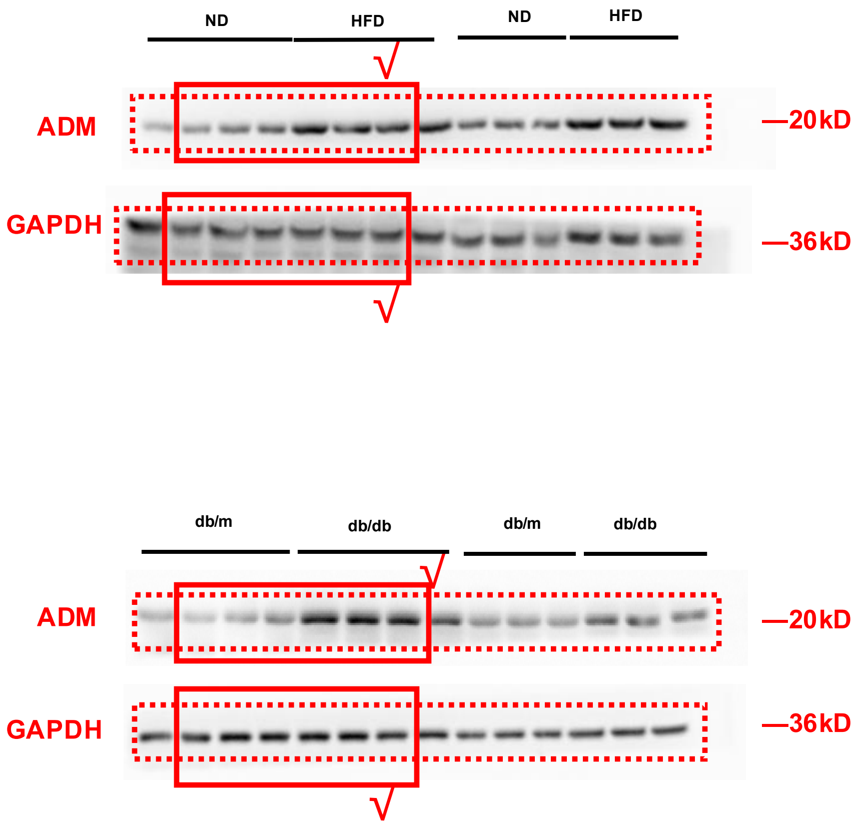

Full unedited gel for Fig.5L ADM GAPDH

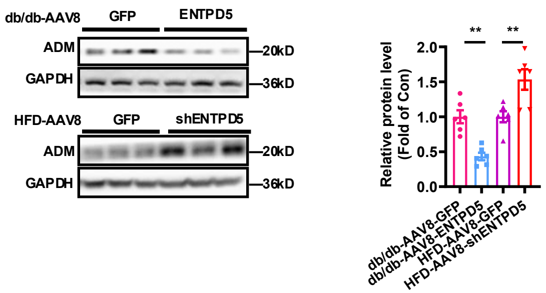

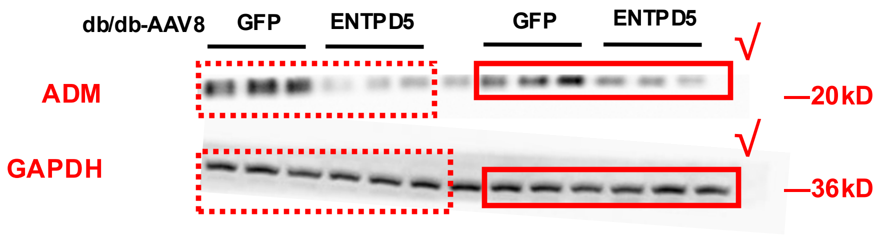

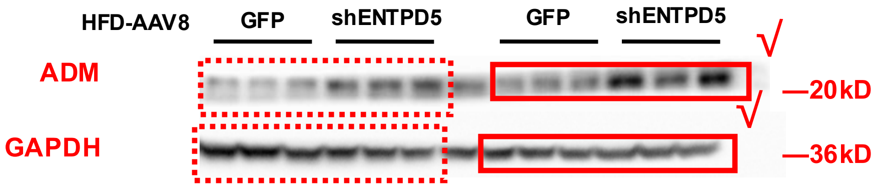

Full unedited gel for Suppl-figure.4B

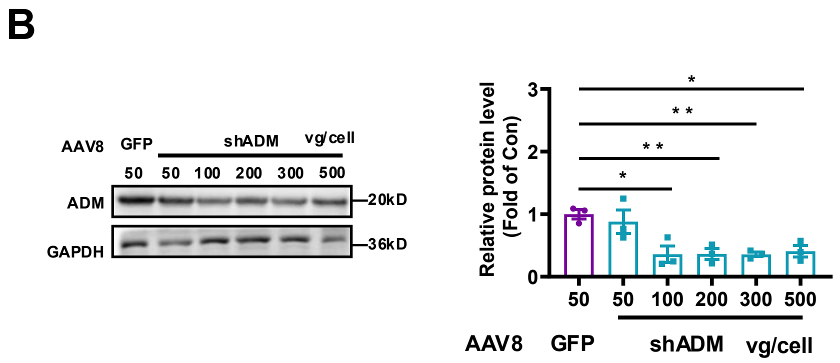

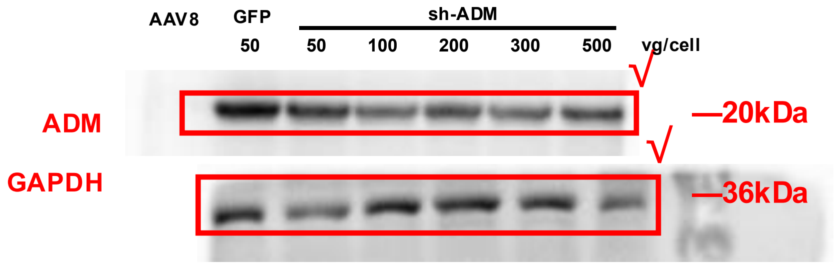

Full unedited gel for Suppl-figure.5H

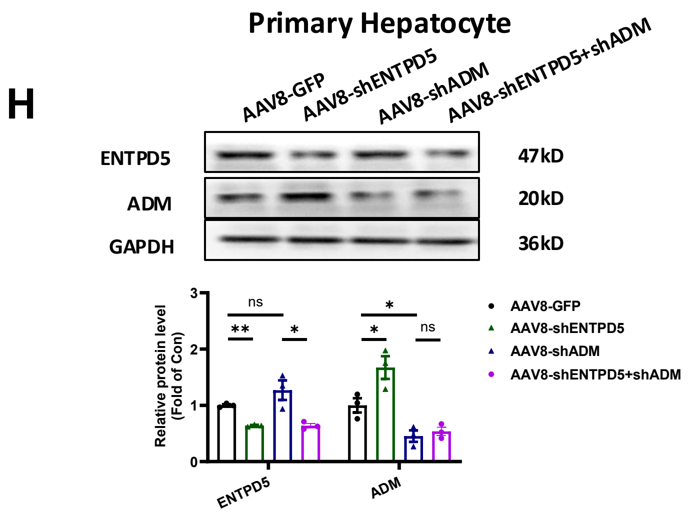

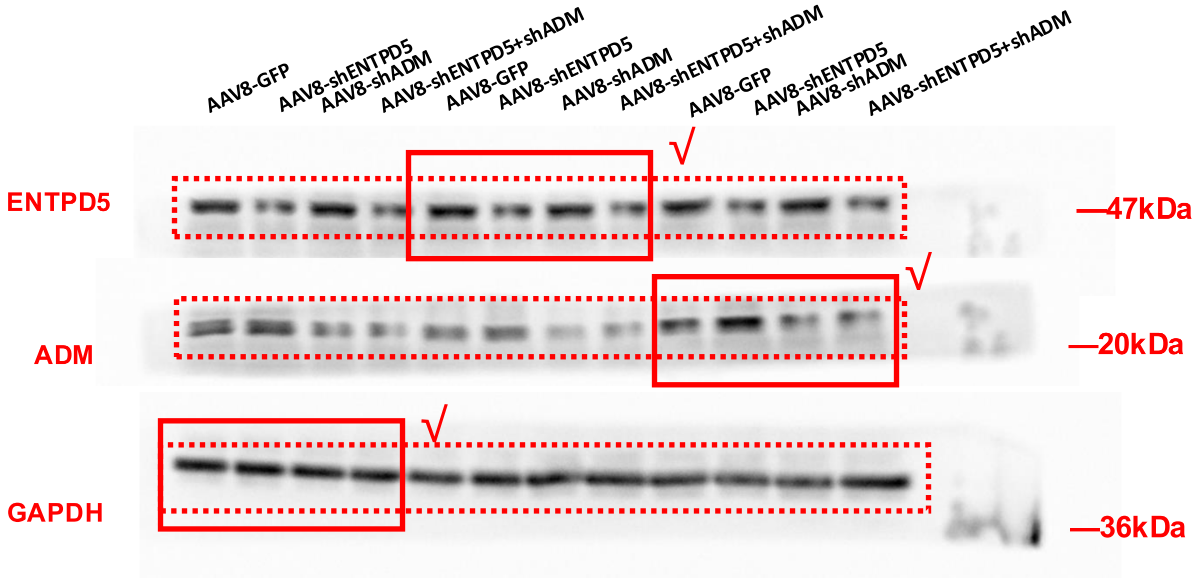

Full unedited gel for Suppl-figure.5I

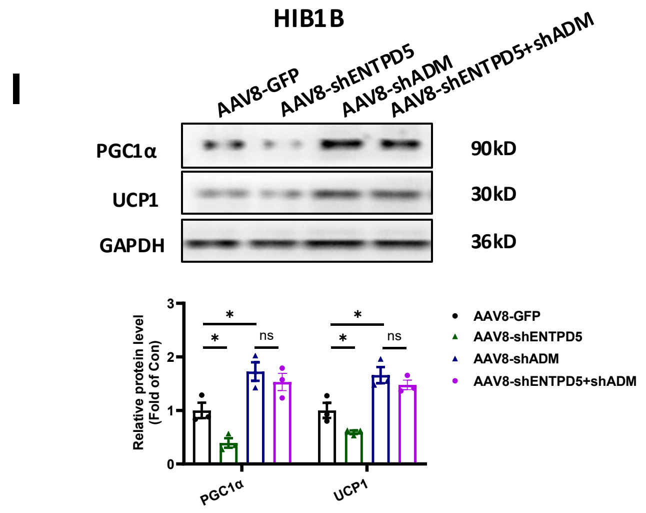

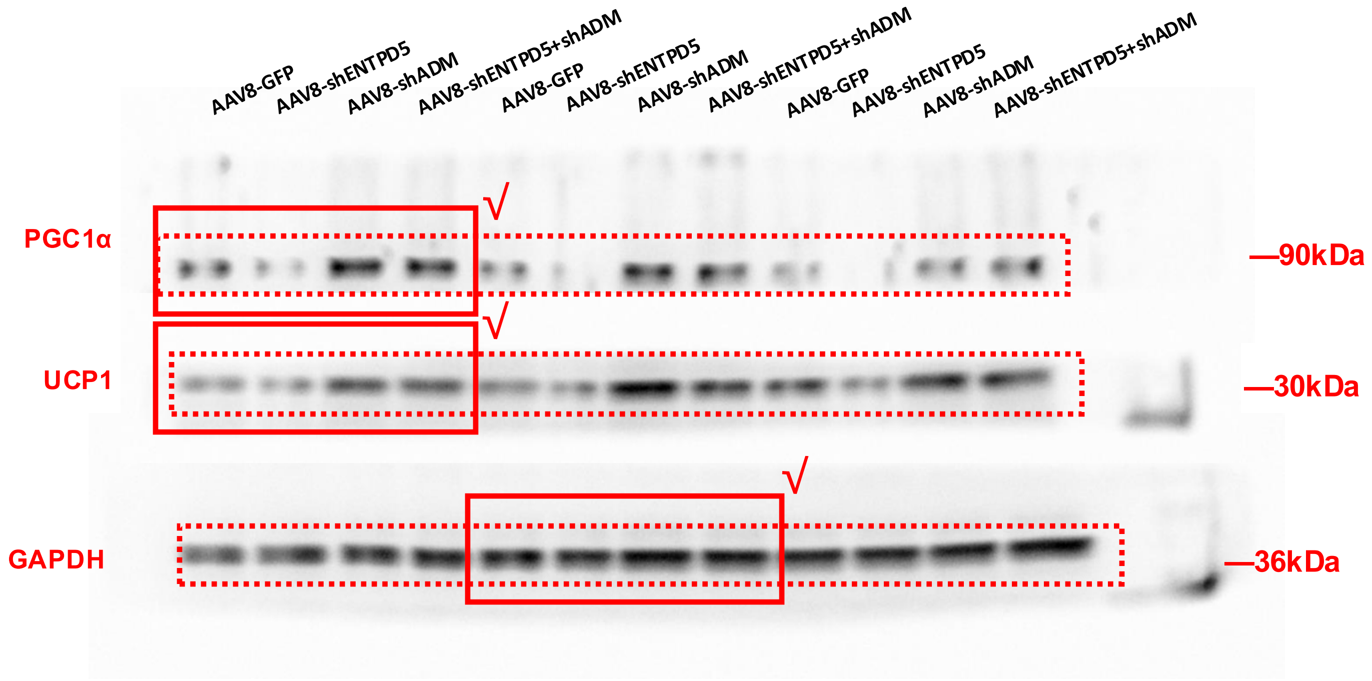

Full unedited gel for Fig. 7G ADM p-AKT AKT G6Pase PEPCK FASN GAPDH

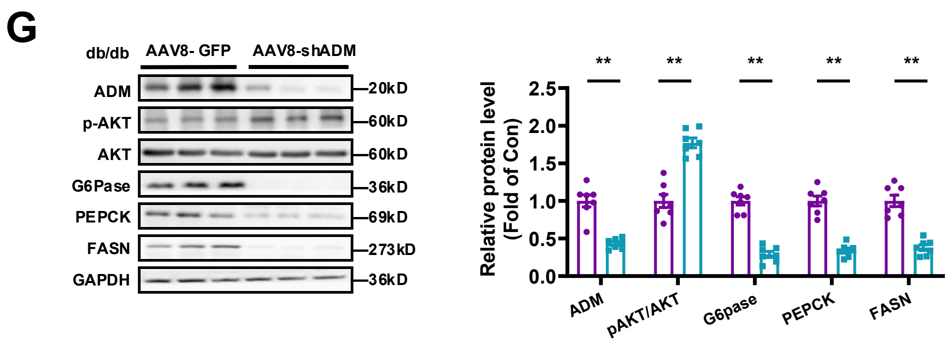

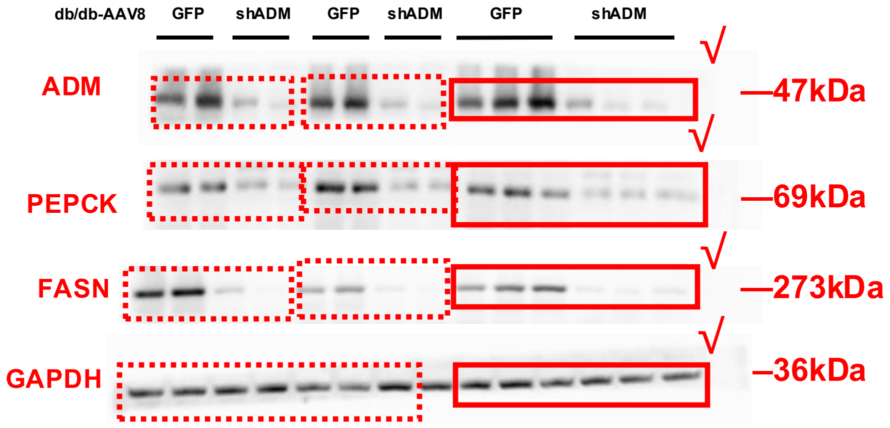

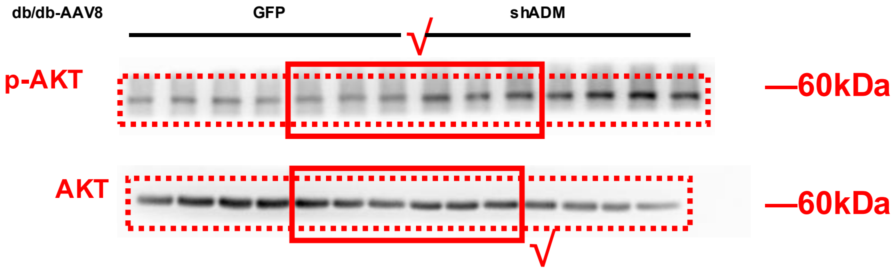

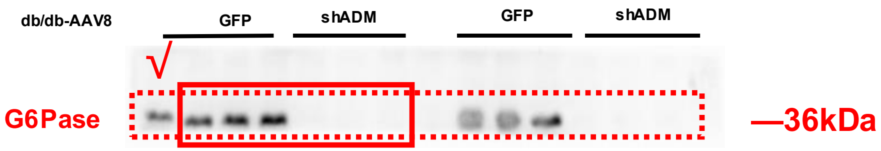

Full unedited gel for Fig. 7I UCP1 PGC1α GAPDH

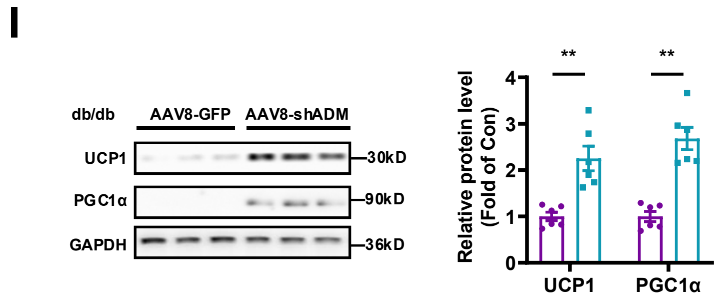

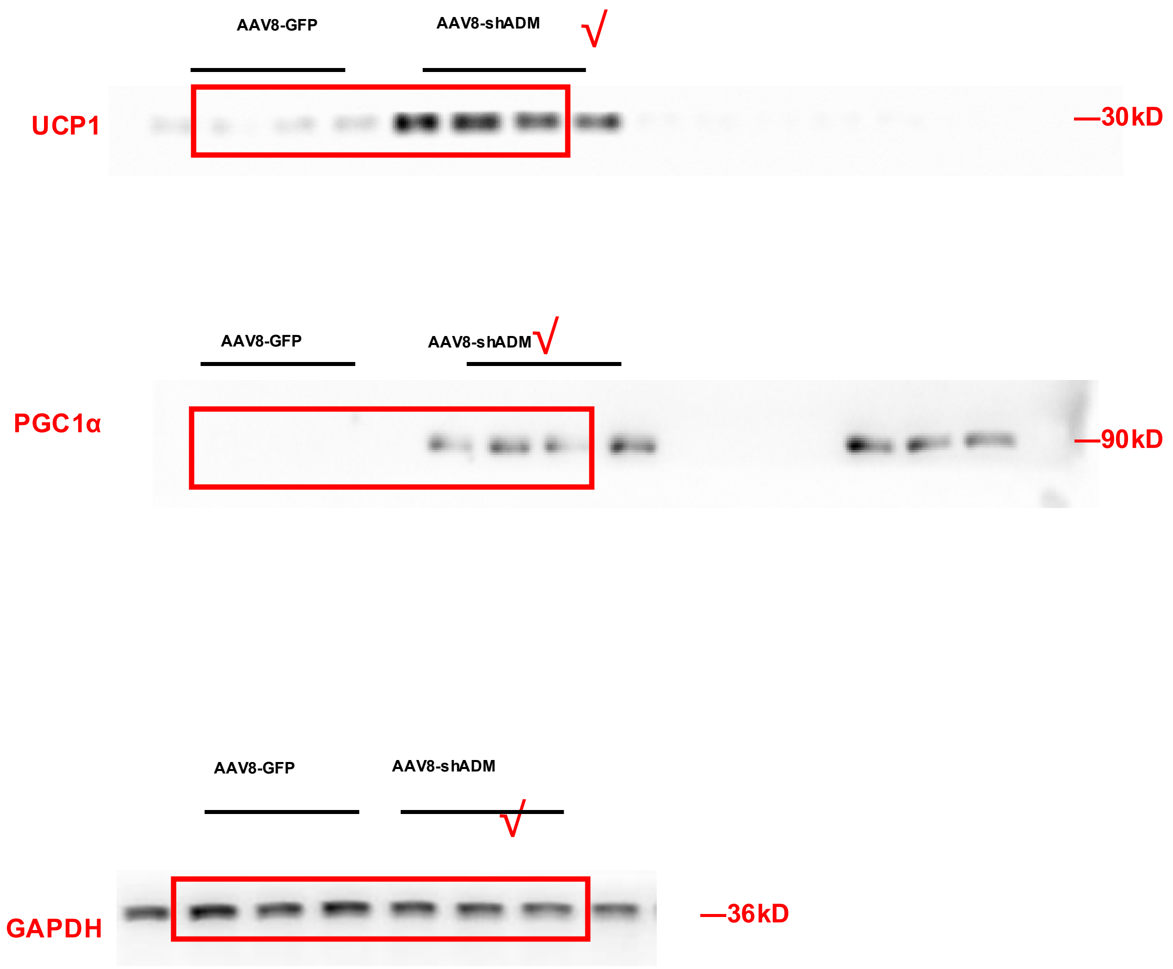

Full unedited gel for Fig. 7P UCP1 PGC1α GAPDH`

P

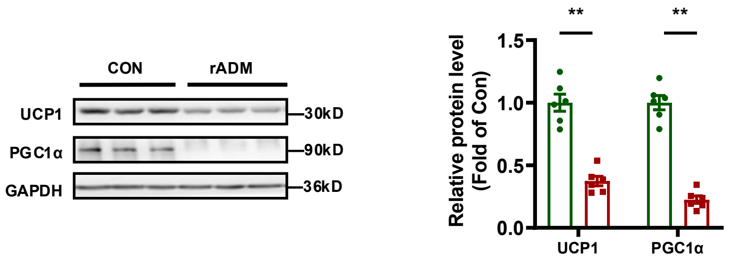

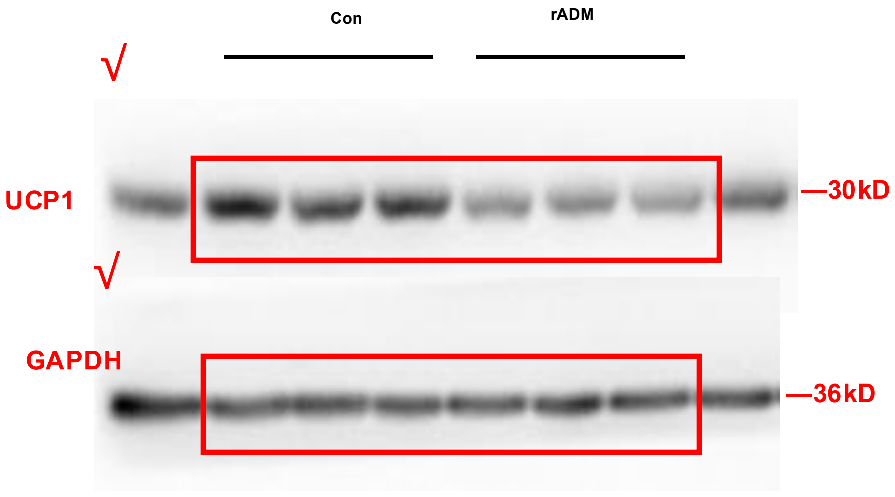

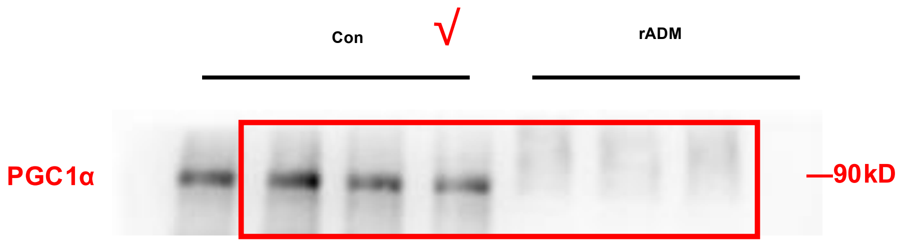

Full unedited gel for Fig.8D p-AKT AKT G6Pase PEPCK FASN ADM GAPDH

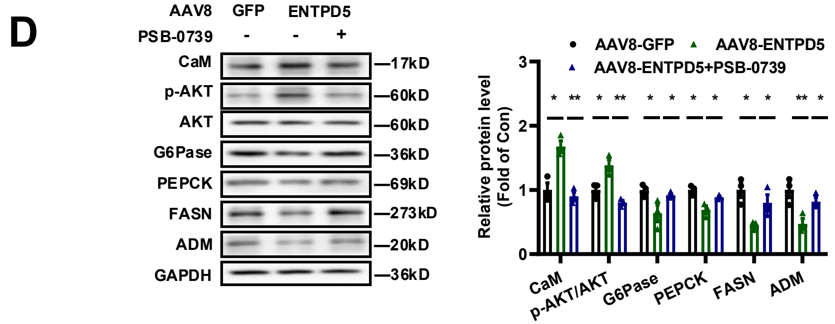

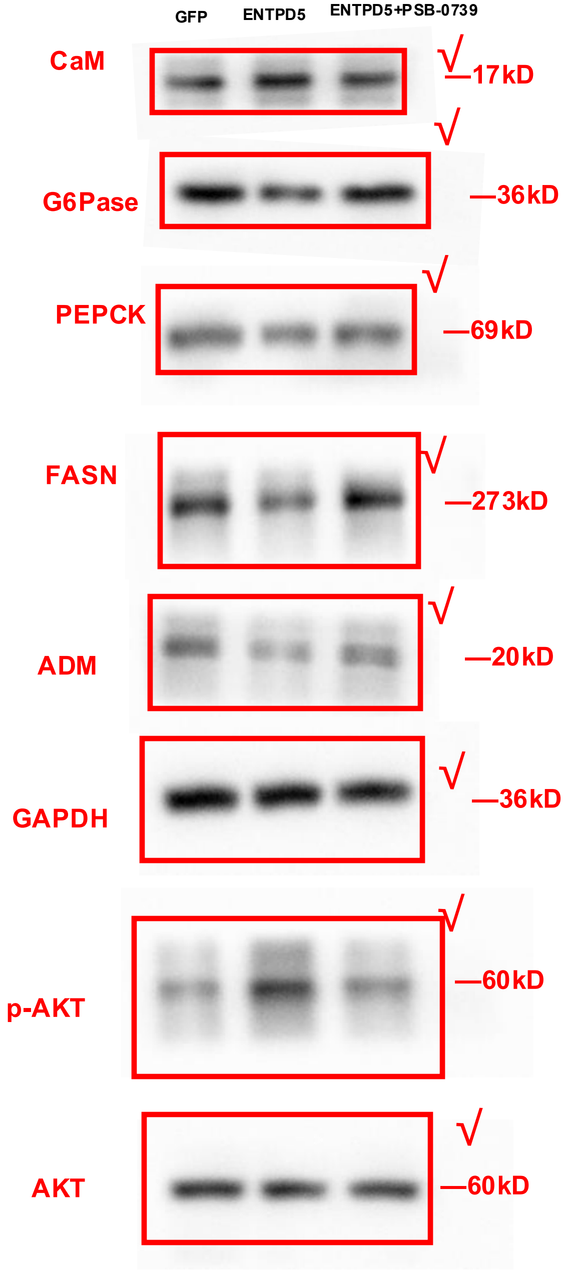

Full unedited gel for Fig. 8G UCP1 PGC1α GAPDH

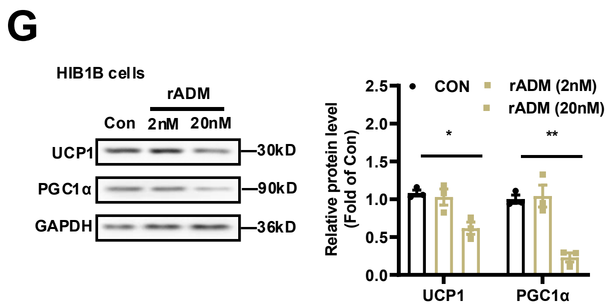

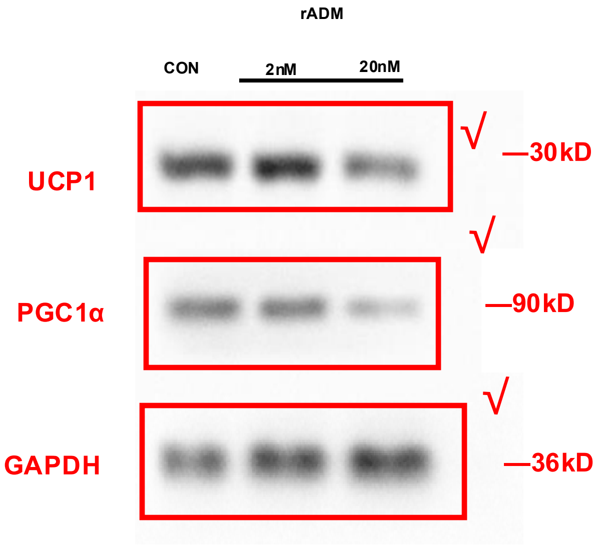

Full unedited gel for Fig. 8I ADM GAPDH UCP1 PGC1α GAPDH

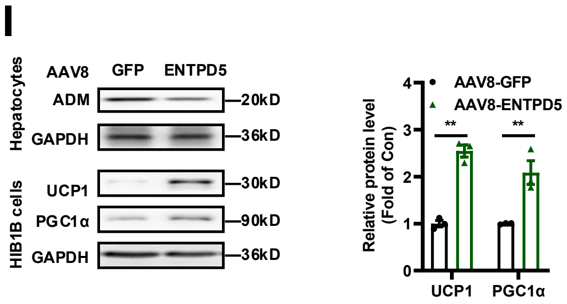

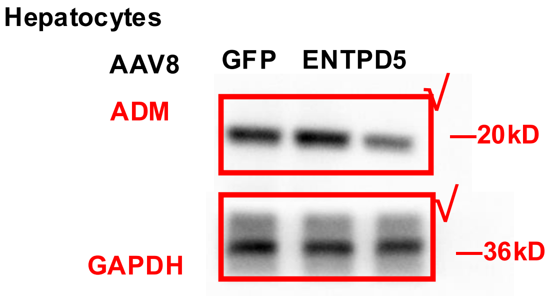

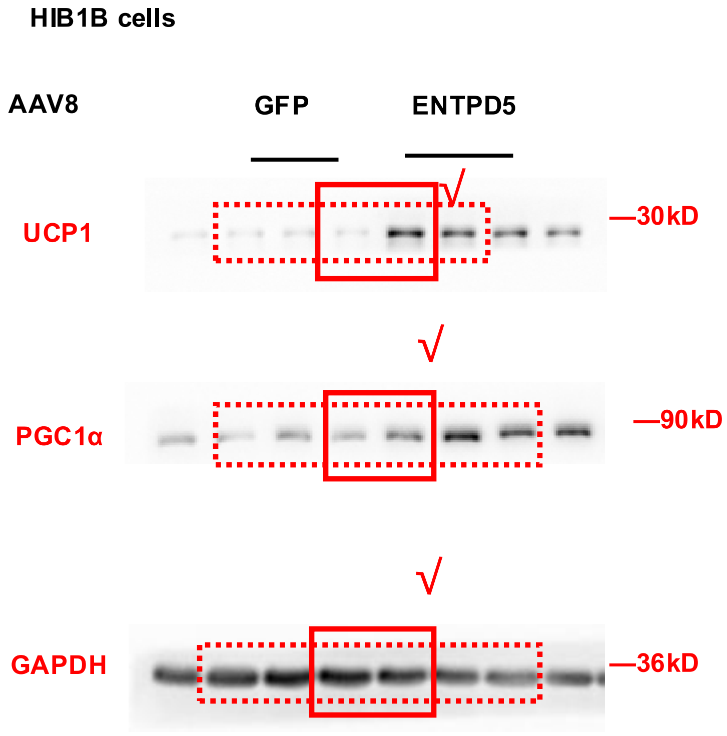

Full unedited gel for Fig. 8J ADM GAPDH UCP1 PGC1α GAPDH

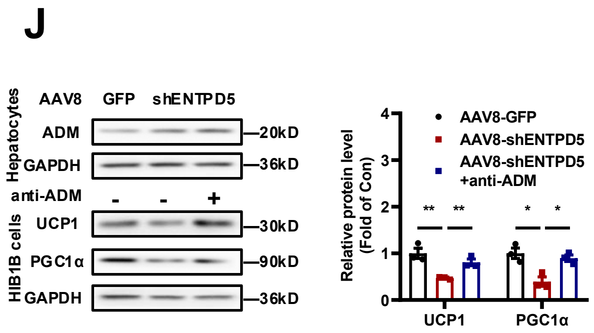

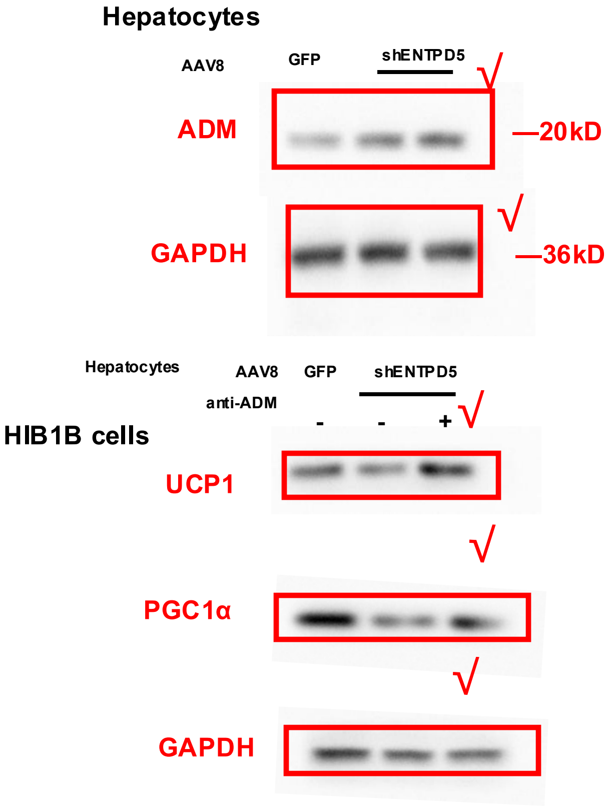

Full unedited gel for Fig. 8K ADM GAPDH UCP1 PGC1α GAPDH

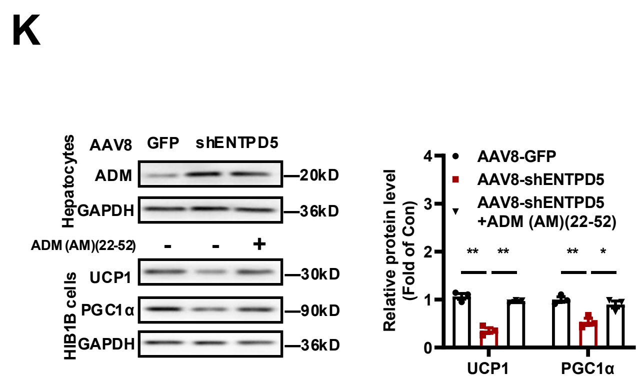

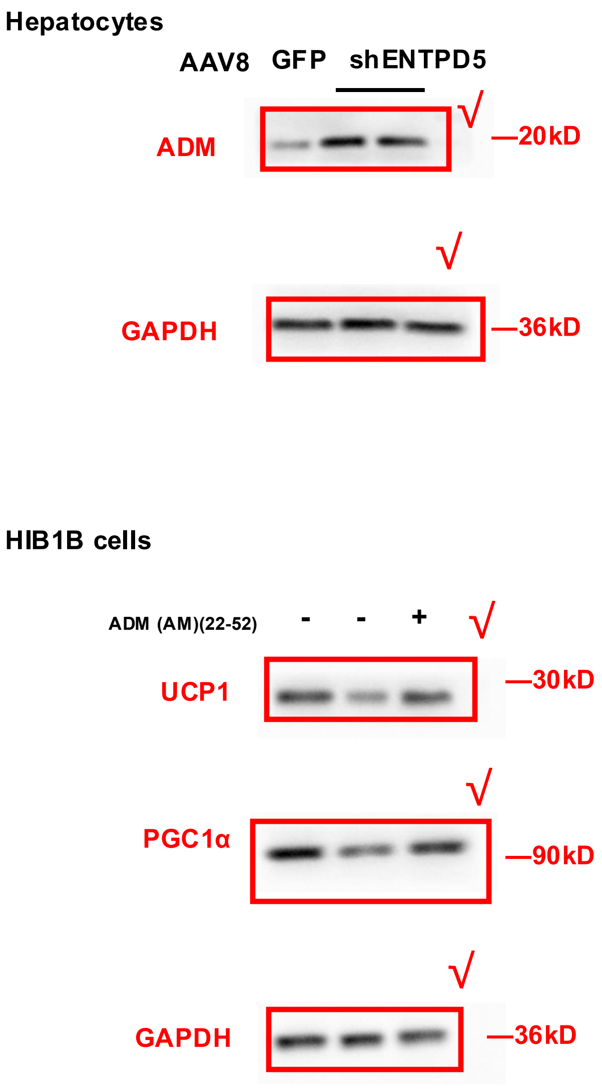

Full unedited gel for Fig. 8P CaM ENTPD5 MECP2 ADM GAPDH

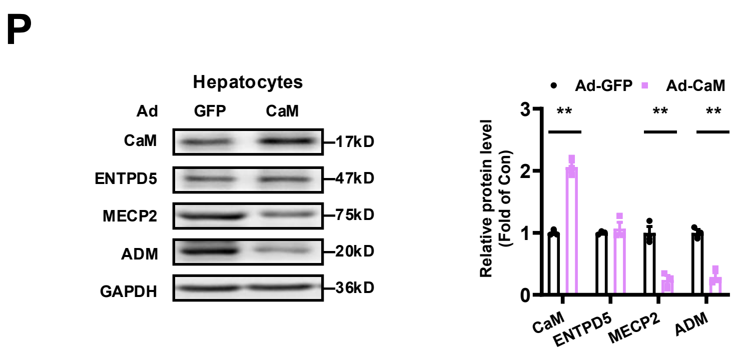

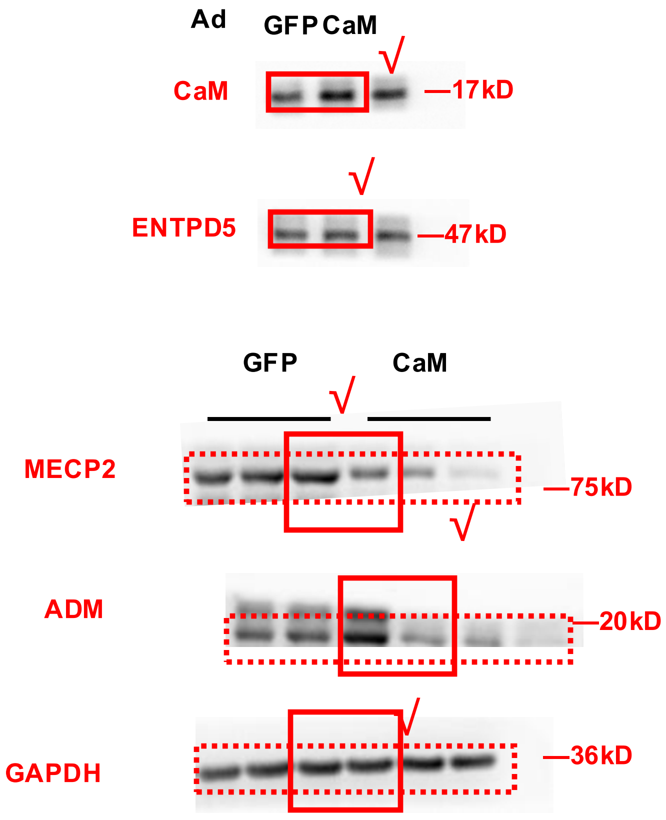

Full unedited gel for Fig. 8Q CaM p-JNK JNK p-AP1 AP1 MECP2 ADM GAPDH

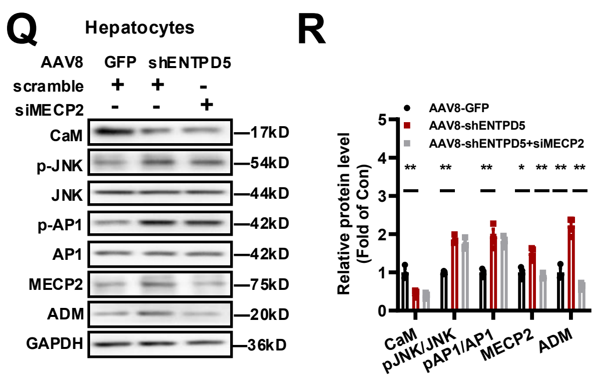

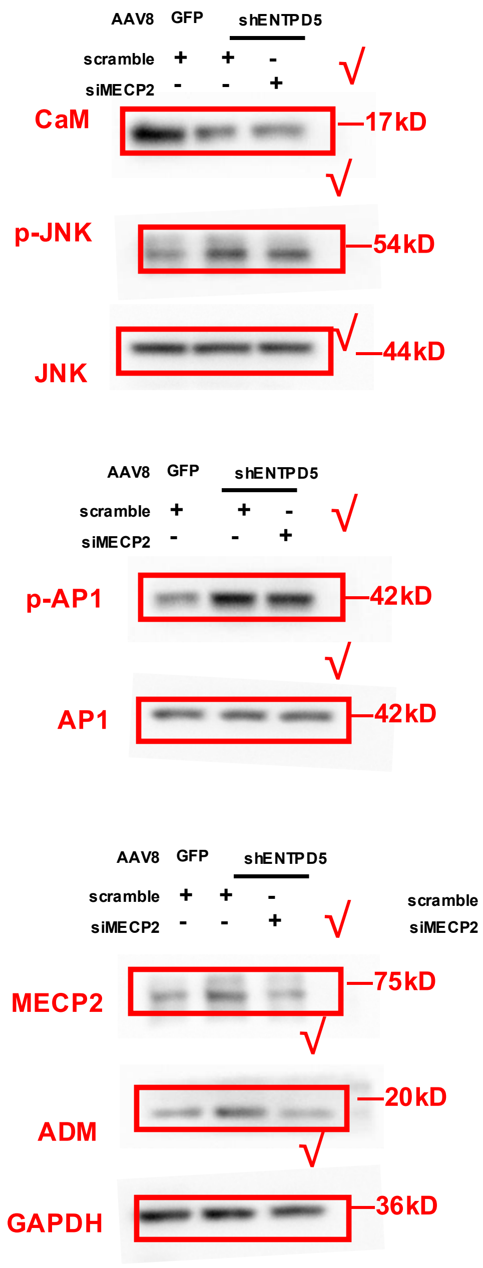

Full unedited gel for Fig. 8V UCP1 GAPDH

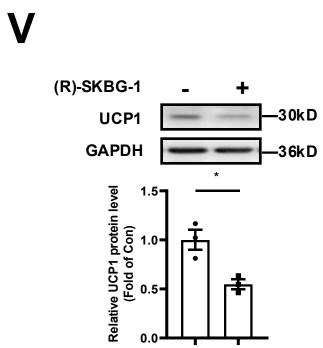

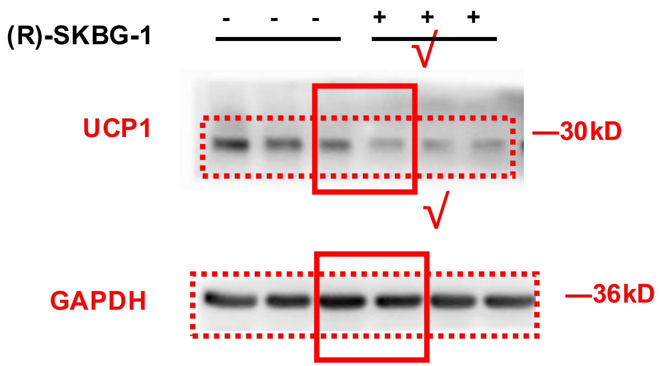

Full unedited gel for Suppl-figure.6F MECP2 GAPDH

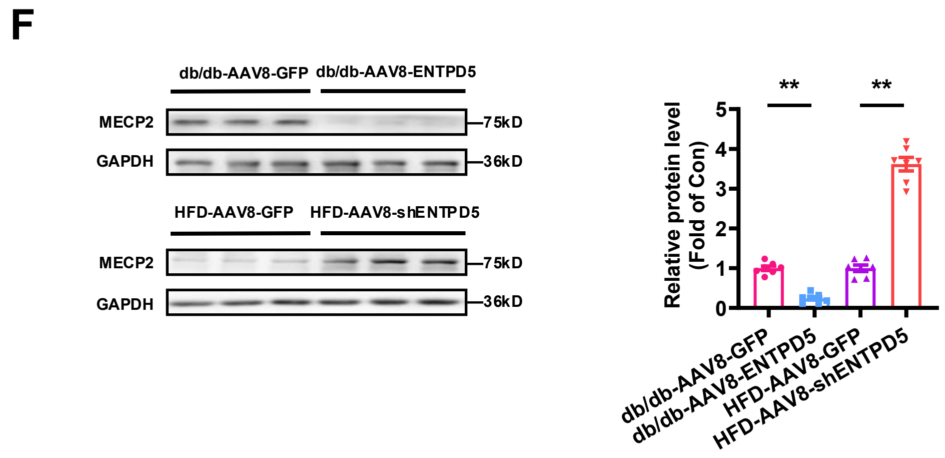

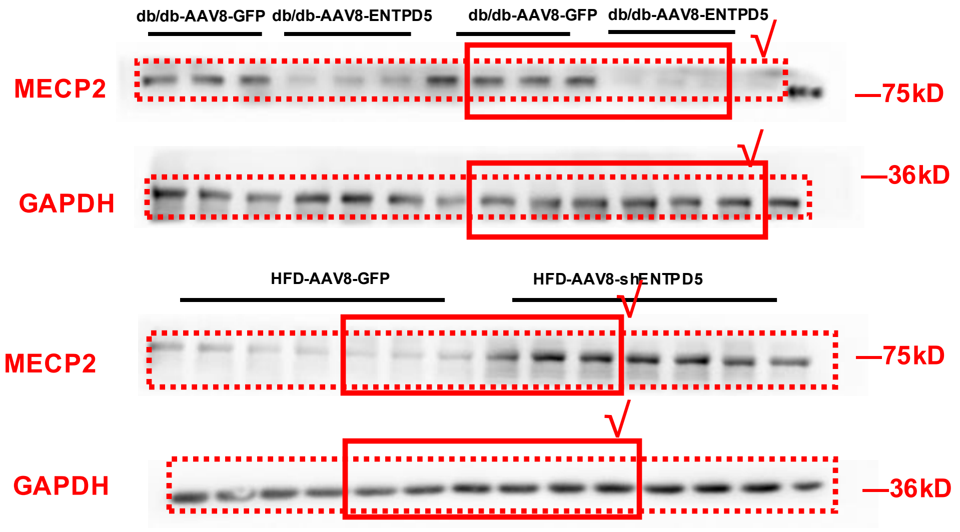

Full unedited gel for Suppl-figure.6G MECP2 GAPDH

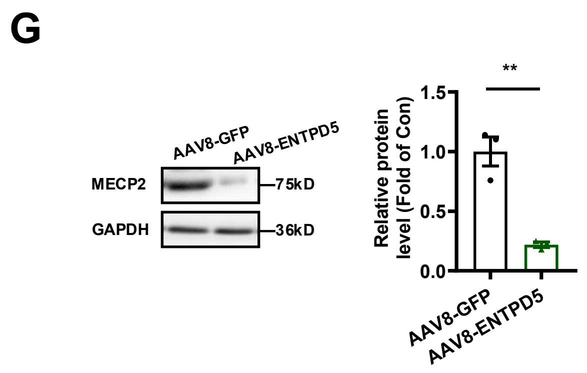

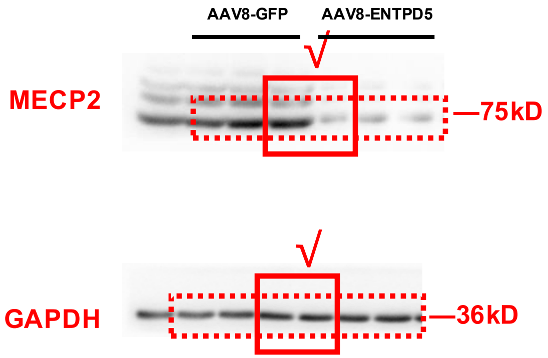

Full unedited gel for Suppl-figure.6H MECP2 GAPDH

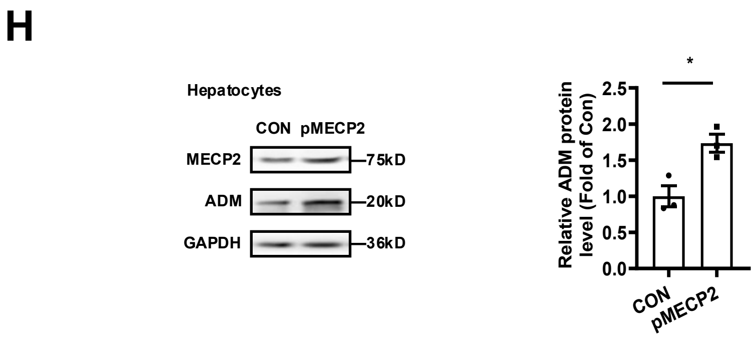

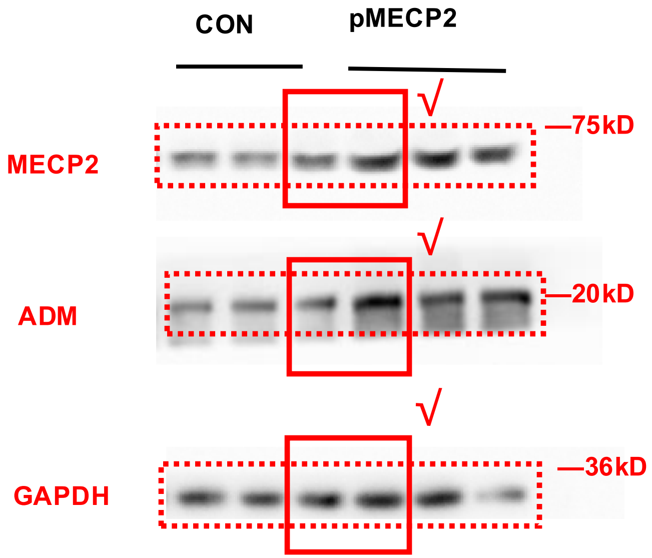

Full unedited gel for Suppl-figure.6I MECP2 GAPDH

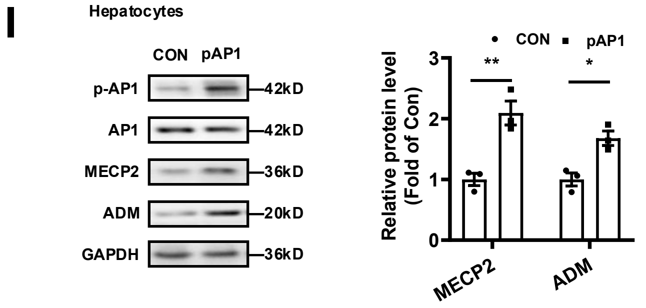

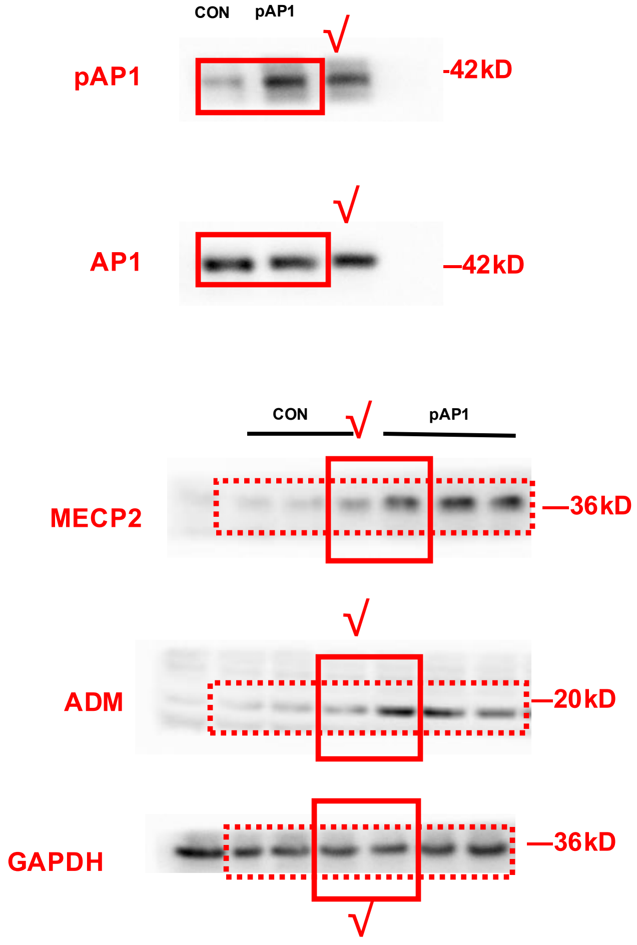

Full unedited gel for Suppl-figure.7F NONO GAPDH

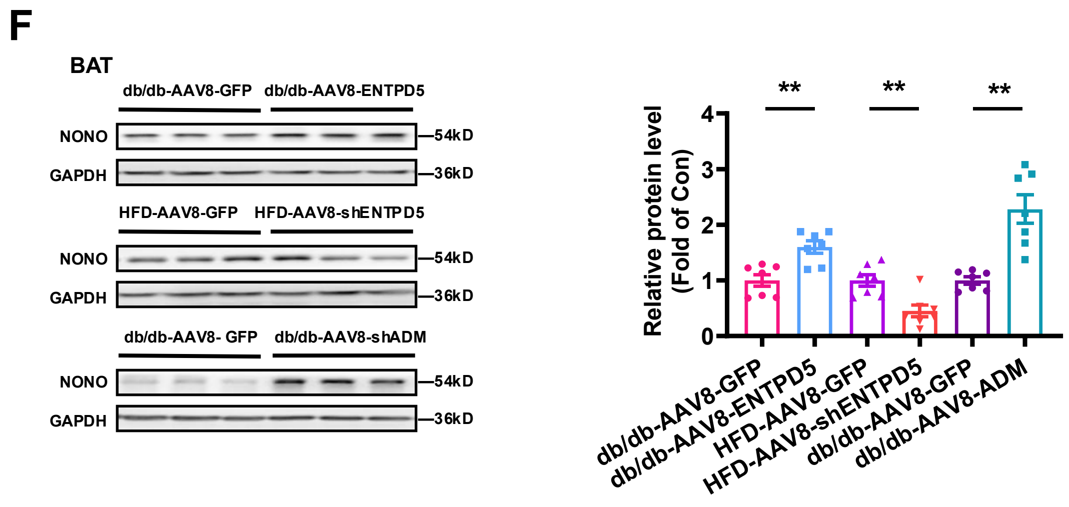

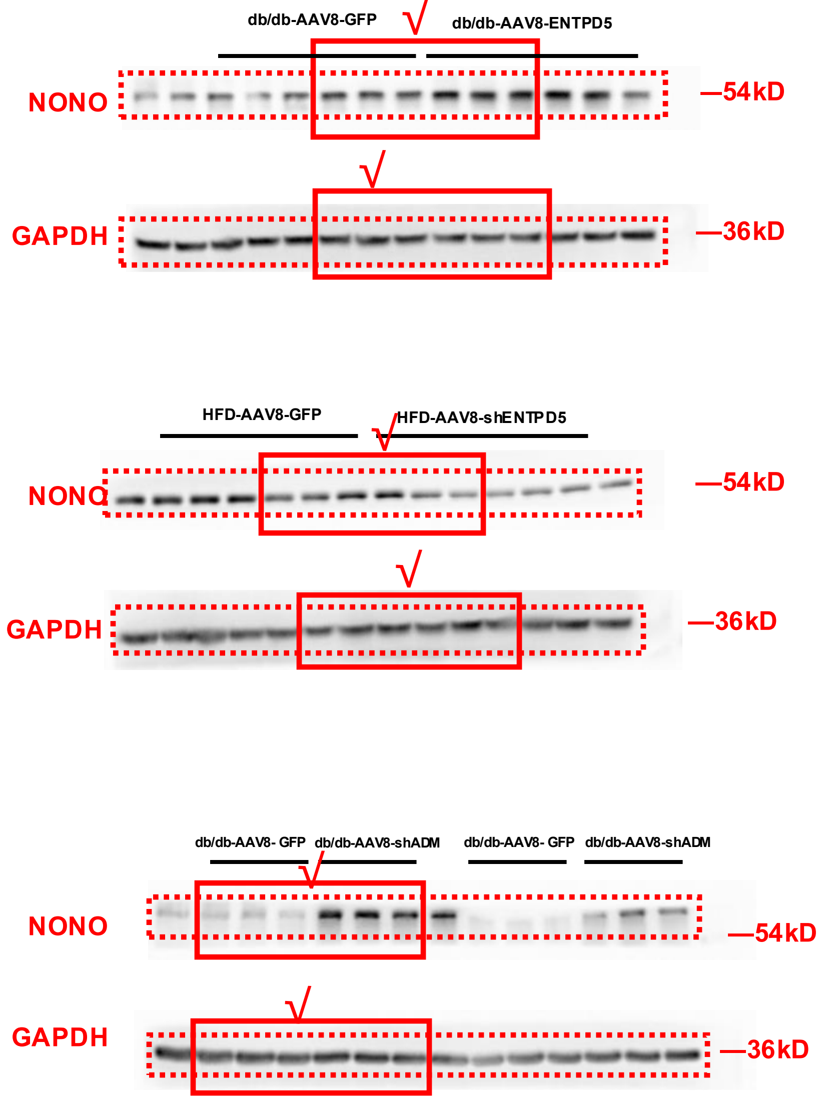

Full unedited gel for Suppl-figure. 7G NONO GAPDH

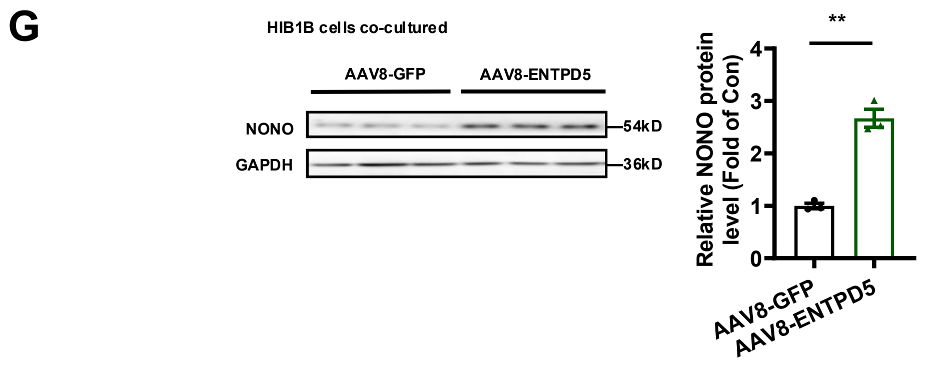

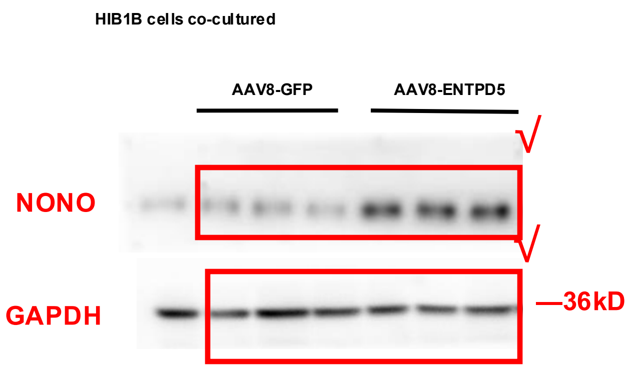

Full unedited gel for Suppl-figure. 7K NONO UCP1 GAPDH

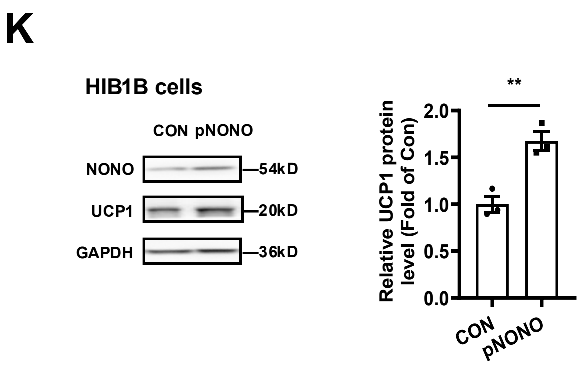

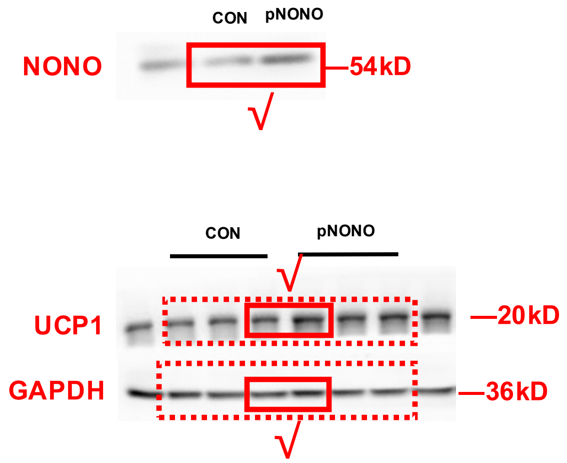

Full unedited gel for Suppl-figure. 8A NONO UCP1 GAPDH

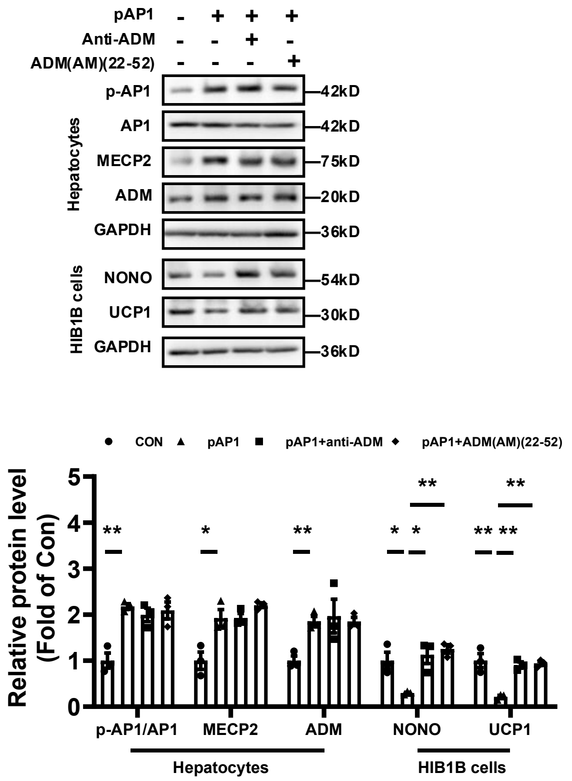

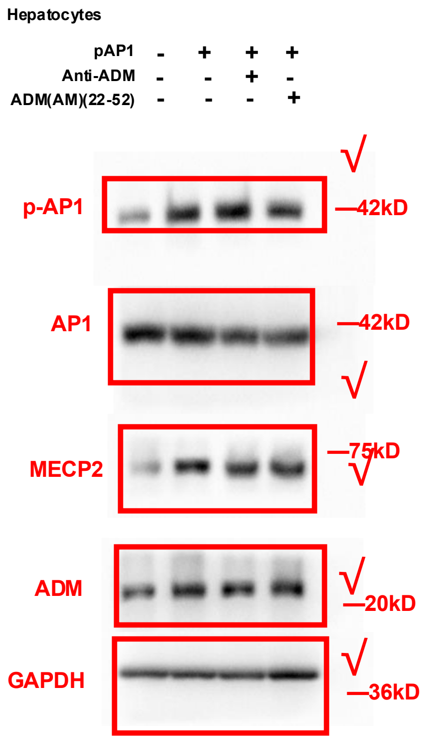

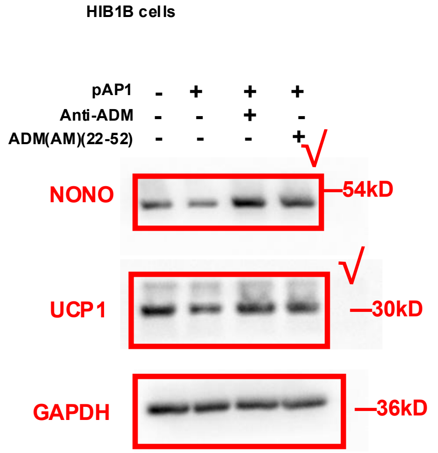

Full unedited gel for Suppl-figure. 8B NONO UCP1 GAPDH

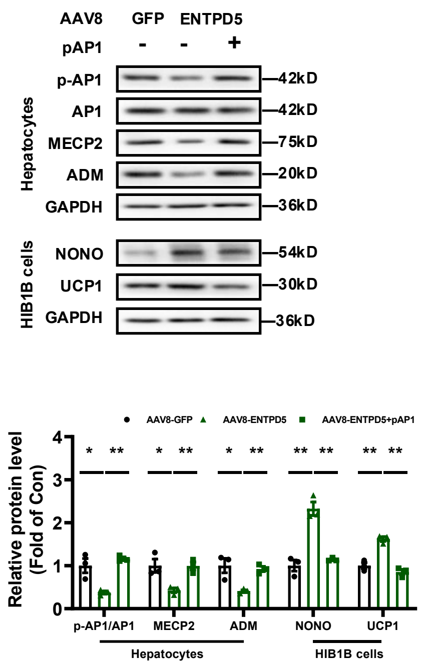

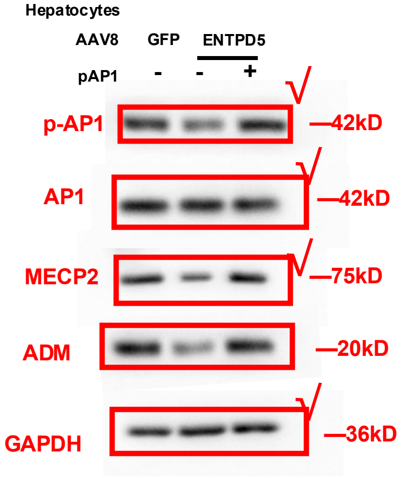

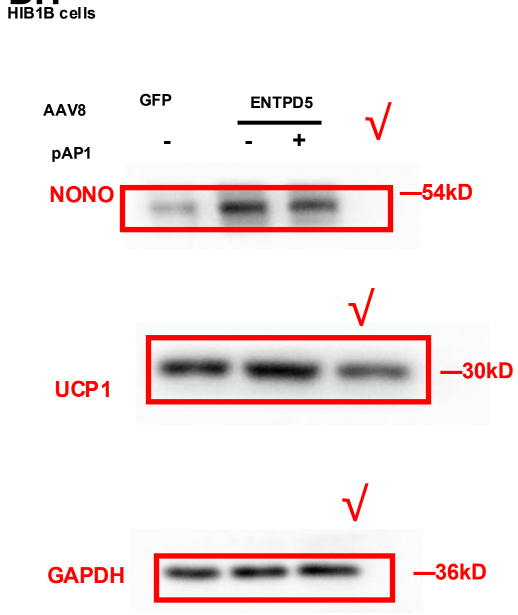

Full unedited gel for Suppl-figure. 8E NONO UCP1 GAPDH

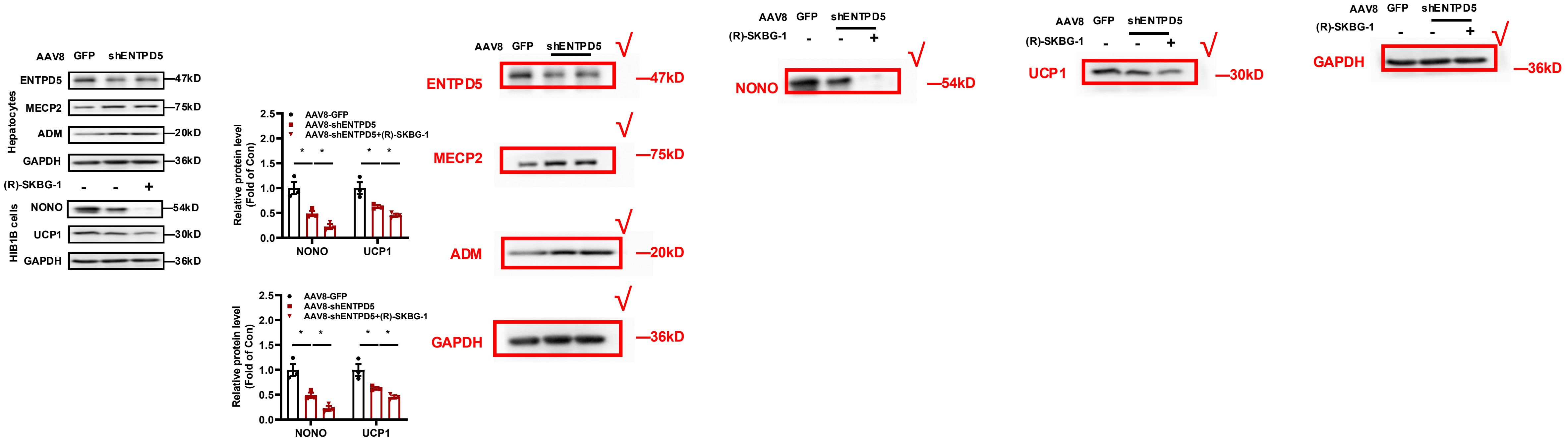

Full unedited gel for Suppl-figure. 8F NONO UCP1 GAPDH

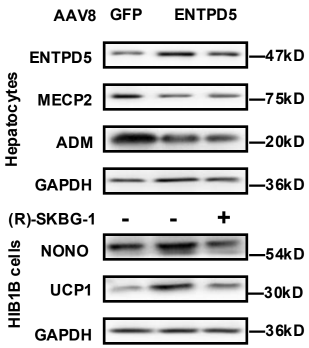

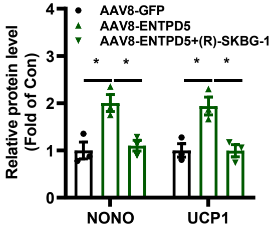

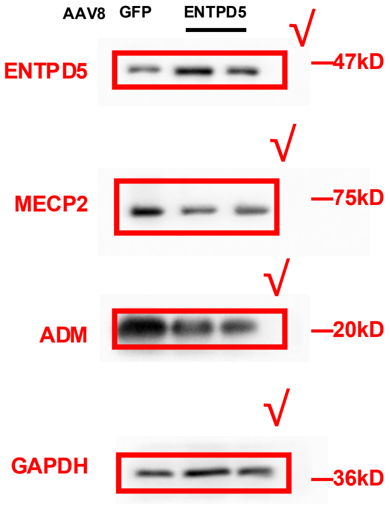

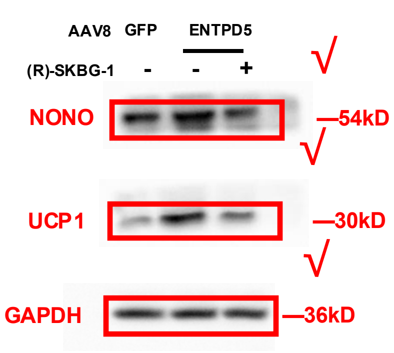

Full unedited gel for Suppl-figure. 8G NONO UCP1 GAPDH

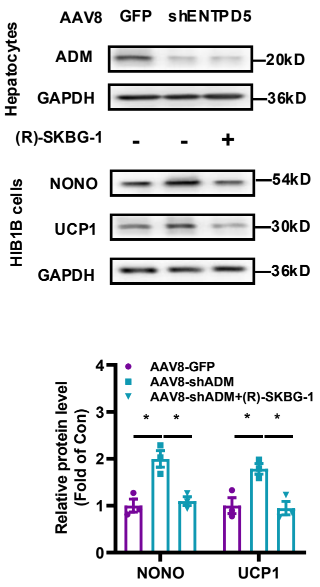

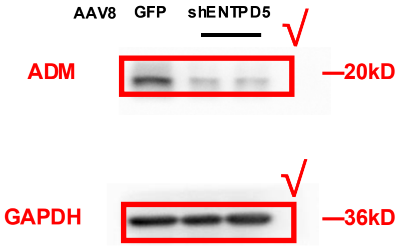

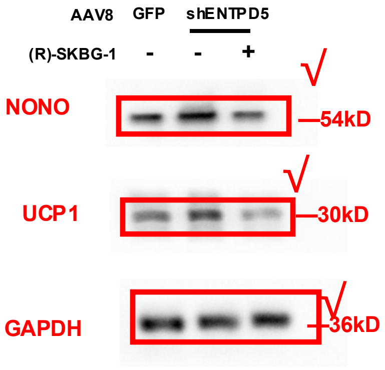

Supplement: Supplementary file 3 — Supporting Information [file ADVS-12-e03603-s002.pdf]
